# Effective control of large deletions after double-strand breaks by homology-directed repair and dsODN insertion

**DOI:** 10.1186/s13059-021-02462-4

**Published:** 2021-08-20

**Authors:** Wei Wen, Zi-Jun Quan, Si-Ang Li, Zhi-Xue Yang, Ya-Wen Fu, Feng Zhang, Guo-Hua Li, Mei Zhao, Meng-Di Yin, Jing Xu, Jian-Ping Zhang, Tao Cheng, Xiao-Bing Zhang

**Affiliations:** 1 State Key Laboratory of Experimental Hematology, National Clinical Research Center for Blood Diseases, Institute of Hematology & Blood Diseases Hospital, Chinese Academy of Medical Sciences & Peking Union Medical College, Tianjin, 300020 China; 2 Center for Stem Cell Medicine, Chinese Academy of Medical Sciences, Tianjin, China; 3 Department of Stem Cell & Regenerative Medicine, Peking Union Medical College, Tianjin, China

**Keywords:** CRISPR-Cas9, Genome editing, Large fragment deletions, Nanopore sequencing, Homology-directed repair (HDR), Non-homologous end joining (NHEJ), T cells, Hematopoietic stem and progenitor cells (HSPCs), Induced pluripotent stem cells (iPSCs)

## Abstract

**Background:**

After repairing double-strand breaks (DSBs) caused by CRISPR-Cas9 cleavage, genomic damage, such as large deletions, may have pathogenic consequences.

**Results:**

We show that large deletions are ubiquitous but are dependent on editing sites and cell types. Human primary T cells display more significant deletions than hematopoietic stem and progenitor cells (HSPCs), whereas we observe low levels in induced pluripotent stem cells (iPSCs). We find that the homology-directed repair (HDR) with single-stranded oligodeoxynucleotides (ssODNs) carrying short homology reduces the deletion damage by almost half, while adeno-associated virus (AAV) donors with long homology reduce large deletions by approximately 80%. In the absence of HDR, the insertion of a short double-stranded ODN by NHEJ reduces deletion indexes by about 60%.

**Conclusions:**

Timely bridging of broken ends by HDR and NHEJ vastly decreases the unintended consequences of dsDNA cleavage. These strategies can be harnessed in gene editing applications to attenuate unintended outcomes.

**Supplementary Information:**

The online version contains supplementary material available at 10.1186/s13059-021-02462-4.

## Background

Clustered regularly interspaced short palindromic repeats (CRISPR)-Cas9 is an RNA-guided DNA endonuclease system that targets specific genomic sequences [1]. Genome editing via the non-homologous end-joining (NHEJ) or homology-directed repair (HDR) after CRISPR-mediated double-stranded DNA (dsDNA) cleavage has transformed the field of cell and gene therapy. The potential applications of the CRISPR-Cas9 system for gene therapy in humans have been recognized and extensively investigated [2]. It is imperative to investigate genome editing’s unintended consequences thoroughly before its foray into the clinic.

Initial concerns about the off-target activity have been addressed by the development of sensitive detection methods [3–5], as well as modified Cas9 enzymes [6, 7] and improved delivery protocols [8] that limit this type of damage. Besides off-target effects, a combination of long-range PCR and third-generation sequencing technologies has led to the identification of frequent large fragment deletions (kilobase scale) and even complex genomic rearrangements at target sites of gene-edited cells and human embryos [9–13]. Compared with the PacBio platform, nanopore-based technologies detect DNA bases by monitoring a DNA molecule’s transit through a hole and measuring the variation in electric currents or optical signals. Nanopore sequencing, as commercialized by Oxford Nanopore Technologies (ONT), can produce high yields of very long 100+ kilobase (kb) reads [14]. Its portability, affordability, and speed in data production make it suitable for a comprehensive investigation of genome-editing associated large deletions [15].

Although DNA breaks introduced by Cas9 and single-guide RNA (sgRNA) frequently resolved into deletions extending over many kilobases in mouse and human cells, few studies explored the large-fragment deletions in clinically relevant cells. Careful evaluation of large-deletions in cell types of clinical significance, such as human primary T cells, hematopoietic stem and progenitor cells (HSPCs), and human induced pluripotent stem cells (iPSCs), is pivotal for their clinical translation. More importantly, developing strategies to attenuate this adverse effect is a prerequisite to further advancing this field. Here we hypothesized that exploitation of DNA damage repair pathways would effectively curtail DSB-induced large deletions.

For precise gene knock-in, templates with homology arms are often provided to guide HDR repair. The main HDR donor types are plasmid donors and single-stranded oligodeoxynucleotides (ssODNs) [16]. We have previously reported efficient HDR editing in cell lines and human iPSCs using plasmid donors [17–19]. However, plasmid donors often cause severe cytotoxicity due to the activation of the cytosolic DNA-sensing pathway [20]. Instead, adeno-associated virus (AAV) vectors have been successfully used as HDR templates [21, 22].

This study identifies large-fragment deletions in multiple loci in human T cells and HSPCs after CRISPR-Cas9 induced DSBs by long-range PCR and nanopore sequencing. Furthermore, for the first time, we show that AAV6 donor-mediated HDR almost abrogates, and NHEJ-mediated dsODN insertion attenuates large-fragment deletions, providing solutions to this type of adverse effect that hampers the clinical translation of genome editing-based therapy.

## Results

### Repair of double-strand breaks induced by CRISPR-Cas9 leads to large deletions in multiple cell types

**Fig. 1:**
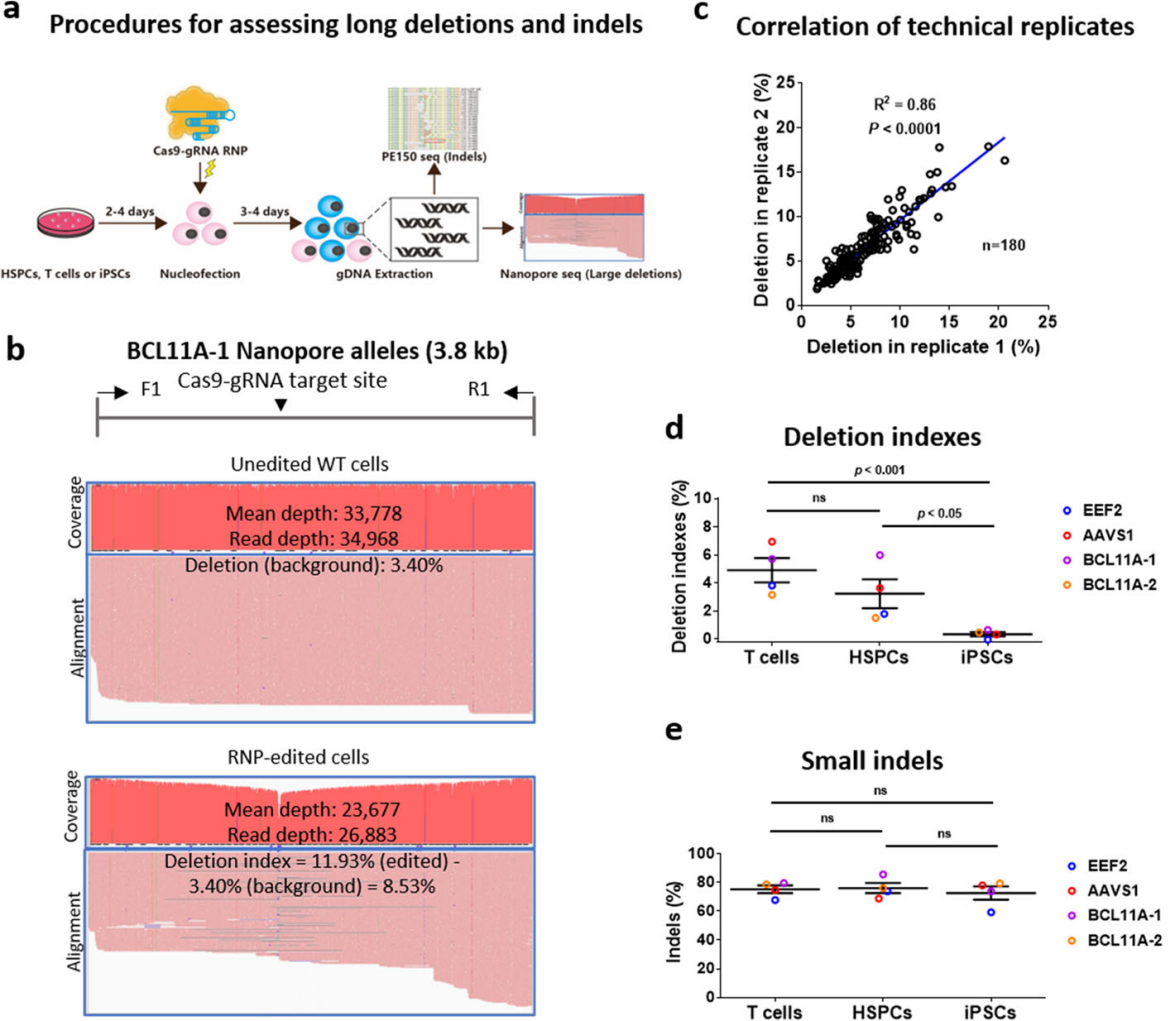
CRISPR-Cas9 RNP cleavage leads to large deletions. **a** Experimental design. Three types of human cells were edited with Cas9-gRNA RNPs. Editing efficiencies of small indels were assessed by Illumina amplicon sequencing and CRISPResso2 analysis. Large deletions were determined by long PCR and nanopore sequencing. **b** A representative of coverage and alignment of nanopore sequencing reads of the *BCL11A* amplicon. “Mean depth” and “Read depth” were analyzed by Samtools and Seqkit, respectively. Deletions were calculated by the formula (Read depth − Mean depth)/(Read depth). The deletion index was defined as deletion (%) of edited cells minus deletion (%) of unedited wildtype cells (background noise). A white area indicates an apparent deletion around the gRNA targeting sites in the coverage of alleles from RNP-edited cells. **c** Reproducibility of the deletion index data. The edited samples were PCR amplified with primers carrying different barcode sequences, followed by nanopore sequencing. The correlation of replicates indicates the reproducibility of this study. **d** Large deletion levels in three cell types. We used the deletion indexes to quantitate large deletions. **e** Frequencies of indels determined by NGS and CRISPResso2 analysis. For **d** and **e**, error bars represent the mean ± SEM of 4 experiments. The data in **d** and **e** were statistically analyzed by a two-way ANOVA test. Adjusted *p* values were indicated. “ns” means no significance (*p* > 0.05)

To identify significant genetic changes after CRISPR-mediated dsDNA cleavage (Fig. 1a), we PCR-amplified a 4- to 6-kb region flanking the Cas9-gRNA target sites at *EEF2*, *AAVS1*, and two *BCL11A* loci of three cell types (Additional file 1: Fig. S1a). We sequenced the barcoded PCR products via nanopore sequencing on PromethION. The data were demultiplexed using the grep command in the SeqKit bioinformatics packages [23] and aligned with reference amplicon sequences using Minimap2 [24] (Additional file 1: Fig. S1b). We first used ImageJ to define significant deletions (Additional file 1: Fig. S1c). To streamline the analysis, we used Samtools to determine the proportion of deletion mutations, which is defined as (read depth − mean depth) divided by (read depth). Data from four edited loci in wildtype (WT) cells showed a mean deletion of 3.3% (Fig. 1b and Additional file 1: Fig. S1d), which mostly reflects ONT sequencing errors (nucleotide deletions). The deletion indexes analyzed by Samtools and ImageJ showed an excellent linear correlation (*R*^2^ = 0.98) (Additional file 1: Fig. S1c). To obtain actual deletion mutation rates from editing groups, we used the metric of deletion index (deletion in editing group (%) − deletion in WT group (%)). We also assessed the reproducibility of long PCR and our data analysis strategy. We found that deletion indexes from technical replicates of amplicons primers with different barcodes correlated very well (*R*^2^ = 0.86, *P* < 0.0001) (Fig. 1c).

Using the above-established approach, we assessed the large fragment deletions after CRISPR-Cas9 targeting four loci in human primary T cells, cord blood CD34^+^ HSPCs, and iPSCs (Fig. 1a). We chose a gRNA to target the stop codon of the *EEF2* locus. *AAVS1* is a safe harbor in the genome. *BCL11A* is a gene therapy target site for hemoglobinopathy. Disruption of the expression of *BCL11A* can trigger *HBG* (γ-globin) gene activation. We chose a previously reported gRNA targeting *BCL11A* GATA motif (named BCL11A-1) [25], and also designed a gRNA to target *BCL11A* intron 2 (designated as BCL11A-2). To minimize off-target effects, we used Cas9-gRNA ribonucleoprotein (RNP) to edit human T cells, HSPCs, and iPSCs. Commercial tracrRNAs and crRNAs are chemically modified to have excellent stability [26]. We found an apparent deletion around the gRNA targeting sites in human T cells after editing (Fig. 1b). To a less extent, we also observed deletions in edited HSPCs. Of interest, iPSCs exhibited low-level deletions (Fig. 1d). This difference was not attributable to differential editing efficiency since indel frequencies were comparable in human primary T cells, HSPCs, and iPSCs (Fig. 1e).

In the above studies, we observed considerably lower deletions in iPSCs compared to T cells and HSPCs. We asked if cell death after electroporation of RNPs contributed to biased results in the three types of cells. We counted cell numbers 2 days after transfection relative to unmanipulated counterparts as a surrogate indicator of cell survival. No significant differences in viabilities were observed (Additional file 1: Fig. S3a). We further investigated if changes in the cell cycle played a part. We profiled the cell cycle using Pyronin Y and Hoechst 33342 co-staining 1 day after RNP delivery (Additional file 1: Fig. S3b, c). We observed no significant differences in G0/G1, S, and G2/M phases in HSPCs (Additional file 1: Fig. S3d), while a slight increase of T cells in the S phase and iPSCs in the G2/M phase. However, we noted considerably more iPSCs in the G2/M phase than T cells or HSPCs (~ 50% vs. 20%). This striking distinction might have partly contributed to ~ 5-fold lower deletions in edited iPSCs since the HDR is the prevailing pathway in cycling iPSCs [27]. Together, significant low-level deletion mutagenesis in iPSCs likely results from their intrinsic nature and transient low-level intracellular Cas9.

**Fig. 2:**
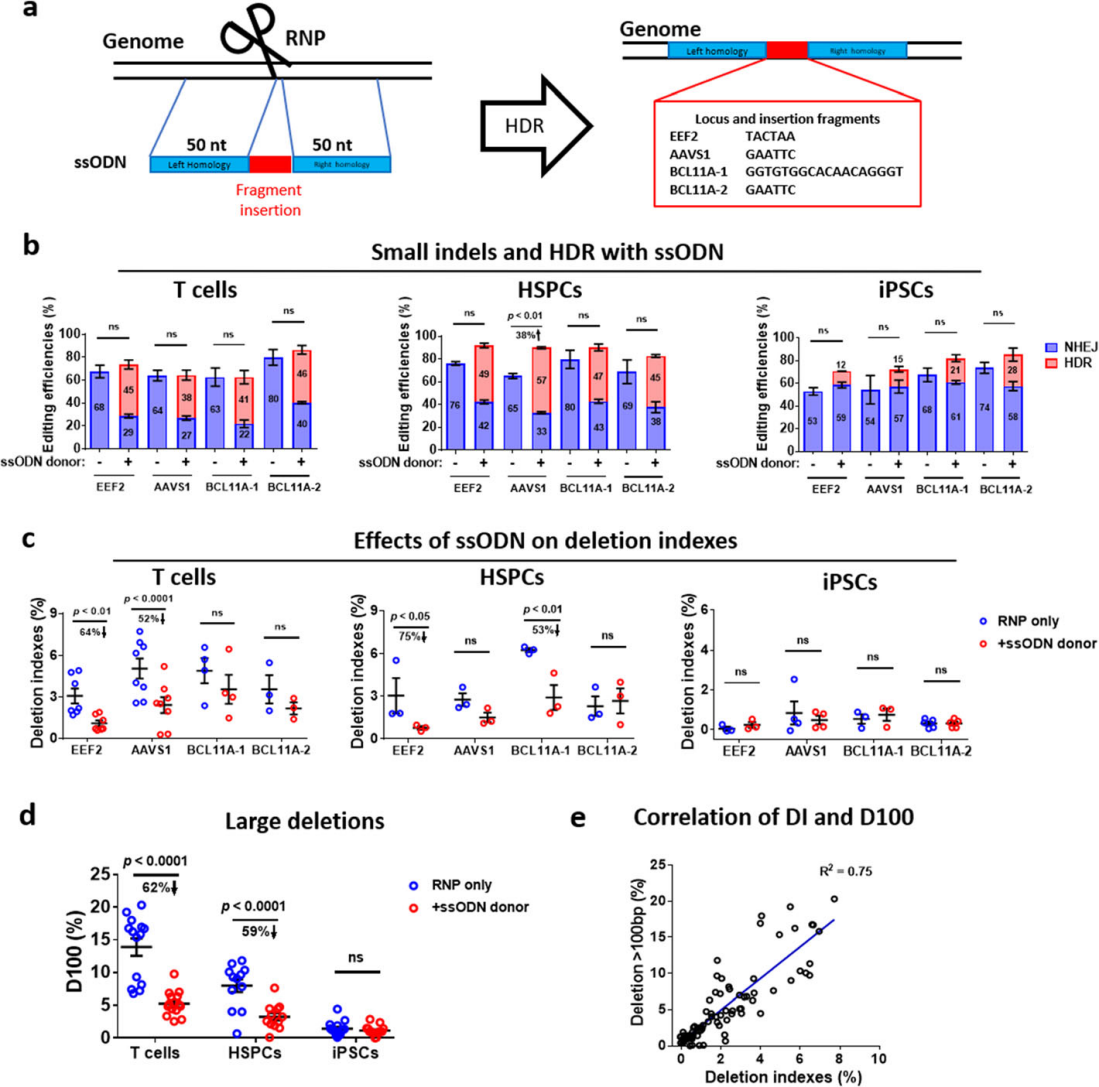
Single-stranded oligonucleotides mediated HDR editing attenuates large deletions. **a** Schematic for editing human T cells, HSPCs, and iPSCs with Cas9-gRNA RNPs and ssODN HDR donor templates. **b** Frequencies of small indels and HDR events after transfection of RNPs with or without ssODN donors in T cells, HSPCs, and iPSCs. Frequencies of indels (NHEJ) and HDR were determined by NGS and CRISPResso2 analysis 3 days after transfection. The numbers in the bars indicate the mean NHEJ or HDR efficiencies. **c** Frequencies of deletion indexes after RNP-ssODN editing in T cells, HSPCs, and iPSCs. The deletion indexes were determined by long-range PCR, ONT sequencing, alignment with references using Minimap2, and Samtools analysis. Error bars represent the mean ± SEM of *n* = 3–8 independent experiments. **d** Analysis of D100 in **c**. **e** Correlation of deletion indexes (DI) and deletion > 100 bp (D100) from data in **c**. The data in **b**, **c**, and **d** were statistically analyzed by a two-way ANOVA test. Adjusted *p* values were indicated. “ns” means no significance (*p* > 0.05)

**Fig. 3:**
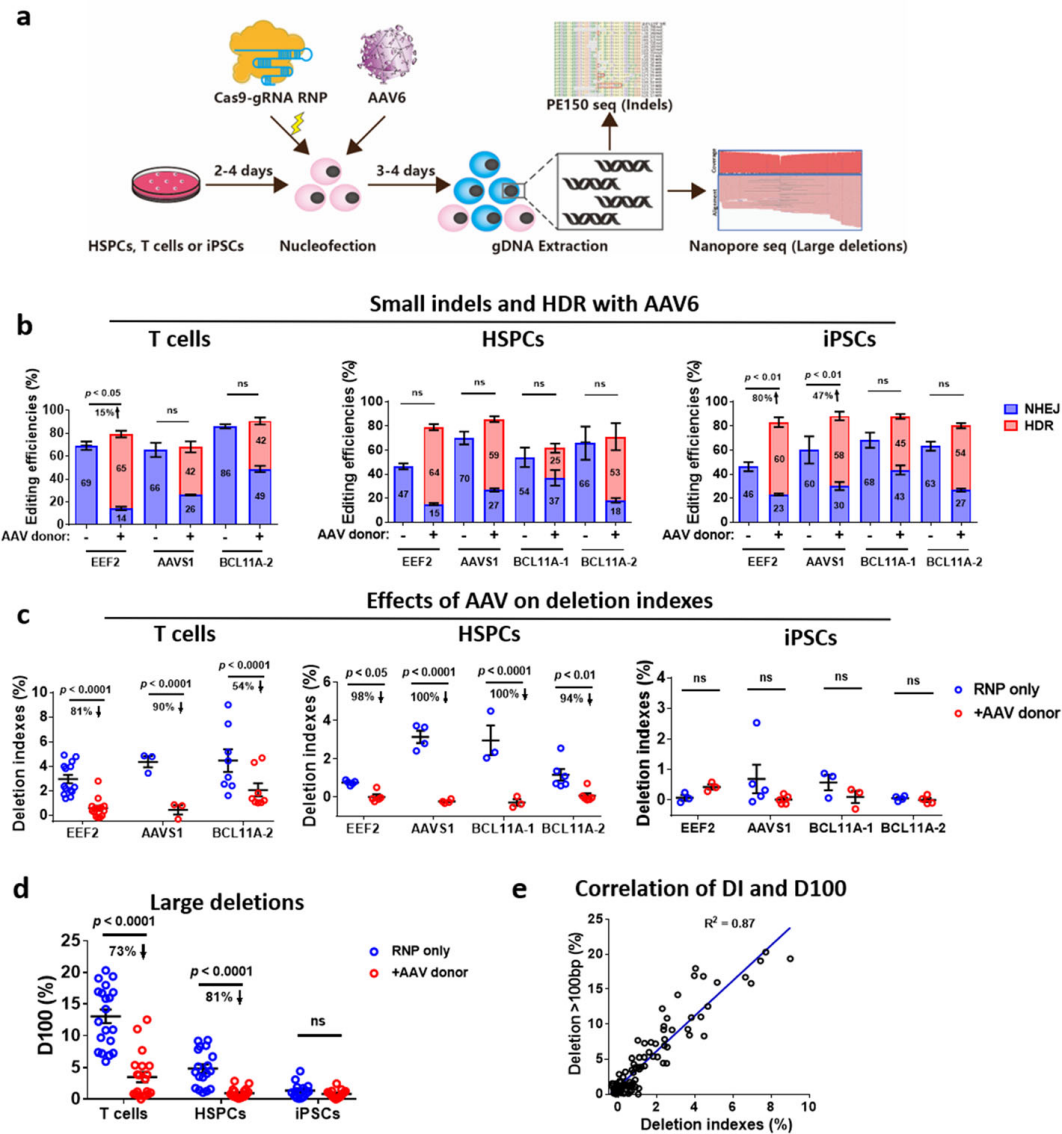
AAV6 donor-mediated HDR considerably reduces large deletions. **a** Experimental design for RNP-AAV6 editing. Three types of human cells were transfected with Cas9-gRNA RNP, followed by AAV transduction. Small indel (NHEJ) and HDR efficiencies were assessed by amplicon sequencing and CRISPResso2 analysis. Large deletions were determined by long PCR, nanopore sequencing, Minimap2 alignment, and Samtools analysis. **b** Frequencies of small indels and HDR as determined by amplicon sequencing. The numbers in the bars indicate the mean NHEJ or HDR efficiencies. **c** Frequencies of deletion indexes. Deletion indexes are deletions in edited cells subtracted by deletions (background noise) in wildtype samples. Error bars represent mean ± SEM of *n* = 3–12 independent experiments. **d** Analysis of D100 in **c**. **e** Correlation of deletion indexes (DI) and deletion > 100 bp (D100) from data in **c**. The data in **b**, **c**, and **d** were statistically analyzed by a two-way ANOVA test. Adjusted *p* values were indicated. "ns" means no significance (*p* > 0.05)

To quantify the levels of large deletions precisely, we used the deletion index, whereas previous studies defined removing fragments of over 100 bp (D100) as large deletions [12, 28]. Therefore, we extended the analysis by calculating alleles with deletions of over 100, 500, 1000, 1500, or 2000 bp (Additional file 1: Fig. S2). We found that over 80% of large deletions were 100–1000 bp in length, while D2000 was 0.1% or lower (Additional file 1: Fig. S2a-c). Furthermore, even though D100 was more sensitive than deletion index (DI) in assessing large deletions, deletion indexes correlated excellently with D100 (Figs. 2e and 3e, and Additional file 1: Fig. S8c). We thus decided to report DI in detail and also summarized D100 data for comparison.

### Single-stranded oligonucleotides mediated HDR knock-in decreases large deletions by 40%

Having validated large deletions after CRISPR targeting, we asked whether providing ssODN HDR donors can decrease deletions. We used modified ssODN donors with ~ 50 bp homology to guide HDR insertion of a 6-bp or 18-bp fragment at the cleavage site (Fig. 2a). We analyzed indels and HDR editings 72 h after the delivery of RNP and ssODN by PCR amplification of sequences flanking the target followed by Illumina sequencing. The presence of ssODN donors led to 38–46% HDR knock-in efficiencies in four individual targeting sites in T cells (Fig. 2b). The total editing events (NHEJ+HDR) were similar in the presence or absence of ssODNs (Fig. 2b). However, the ssODN HDR editing led to significantly decreased deletion indexes at the *EEF2* (an ~ 64% decline) and *AAVS1* (an ~ 52% reduction) loci (Fig. 2c). At the two *BCL11A* loci, the effects were less pronounced. Still, the ssODN HDR donor led to a lower deletion in T cells (Fig. 2c).

Next, we analyzed the ssODN-based HDR effects on deletion mutations in human cord blood HSPCs. The presence of ssODNs led to high HDR efficiencies (45–57%) in HSPCs (Fig. 2b) compared with T cells, accompanied by an increase of total editing efficiencies from 60 to 80% even to 80–90% (Fig. 2b). At three out of four loci, the deletion levels dwindled in edited HSPCs, with a 75% and 53% decrease at *EEF2* and *BCL11A-1*, respectively (Fig. 2c). Compared to T cells and HSPCs, iPSCs exhibited lowered ssODN-mediated HDR efficiencies, ranging from 12% to 28% (Fig. 2b). We also did not detect significant changes in deletion indexes in the absence or presence of ssODN in iPSCs (Fig. 2c). We speculated that low-level deletions might explain this observation in iPSCs. We aggregated all the ssODN editing data of T cells and HSPCs and concluded that ssODN-HDR editing led to an average of 40% decrease in deletion indexes (DI) (Additional file 1: Fig. S8a-b). Similarly, the ssODN donor leads to ~ 60% reduction of large deletions in T cells and HSPCs (Fig. 2d). Again, D100 was well associated with DI (Fig. 2e).

### AAV6 donor mediated HDR considerably reduces large deletions

Adeno-associated virus serum type 6 (AAV6) as an HDR donor has achieved impressive results in the genome editing of human T cells, hematopoietic cells, and iPSCs [29]. We attempted to assess whether AAV6 donors with ~ 600 bp homology instead of ssODN donors with 50 bp homology significantly affect large fragment deletion mutations. To facilitate the analysis of HDR and indels, we designed AAV6 HDR vectors to guide an 8 or 15 bp insertion at the Cas9-gRNA RNP cleavage site at *AAVS1* and *BCL11A* or insertion of a promoter-less mNeonGreen reporter at *EEF2* stop codon (Additional file 1: Fig. S4a). After the transfection of Cas9-gRNA RNP, cells were cultured in the absence or presence of AAV6 HDR donors (Fig. 3a). The knock-in efficiency of the promoter-less AAV-mNeonGreen HDR donor at the *EEF2* stop codon was ~ 65% in T cells as determined by FACS (Fig. 3b). As a control, this donor’s direct insertion at *AAVS1* or *BCL11A* locus showed 0% mNeonGreen^+^ cells (Additional file 1: Fig. S4b). AAV-mediated insertion of a short fragment in either *AAVS1* or *BCL11A* showed ~ 40% HDR efficiencies (Fig. 3b). The addition of AAV6 donors slightly increased total editings at *EEF2* (1.15-fold change), but had no apparent differences in *AAVS1* (1.04-fold), and *BCL11A* (1.07-fold) loci in T cells (Fig. 3b). However, AAV-HDR editings showed 81%, 90%, and 54% reduction in deletion indexes at *EEF2*, *AAVS1*, and *BCL11A*, respectively (Fig. 3c). The AAV6 donor mediated a significant decrease in large deletions in all three loci in T cells (Fig. 3c, d). At the *EEF2* locus, rather than a short fragment knock-in, AAV6 mediated a mNeonGreen reporter insertion. To determine whether the HDR alleles in the dataset artificially decrease the deletion indexes, we analyzed data after depletion of HDR alleles (Additional file 1: Fig. S5a, b). We found no significant differences between the reads with HDR alleles and reads without HDR alleles (Additional file 1: Fig. S5c, d). These data suggest that our analytical strategy precisely captures the deletion levels. To further consolidate AAV6-HDR donors’ role in reducing large fragment deletions, we evaluated AAV's dosage effects. As expected, with the increase of multiplicity of infection (MOI) from 1000 to 10,000, the HDR events steadily increased but no significant changes of total editing efficiencies at both *EEF2* and *AAVS1* loci (Additional file 1: Fig. S4c), accompanied by a considerable reduction of deletion indexes from ~ 2 to ~ 1% (Additional file 1: Fig. S4d).

Next, we assessed the effect of AAV-based HDR on the deletion indexes of edited HSPCs. The HDR efficiencies were 64%, 59%, 25%, and 53% at *EEF2*, *AAVS1*, *BCL11A-1*, and *BCL11A-2*, respectively (Fig. 3b). The total editing efficiencies increased by 7–70% but not significant with AAV HDR donors (Fig. 3b). However, AAV HDR donors’ presence led to an over 90% significant decrease in the deletion indexes in edited HSPCs (Fig. 3c). Of note, the deletion indexes at the *AAVS1* and *BCL11A-1* loci were below zero (4.26–4.52% (background)) (Fig. 3c and Additional file 1: Fig. S4e), suggesting that providing HPSCs with AAV-HDR donors during RNP editing can decrease large deletions to background levels.

The addition of AAV donors led to high-level HDR efficiencies in iPSCs (60%, 58%, 45%, and 54% for *EEF2*, *AAVS1*, *BCL11A-1*, and *BCL11A-2*, respectively) (Fig. 3b). AAV-mediated insertion significantly increased the total editing efficiencies in *EEF2* and *AAVS1* loci but not changes in two *BCL11A* loci (Fig. 3b). At three out of the four edited loci, the deletion indexes in the RNP-AAV edited cells showed comparable levels to unedited wildtype iPSCs and RNP edited iPSCs (Additional file 1: Fig. S1d, Fig. 3c, d).

Aggregating all the above results from four editing loci, we conclude that AAV-HDR editing leads to a ~ 80% decrease in DI in T cells and HSPCs (Additional file 1: Fig. S8a, b). Similarly, we observed ~ 80% reduction in D100 in these cells (Fig. 3d, e).

### Insertion of short double-stranded oligonucleotides reduces large deletions by 60%

The HDR pathway directs site-specific transgene integration, but it is inefficient in non-dividing cells [30]. By contrast, NHEJ, the other major double-strand break repair pathway, is active in both proliferating and post-mitotic cells [2] and is generally more efficient than HDR in mammalian cells [31]. Thus, CRISPR-Cas9 introduced DNA cleavage followed by NHEJ repair has been exploited to generate loss-of-function alleles in protein-coding genes [32].

**Fig. 4:**
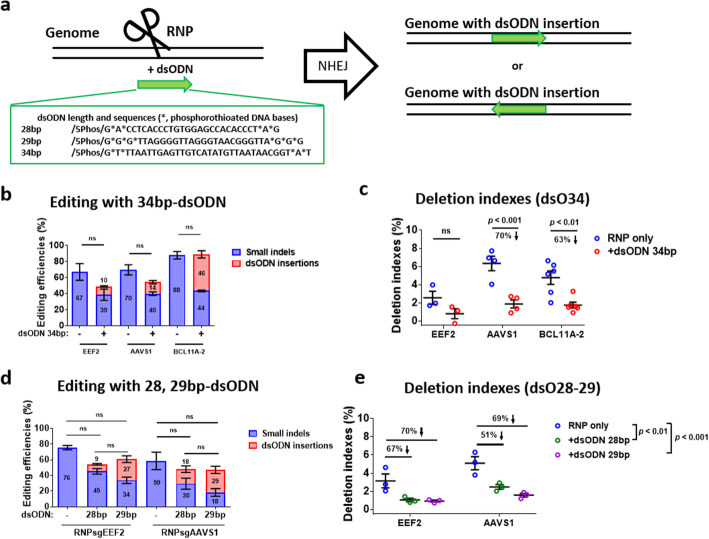
Insertion of double-stranded oligonucleotides reduces large deletions in T cells. **a** Schematic of dsODN insertion into DSBs with Cas9-gRNA RNPs and dsODN. dsODN is inserted in both forward and reverse orientations. **b** Frequencies of small indels and 34-bp dsODN insertions at three loci in T cells. The numbers in the bars indicate the mean small indels or dsODN insertion efficiencies. Small indels (NHEJ) and dsODN insertions (treated as HDR by CRISPResso2) were determined by amplicon sequencing and CRISPResso2 analysis. **c, e** Insertions with 28, 29, or 34 bp-dsODN decrease deletion indexes in T cells. Deletion indexes were assessed by long PCR, nanopore sequencing, Minimap2 alignment, and Samtools analysis. **d** Frequencies of small indels and dsODN insertions after editing with RNP and 28- or 29-bp dsODN. Error bars represent the mean ± SEM of *n* = 3–6 independent experiments. The data in **b–e** were statistically analyzed by a two-way ANOVA test. Adjusted *p* values were indicated. “ns” means no significance (*p* > 0.05)

The above studies demonstrated that the provision of HDR donors decreased large deletions by 80% in T cells and HSPCs. However, this strategy may not be practical in gene knockout applications without HDR donors. SpCas9 usually leaves two blunt ends after cleavage, leading to the perfect religation of two DSB ends by NHEJ. In this scenario, a secondary cut may occur, which might increase the frequency of large deletions. Due to the integration of a blunt double-stranded oligodeoxynucleotide (dsODN) at DSBs via NHEJ [3], we hypothesized that timely insertion of a short fragment at DSB ends would decrease deletion occurrences. For that purpose, we included chemically modified 34 bp dsODN (Fig. 4a) during the RNP nucleofection of human T cells, HSPCs, and iPSCs. We observed efficient dsODN insertion into DSBs (Additional file 1: Fig. S6a). The dsODN insertion rates depended on targeting sites and cell types, ranging from 4.4 to 52% in this study. The average dsODN insertions at *EEF2* and *AAVS1* were 10% and 14%, respectively (Fig. 4b). We observed a 22–27% but not significant decrease in total editings after 34 bp dsODN nucleofection in T cells, likely due to cytotoxicity of relatively long dsODN (Fig. 4b). Even so, we found that dsODN insertion decreased the deletion index by ~ 70% (Fig. 4c and Additional file 1: Fig. S6g). At the *BCL11A-2* target site, we observed a relatively high insertion rate (46%) and no change in total editing efficiencies (Fig. 4b). Similarly, the deletion levels were reduced by ~ 63% (Fig. 4c and Additional file 1: Fig. S6g). The dsODN insertion was NHEJ-dependent, as inhibition of the NHEJ pathway with M3814 significantly decreased its insertion by over 90% (Additional file 1: Fig. S6b). We also examined the dsODN dosage effects and observed reduced insertion efficiencies with a reduced dsODN amount (Additional file 1: Fig. S7a-c). Although dsODN insertion efficiencies were different at different loci, these results established a causal relationship (*R*^2^ = 0.51~0.76) between the dsODN insertion and attenuated deletion rates (Additional file 1: Fig. S7d).

In HSPCs, we observed dsODN insertion rates of 14%, 18%, 12%, and 52% at *EEF2*, *AAVS1*, *BCL11A-1*, and *BCL11A-2* targeting sites, respectively (Additional file 1: Fig. S6c). In the presence of the 34 bp dsODN, the total editings showed no significant changes (Additional file 1: Fig. S6c). Similar to T cell editings, dsODN insertion in HSPCs led to a reduction of 59%, 64%, 66%, and 27% in deletion indexes at the four loci (Additional file 1: Fig. S6d, g). In iPSCs, we observed less pronounced effects of dsODN insertion on reducing deletions, which may attribute to low-level deletion indexes that masked small changes (Additional file 1: Fig. S6e-g).

To decrease the cytotoxicity of the 34 bp dsODN, we used a 28 bp and a 29 bp dsODN for further studies (Fig. 4a). The 28 bp dsODN were inserted in 9–18% of T cells with RNPs targeting *EEF2* and *AAVS1* (Fig. 4d), leading to a 51–67% reduction in deletions (Fig. 4e). In comparison, the 29 bp dsODN were inserted in 27–29% of T cells at the *EEF2* and *AAVS1* sites (Fig. 4d), leading to a 70% reduction in deletions (Fig. 4e). Together, these data demonstrate that insertion of a short (28–34 bp) dsODN into DSBs by NHEJ leads to ~ 60% reduced large-fragment deletion mutations in human T cells and HSPCs (Additional file 1: Fig. S8a, b).

### Inhibition of NHEJ leads to increased large fragment deletions

**Fig. 5:**
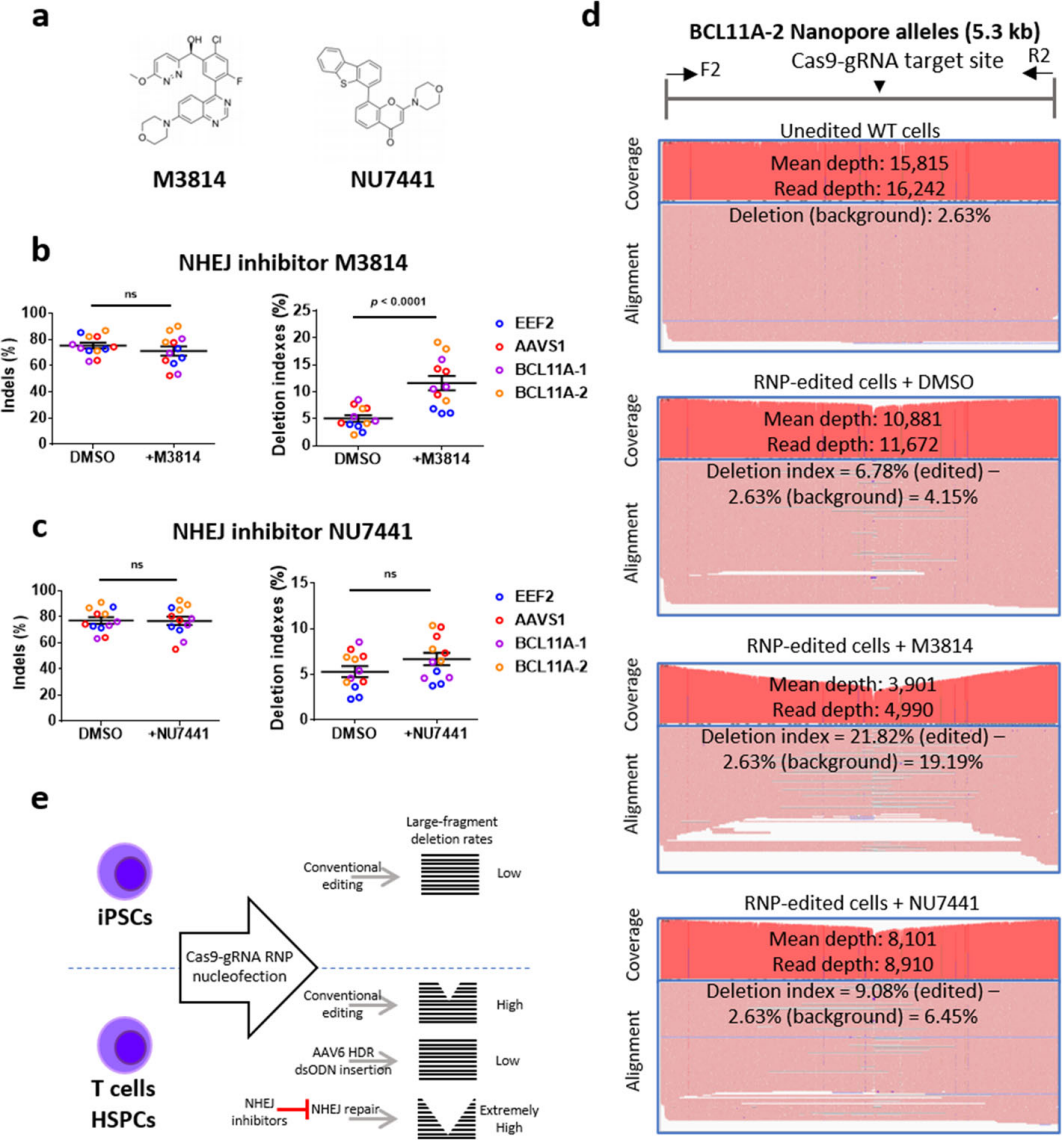
Inhibition of NHEJ leads to increased large fragment deletions. **a** Molecular structures of NHEJ inhibitors (M3814 and NU7441). **b**, **c** M3814 (**b**) or NU7441 (**c**) does not affect indel frequencies but significantly increases deletion indexes. **d** Visualization of changes in large deletions after inhibition of NHEJ or MMEJ repair pathways. Long PCR amplicons were aligned with references using Minimap2 and visualized with IGV. Deletions were calculated by (Read depth − Mean depth)/(Read depth). Unedited wildtype cells showed a background deletion. This noise was subtracted from the deletion of edited cells to obtain deletion indexes. **e** Schematic summary for the impact of DNA repair pathways on large deletions after CRISPR-Cas9 editing. The data in **b** and **c** were statistically analyzed by a two-way ANOVA test. Adjusted *p* values were indicated. “ns” means no significance (*p* > 0.05)

HDR, NHEJ, and microhomology-mediated end-joining (MMEJ) are the three prevailing cellular pathways for repairing dsDNA break s[33]. We have shown that timely repair of dsDNA damage with an HDR donor considerably curtailing extensive DNA injuries. To further assess the NHEJ pathway’s role, we used NHEJ inhibitors (M3814 and NU7441) (Fig. 5a) during T cell editing. These NHEJ inhibitors had no apparent effects on editing efficiencies (Fig. 5b, c), yet M3814, but not NU7441, significantly increased the deletion indexes after gene editing in four individual targeting sites (Fig. 5b–d). The more pronounced effect of M3814 than NU7441 on promoting disruptions correlated with its more significant inhibition on the NHEJ pathway (Additional file 1: Fig. S9), consolidating the impact of NHEJ on protecting DNA end damage. To further confirm the role of inhibitors, we analyzed the predominant +A type NHEJ and − 25 bp type MMEJ (with micro-homology CAGGAAG) frequencies after RNP editing at the *EEF2* locus (Additional file 1: Fig. S9). We found a significant decrease in the +A NHEJ frequencies and a significant increase in the -25 bp MMEJ frequencies with either M3814 or NU7441 treatment (Additional file 1: Fig. S9). Together with HDR editing results, these data demonstrate that the HDR and NHEJ pathways play a dominant role in preventing large deletions (Fig. 5e).

### Single-cell cloning analysis consolidates the editing results of bulk iPSC cells

We then analyzed large deletions at a single-cell level to assess the potential bias introduced by PCR and nanopore sequencing of the bulk edited cells. We used a sgRNA to target *BCL11A-1* locus with or without AAV6, a ssODN donor, or dsODN, followed by single-cell cloning in 96-well plates by FACS. A total of 52 single-cell clones of edited iPSCs was grown out and thus available for nanopore sequencing and qPCR analysis.

**Fig. 6:**
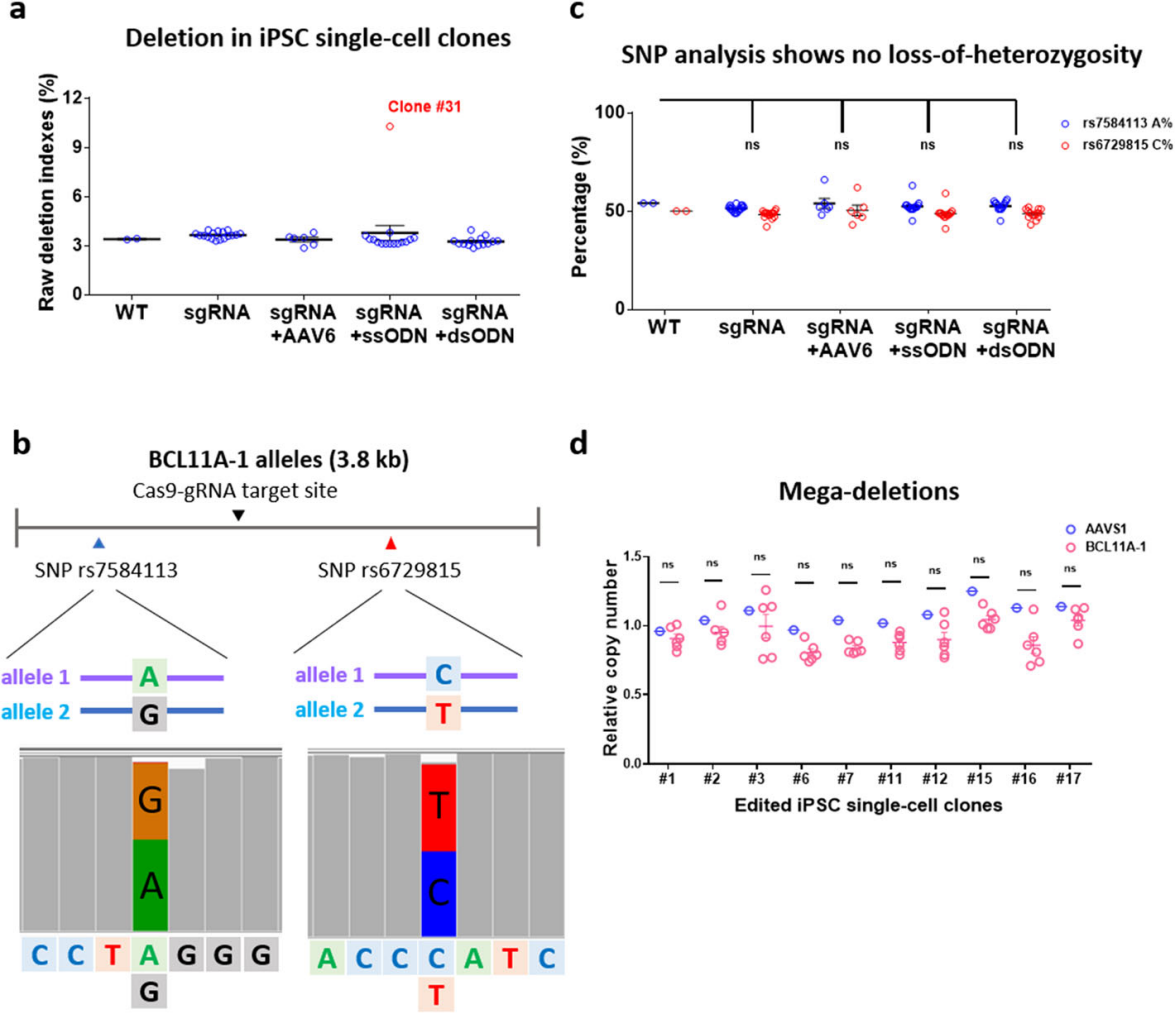
Analysis of large deletions in iPSC single-cell clones. **a** Raw deletion indexes of iPSC single-cell clones. Clone #31 highlighted in red indicated the only one clone with significant deletion mutation on one allele among 52 gene-edited iPSC single-cell clones. See details of all the clones in Additional file 1: Fig. S10. **b** Heterozygous genotype of the two SNPs identified on the *BCL11A* alleles. **c** No loss of heterozygosity in all the iPSC single-cell clones by SNP analysis. A total of 52 edited single-cell clones were analyzed by PCR and nanopore sequencing. **d** Assessment of mega-deletions in edited iPSC single-cell clones by qPCR analysis. We conducted qPCR to assess copies of gDNA at 40, 80, and 160 kb away from the target site *BCL11A-1*. *AAVS1*, located on another chromosome, served as a control. The data from WT cells were used to normalize the copy numbers of edited cells. We aggregated all the data points surrounding the *BCL11A-1* editing site of each clone to increase statistical power. The data in **c** and **d** were statistically analyzed by a two-way ANOVA test. “ns” means no significance (*P* > 0.05).

Similar to our bulk cell results, we did find 51 sgRNA targeted clones that showed a background level of deletion indexes and D100 (Fig. 6a and Additional file 1: Fig. S10). We found only one clone with a 482-bp deletion in one allele (Additional file 1: Fig. S10). A recent study showed up to 40% loss-of-heterozygosity in gene-edited iPSCs [34]; thus, we addressed this issue in our single-cell clones. We used two identified SNPs (rs7584113 and rs6729815) on the *BCL11A* allele around the target site to identify potential loss-of-heterozygosity (Fig. 6b). We did not find any loss-of-heterozygosity in all of our edited clones (Fig. 6c).

Previous studies have reported mega-deletions after editing [34], whereas long PCR cannot detect this type of mutagenesis. Therefore, we conducted qPCR analysis to assess relative copies of gDNA at 40, 80, and 160 kb away from the target site compared to unedited WT iPSCs. This analysis did not identify any appreciable changes in copy numbers in edited iPSC single-cell clones (Fig. 6d). The above data corroborate the conclusion that RNP-edited iPSCs carry considerably lower deletions relative to T cells and HSPCs.

One concern in long PCR of mixed editing events is preferential amplification of short alleles, which would lead to artificially high-level long-range deletions. For example, in clone #31, we found that a 482-bp deletion allele constituted 62% reads of the relevant single-cell clones, which is higher than the expected 50% (Additional file 1: Fig. S10). These data support the notion that current PCR technologies preferentially amplify sequences with a shorter length. Accordingly, the proportion of alleles with long deletions might have been overestimated, casting doubt on the reliability of our conclusions. However, two facts argue against this concern: (1) the bias is systematic and thus does not affect the conclusion of well-controlled studies; (2) D1000, particularly D2000, makes up less than 10% of significant deletions (Additional file 1: Fig. S2).

## Discussion

The CRISPR-Cas9 genome editing technology has been transforming the landscape of cell therapy and gene therapy. The avenue to clinical treatment has been hampered by the earlier discovery of off-target effects, and more recently, by large deletions. New developments, such as high-fidelity nuclease and truncated and modified gRNA, effectively controlled the off-target mutations. The large deletion mutations have evaded detection since the Illumina Next Generation Sequencing (NGS) technology only reveals deletions of up to 100 bp. The ability to amplify long PCR products and sequence long DNA molecules led to identifying omissions of kilobases. The large fragment deletion occurred after CRISPR-Cas9 induced gene editing in mouse embryonic stem cells and human embryos [10, 11]. Extending previous reports, we find the same phenomenon in RNP-edited human primary T cells, HSPCs, and iPSCs.

To ensure a smooth transition of CRISPR-Cas9 based editing to clinical therapy, we have endeavored to develop tools for effective control of large mutations. Using the affordable nanopore long-read sequencing technology, we comprehensively investigated how to utilize the cell-intrinsic DNA damage repair pathways to attenuate large deletions. For the first time, we show that timely repair after dsDNA cleavage by ssODN-based HDR, AAV6-based HDR, or dsODN insertion by NHEJ can effectively control this type of genome damage. We focused our studies on cell types of great translational interest; however, most of our discovery should also extend to other cell types.

Even though we observed similar editing efficiencies with high-performance guide RNAs, some sites showed more deletions than others, suggesting that differential DNA damage may be contextual, depending on chromatin structure and nucleotide components. We studied clinically relevant human T cells and stem cells (including HSPCs and iPSCs), all with intact DNA damage repair processes. Of interest, T cells showed more large deletions after RNP transfection than other cell types. Given that edited Chimeric Antigen Receptor (CAR)-T cells (CAR-T) are entering the clinic, this is a concern that deserves attention. To a less extent, we also observed large deletions in RNP-edited human cord blood HSPCs.

In contrast, human iPSCs in this study showed low-level large deletions and no appreciable mega-deletions surrounding the Cas9-sgRNA cut site (Fig. 6, Additional file 1: Fig. S2 and Fig. S10). This discrepancy might attribute to the different approaches for delivering editing components and data analysis methods. We conducted long-range PCR and nanopore sequencing to assess large deletions. Although we showed no apparent large deletions in gene-edited iPSCs compared with wildtype cells, we still found one clone with large deletion among the 52 iPSC single-cell clones. Thus, gene-edited iPSCs may need rigorous analysis of on-targets to avoid unintended outcomes. It is well-known that plasmid-mediated high-level expression of CRISPR increases off-target effects by over 10-fold relative to RNP delivery [35]. Even so, RNP still led to salient disruptions in HSPCs and T cells. We reason that two factors might have contributed to the protection of iPSCs from significant damages: (1) In response to DSBs, iPSCs are prone to p53-dependent cell death and cell cycle arrest [36], leading to selection against disrupted cells; (2) After RNP delivery, ~ 50% iPSCs were in the G2/M cell cycle, whereas only ~ 20% HSPCs or T cells were in the same phase. Cells in the G2/M phase are proficient in HDR [27], leaving less chance for erroneous DNA repair.

Due to cell types and gene loci’s heterogeneity, the extent of large mutations needs to be assessed individually. We envision using one of the ssODN-HDR, AAV-HDR, and dsODN-NHEJ insertion approach to effectively curtail immense DNA damage in most scenarios. Besides that, adopting more than one method will considerably decrease large deletions by ~ 90% at editing sites with a high tendency for large deletions.

Transfection of RNP to create loss-of-function has been used in clinical trials, such as CAR-T therapy [37]. However, large deletions and translocations have been reported in these edited cells [10]. Our data suggest that the inclusion of an HDR donor and a dsODN would considerably decrease this adverse effect, with an extra benefit of enhanced gene depletion efficiency. This study used a 29 bp dsODN with three stop codons. We speculate that this dsODN insertion in the protein-coding region may lead to high-level gene knockout. However, the dsODN poses a risk to random insertion at dsDNA breaks induced by replication or other stresses [3]. Further investigations will gain more insight into this potential concern.

Following DSBs after CRISPR-Cas9 cleavage, DNA repair machinery is activated and recruited to promote end ligations through several damage repair pathways. These include NHEJ, alternative end-joining or microhomology-mediated end joining (alt-EJ/MMEJ), and HDR in the presence of a donor template flanked with homology arms [33]. This process results in random repair outcomes in the absence of a donor template, leading to small indels at high frequencies and large deletions at lower frequencies. Our recent study examined the editing dynamics and patterns of CRISPR-Cas9 editing [22], revealing that short indels (such as +A or +T type NHEJ) occur faster than longer deletions (> 2 bp), and the AAV6-mediated HDR occurs faster than MMEJ but slower than NHEJ. As such, we hypothesize that the timely repair of DSBs decreases the events of large deletions, which is primarily a consequence of MMEJ repair. Our data in this study also shows that the inhibition of NHEJ by small molecules M3814 or NU7441 disrupts the NHEJ dependent repair. Therefore, NHEJ inhibition leads to more MMEJ-mediated deletions (Additional file 1: Fig. S9) and significant disruptions. On the other hand, in the presence of an HDR template, the damaged ends would have a greater chance to be fixed by homologous recombination promptly, thereby reducing the possibilities of large deletions. Cas9-gRNA predominantly creates blunt ends [38], which can be correctly linked by NHEJ. However, the reemergence of the Cas9-gRNA target site leads to a secondary cut. Our unique finding was that providing a short dsODN leads to its insertion at the DSBs, preventing further damage. Together, timely repair of DSBs decreases large deletions, whereas delayed restoration heightens the possibility of severe damage to the genome.

Our studies are limited to examining 4–6 kb deletions due to long-range PCR technical limitations. Most omissions occur in the proximity of the Cas9-sgRNA target sites. Mega deletions, if any, are expected to be rare events. A previous study showed the occurrence of loss-of-heterozygosity after editing [34], but our analysis of iPSC single-cell clones did not identify any loss-of-heterozygosity. Besides, due to off-target cuts and other damages induced by cell replicative stress or environmental insults, cells may simultaneously possess multiple dsDNA breaks. Such a situation will increase the occurrences of rearrangements, insertions, and translocations. Rearrangements and insertions are a somewhat frequent phenomenon after editing that can affect the edited locus and chromosome integrity, which need to be addressed in future investigations. It is not our intention to investigate the impact of our proposed strategies on translocations. Still, we speculate that our approach should also considerably decrease mega deletions and translocations due to the timely bridging of broken DNA ends. Human T cells and HSPCs have become the widely used cell sources in clinical cell-based gene therapies recently [39, 40]. We envision that the rational adoption of our strategies in clinical gene editing protocols will safeguard successful clinical treatments.

## Conclusions

In summary, empowered by long-range PCR and nanopore sequencing technologies, we discover frequent larger deletions after CRISPR-Cas9 mediated genomic editing in human primary T cells and HSPCs, and considerably low-level yet noticeable disruptions in iPSCs. The differences of large deletions may be attributable to differences in cell cycle profiles following RNP delivery. The use of AAV6 as the HDR donor can reduce the large deletions by 80%, and the dsODN insertion in DSB reduces the large deletions by 60%. These findings will stimulate the endeavor to develop safer gene-editing strategies.

## Methods

### Cell culture

Peripheral blood mononuclear cells (PBMCs) were isolated from healthy donors’ peripheral blood (PB) by density gradient centrifugation with Ficoll-Hypaque (1.077 g/mL). T cells were purified from PBMCs with CD3 magnetic beads. Primary human T cells were cultured in serum-free ImmunoCult™-XF T Cell Expansion Medium (Stemcell Technologies) supplemented with 10 ng ml^−1^ recombinant human interleukin (IL)-2 (Peprotech). We cultured T cells in non-tissue culture-treated 6-well plates with 20 μl ml^−1^ Dynabeads Human T-Activator CD3/CD28 (Gibco).

Cord blood CD34^+^ HSPCs were purified with CD34 MicroBead Kit (Miltenyi Biotec). The enriched HSPCs contained over 90% of CD34^+^ cells. HSPCs were seeded at 5 × 10^5^ cells per mL in serum-free StemSpan™ SFEM II medium (Stemcell Technologies) supplemented with 1% glutamine, 100 ng ml^−1^ recombinant hSCF (Peprotech), 100 ng ml^−1^ recombinant hFlt3-L (Peprotech), 100 ng ml^−1^ recombinant hTPO (Peprotech), 50 ng ml^−1^ recombinant hIL-6 (Peprotech), 750 nM SR1 (Sigma), and 50 nM UM171 (Sigma).

iPSCs were generated by PBMC reprogramming as previously described [41, 42]. iPSCs were maintained on Matrigel (BD) coated 6-well plates and cultured in StemFlex™ Medium (Gibco). The 10 μM ROCK inhibitor Y-27632 (STEMGENT) was added to the medium during the first day after passaging with Accutase (Stemcell Technologies). All cells were cultured in a 5% CO_2_ humidified atmosphere at 37 °C.

### Cas9-gRNAs and RNP formation

We used CHOPCHOP [43] to design high-performance gRNAs targeting *EEF2*, *AAVS1,* and *BCL11A* (targeting two individual sites). Additional file 1: Table S1 listed the gRNAs used in this study. The modified synthetic crRNAs and tracrRNA were purchased from Synthego or Integrated DNA Technologies (IDT), which showed indistinguishable efficacies. To prepare the gRNA complex, we combined 12 μl crRNAs (200 μM), 6 μl tracrRNAs (200 μM), 8 μl 5 × annealing buffer (Synthego), and 14 μl nuclease-free water. After heating the mixture at 78 °C for 15 min, they were cooled to room temperature. To prepare RNPs, we mixed Cas9 protein and gRNA (molar ratio 1: 2.5) at room temperature for 10–20 min before mixed with nucleofection buffers.

### ssODN HDR donors

We designed ssODN HDR donors of 50 bases at both left and right homology arms with a short insertion. The ssODN donors were phosphorothioate-modified to enhance cellular stability and synthesized by IDT [16]. 40 pmol ssODN was used in each transfection—Additional file 1: Table S2 listed the ssODN sequences used in this study.

### AAV HDR donors

We used AAV donors to guide HDR editing after Cas9-gRNA mediated dsDNA cutting. The AAV HDR vector consisted of a backbone with AAV2 inverted terminal repeat (ITR) of 145 bases, a short insert of 8–15 bp (for analysis by Illumina sequencing), or a fluorescent protein (for detection of HDR efficiency by FACS) flanked by 600 bp homologous arms. All the fragments were amplified from human genomic DNA or plasmids in our lab by PCR using KAPA HiFi polymerase (KAPA Biosystems) and purified using the GeneJET Gel Extraction Kit (Thermo Fisher Scientific). The PCR products were assembled using the NEBuilder HiFi DNA Assembly kit. Multiple colonies were chosen for Sanger sequencing (MCLAB) to identify the correct clones. Supplementary Additional file 1: Fig. S11 listed AAV HDR donor sequences used in this study.

### AAV6 packaging, purification, and titering

We used PEI (polyethylenimine) MAX 40 K (Polysciences) to produce recombinant AAV vectors as detailed previously [44]. In brief, HEK293T (ATCC) cells were transfected with the AAV6 capsid plasmid (Cell Biolabs), AAV helper plasmid (Cell Biolabs), and AAV HDR vector. Five days after transfection, the supernatant was harvested after treating with 500 mM NaCl (Sigma) and 20 U/ml Benonase (SCBT). The virus-containing supernatant was concentrated 20-fold using Minimate (PALL) tangential flow filtration system equipped with a 300K molecular weight cutoff (MWCO) capsule. The AAV6 vectors were further purified by iodixanol gradient centrifugation. Finally, AAV6 vectors were titrated by qPCR analysis, as detailed previously [44].

### Gene editing of human T cells.

After 4 d of stimulation, ~ 95% of cells were CD3^+^. 1.0–1.5 × 10^6^ cells were used for each transfection. Cells were electroporated with RNP of Cas9-gRNA at a final concentration of 3.1 μM using P3 Primary Cell 4D-Nucleofector X Kit (Lonza, V4XP-3032) and program EH-115. Where specified, a ssODN donor or annealed dsODN was added to the electroporation mixture at a final concentration of 1.9 μM or 2.4 μM, respectively. After incubating the cuvette at 37 °C for 5 min, we seeded the cells in 24-well plates at a density of 5 × 10^5^ cells per mL. For AAV6-based HDR gene editing, 1 × 10^4^ vector genome copies (vg) per cell of AAV6 was added in the culture within 15 min after electroporation.

### Gene editing of human HSPCs.

After 2 days of stimulation, 0.5–1.0 × 10^6^ cells were used for each transfection. Cells were electroporated with RNP at a final concentration of 3.1 μM using P3 Primary Cell 4D-Nucleofector X Kit (Lonza, V4XP-3032) and program DO-100. Where specified, a ssODN donor or dsODN was added to the electroporation mixture at a final concentration of 1.9 μM or 2.4 μM, respectively. After electroporation, the cuvette was incubated at 37 °C for 5 min. The cells were then seeded at a density of 5 × 10^5^ cells per mL in 24-well plates. For AAV6-mediated HDR editing, cells were cocultured with 1 × 10^4^ vg per cell of AAV6.

### Gene editing of human iPSCs.

Human iPSCs at 60–70% confluency were disassociated with Accutase to obtain a single-cell suspension. 1.0–1.5 × 10^6^ cells were washed with five volumes of DPBS (Gibco) and harvested by centrifugation at 200×*g* for 5 min. iPSCs were resuspended in a 70-μl Stem Cell Nucleofector® Kit 2 (Lonza) electroporation solution with RNP at a final concentration of 0.8 μM together with 0.5 μg of a BCL-XL expressing plasmid [18]. We used Nucleofector™ 2b and program B-016 for the transfection of iPSCs. Where specified, a ssODN donor or dsODN was added to the electroporation mixture at the final concentrations of 0.5 μM or 0.6 μM, respectively. After electroporation, the cuvette was incubated at 37 °C for 5 min. The cells were then seeded at a density of 0.5–1.5 × 10^6^ cells in each well of Matrigel-precoated 6-well plates. The 10 μM ROCK inhibitor Y-27632 was added to the medium. For AAV6-based gene editing, cells were transduced with 1 × 10^4^ vg per cell of AAV6. Twenty-four hours later, AAV was removed, and the culture was refreshed with iPSC medium without the ROCK inhibitor.

### Long-range PCR

Cells were harvested 3 days after the transfection of gene-editing components for genomic DNA extraction using the Gentra Puregene Blood Kit (Qiagen). The *EEF2*, *AAVS1*, *BCL11A-1*, and *BCL11A-2* target sequences were amplified with PrimeSTAR® GXL Premixed DNA polymerase (Takara Bio). The PCR cycling condition was 98 °C for 10 s, 60 °C for 15 s, and 68 °C for 1 min per kb for 30 cycles. The 8–12 nt barcodes were added in 5' of the forward primers—Additional file 1: Tables 3-6 listed long-range PCR primers used in this study. An equal amount of PCR products with different barcodes were mixed for nanopore sequencing.

### Nanopore sequencing

A total amount of 8 μg DNA per sample was used as input material for library preparation. The SQK-LSK109 Kit (ONT, UK) was used to construct a 1D library, which means that the sense chain and antisense chain in the library are entirely separated and sequenced separately in the sequencing process. The DNA library was created by a standard ligation method without DNA fragmentation and depleting small fragments. After end-repair and A-tailing, the sequencing adaptor, motor protein, and tether protein were connected to prepare the DNA library. The library was sequenced using PromethION (ONT, UK) at Novogene (Tianjin, China). Albacore (version 2.3.1, Oxford Nanopore Technologies) was used to transform raw fast5 data into bases and quality scores.

### Nanopore sequencing data analyses

We first removed sequencing adapters using Porechop [45] (version 0.2.4) with the “--extra_end_trim 0” option and then processed with Seqkit to grep for individual reads of barcoded PCR products, as illustrated in Additional file 1: Fig. S1b. We obtained an average of over 10,000 reads for each amplicon. We used Minimap2 [24] (version 2.14) with the “-x map-ont” option to align the fastq sequences to the reference fasta files. Additional file 1 listed the reference amplicon sequences of *EEF2*, *EEF2* with mNeonGreen insertion, *AAVS1*, *BCL11A-1*, and *BCL11A-2*. The aligned bam files were visualized by IGV [46] (version 2.8.2). To determine the deletion index, we retrieved coverage data by Samtools using the command “Samtools coverage file.bam.” The value “meandepth” was considered mean depth in the manuscript. And the raw deletion indexes (including the background) were calculated by [100 – (mean depth × 100)/total reads] %.

### Illumina amplicon sequencing and editing efficiency analysis

Long-range PCR products were used as templates for secondary PCR after 100x dilution to obtain amplicons of 200–240 bp in length for Illumina paired-end 150 bp sequencing. The secondary PCR was conducted using KAPA HiFi polymerase, with cycling conditions as follows: 98 °C for 1 min, followed by 98 °C for 5 s, 64 °C for 10 s, and 72 °C for 15 s for 20 cycles. The barcoded primers were used as previously described [17, 44]. The PCR primers used in this study were listed in Additional file 1: Tables 7-10. For data analysis, the paired-end fastq data were merged with FLASH [47], followed by demultiplexing using Barcode-splitter (https://pypi.org/project/barcode-splitter/). The indel efficiencies, HDR frequencies, and dsODN insertion rates were analyzed with the docker version of CRISPResso2 [48].

### Detection of mega-deletions

Primers for qPCR analysis of DNA fragment located 40–160 kb from the target site (*BCL11A-1*) were designed using the Primer3Plus. These primers were listed in Additional file 1: Table S11. Genomic DNA (gDNA) of 50 ng was used in each reaction. The qPCR was carried out using KAPA SYBR® FAST qPCR reagent (Sigma-Aldrich) with a cycling condition of 98 °C for 2 min, followed by 40 cycles of 98 °C for 5 s and 60 °C for 30 s. Copy numbers were determined by ΔΔCt calculation relative to the internal *ACTB* reference and unedited WT.

### Flow cytometry

Flow cytometry was performed to determine the HDR efficiencies of mNeonGreen edited cells, as previously described [17, 18, 49]. Cells were acquired on a BD FACS Canto II flow cytometer 3 days after nucleofection. For the *EEF2* target site, the HDR mediated knockin of the promoterless mNeonGreen reporter led cells to fluoresce green. As negative controls, omitting gRNA or AAV donor, or providing a mismatched donor, showed 0% mNeonGreen positive cells (Additional file 1: Fig. S4b).

### Small molecules

NHEJ inhibitors M3814 and NU7441 were purchased from MedChemExpress. These compounds were solubilized in DMSO as 2 mM, and 1 mg per mL, respectively. To investigate the role of NHEJ and MMEJ in large deletion formation, we treated RNP-transfected T cells with DMSO control, 2 μM M3814, or 1 μg per mL NU7441. One day later, the cultures were refreshed with the T Cell Expansion Medium containing no inhibitors.

### Single-cell cloning and Long PCR

Single-cell sorting of iPSCs was conducted using a BD FACS Aria III with a 70 mm nozzle under sterile conditions. In each well of round-bottom 96-well plates precoated with MEF feeders, 100 μl of StemFlex™ Medium medium and 10 μM ROCK inhibitor Y-27632 was added. The culture was refreshed 7 days later. At 1~2 weeks after single-cell cloning, gDNA was extracted using a Magnetic Blood Genomic DNA Kit (TIANGEN DP329), per instructions. The bound DNA was eluted from the magnetic beads with 8-10 μl ddH_2_O and 1–2 μl were used for long-range PCR with a 20 μl PrimeSTAR® GXL Premix (Takara R051A) reaction. The PCR cycling condition was 98 °C for 10 s, 60 °C for 15 s, and 68 °C for 1 min per kb for 30 cycles.

### Cell cycle profiling

Cell cycle analysis was conducted by staining DNA and RNA with Hoechst 33342 and Pyronin Y [50]. One day after RNP nucleofection, T cells and HSPCs were harvested and resuspended in PBS. iPSCs were treated with Accutase to obtain a single cell suspension and resuspended in PBS. Cells were immediately fixed with cold 70% ethanol and incubated 2 h at − 20 °C. Cells were then collected by centrifuging for 3 min at 300×g. After washing twice with PBE (PBS containing 2% FBS and 2 mM EDTA), cells were stained with 2 μg/ml Hoechst 33342 and 4 μg/ml Pyronin Y in PBE for 20 min at room temperature in the dark. Without a washing step, the samples were analyzed by flow cytometry.

### Statistics and reproducibility

We used one-way ANOVA or two-way ANOVA to analyze paired/matched or unmatched data. The *P* values were calculated using GraphPad Prism 7.04. Adjusted *p* values were indicated. “ns” means no significance (*p* > 0.05). The statistical methods used for each experiment were detailed in the Figure legends. All the data presented were from at least three independent experiments. For T cells and cord blood-derived HSPCs, at least two different donors were used in each experiment.

## Supplementary Information

**Additional file 1.** Supplemental figures, tables and texts. The additional file 1 includes data and informations related to this manuscript but not mentioned in the main text.

**Additional file 2.** Review history.

## Data Availability

Illumina sequencing data and nanopore sequencing data are deposited in the SRA database under accession number PRJNA733835 [51]. The sample ID is SAMN19460194.
