# Supplementary material for: Effective control of large deletions after double-strand breaks by homology-directed repair and dsODN insertion
Source: Genome Biol. 2021 Aug 20;22:236. doi: 10.1186/s13059-021-02462-4 (PMC8377869; doi:10.1186/s13059-021-02462-4)
Supplement: Supplementary file 1 — Additional file 1. Supplemental figures, tables and texts. The additional file 1 includes data and informations related to this manuscript but not mentioned in the main text. [file 13059_2021_2462_MOESM1_ESM.pdf]

Fig. S1

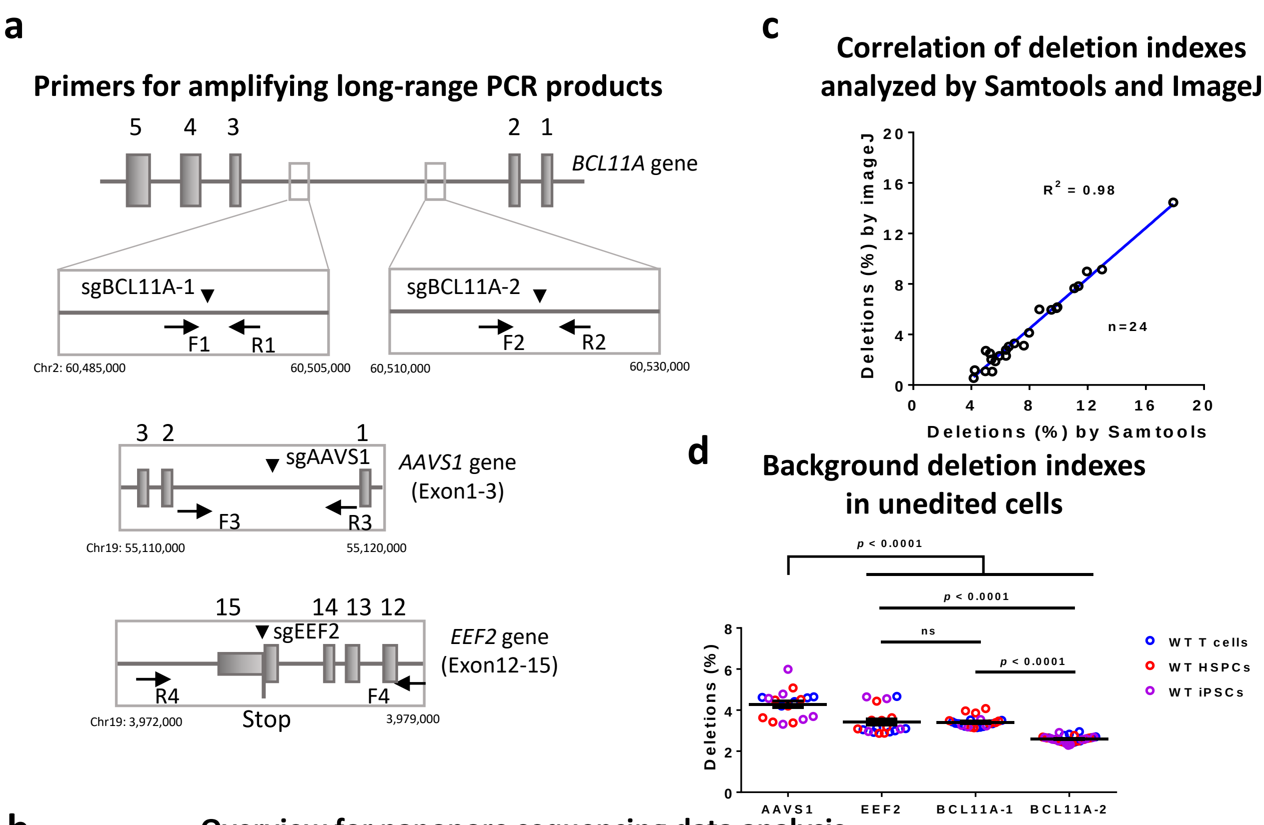

b **Overview for nanopore sequencing data analysis**

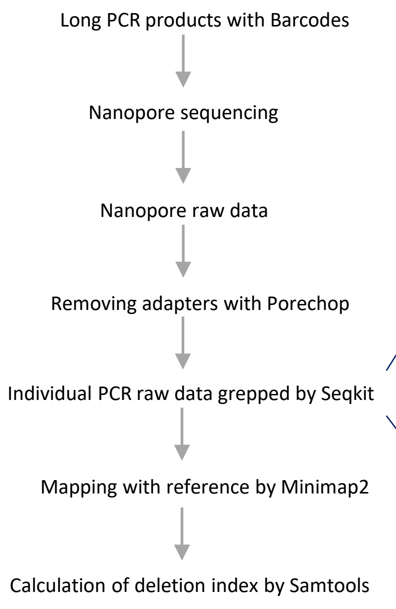

Command lines for grepping individual PCR sequences:

```
# Extract specific PCR sequences by forward barcodes
seqkit grep -s -f PCR_reads_extraction_forward_barcodes.txt
nanopore_chop.fastq >> PCR-Fwd.temp
# Reverse nanopore chop data
seqkit seq nanopore_chop.fastq -r -p -v -o nanopore _chop_reverse.fastq
# Extract specific PCR sequences from reversed chop data by forward barcodes
seqkit grep -s -f PCR_reads_extraction_forward_barcodes.txt
nanopore_chop_reverse.fastq >> PCR-Fwd.temp
# Extract specific PCR sequences from forward extracted data by reward
barcodes
seqkit grep -s -f PCR_reads_extraction_reward_barcodes.txt PCR-Fwd.temp >>
PCR.fastq
# Extract PCR reads with barcodes sequences
seqkit grep –R 1:20 -s -f Primer_barcode_file.txt PCR.fastq -o
Primer_borcode_PCR_reads.fastq
```

Fig. S2

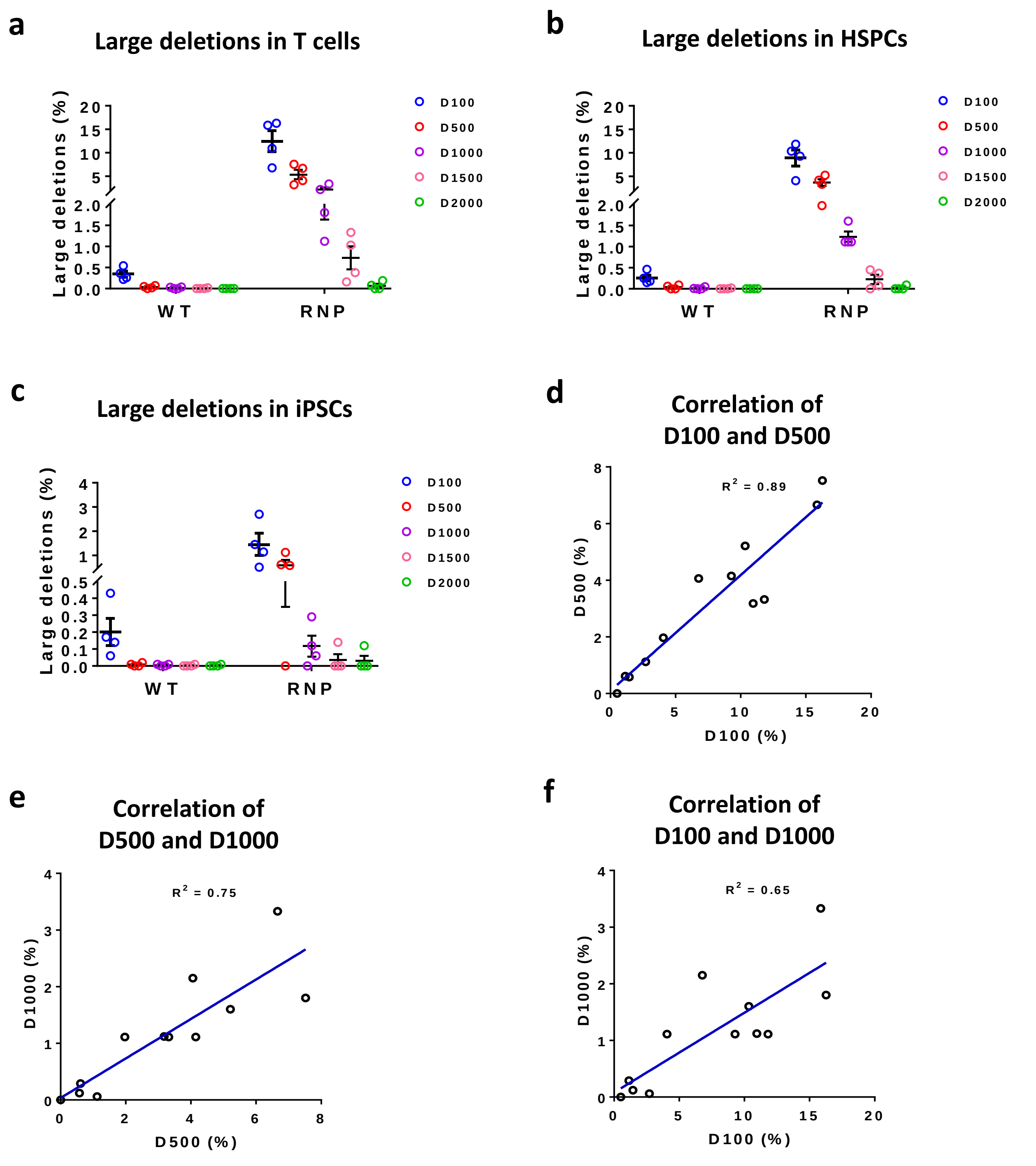
a
Large deletions in T cells
Large deletions (%)
20
15
10
5
2.0
1.5
1.0
0.5
0.0
WT
RNP
D100
D500
D1000
D1500
D2000
b
Large deletions in HSPCs
Large deletions (%)
20
15
10
5
2.0
1.5
1.0
0.5
0.0
WT
RNP
D100
D500
D1000
D1500
D2000
c
Large deletions in iPSCs
Large deletions (%)
4
3
2
1
0.5
0.4
0.3
0.2
0.1
0.0
WT
RNP
D100
D500
D1000
D1500
D2000
d
Correlation of D100 and D500
R^2 = 0.89
D500 (%)
8
6
4
2
0
D100 (%)
0
5
10
15
20
e
Correlation of D500 and D1000
R^2 = 0.75
D1000 (%)
4
3
2
1
0
D500 (%)
0
2
4
6
8
f
Correlation of D100 and D1000
R^2 = 0.65
D1000 (%)
4
3
2
1
0
D100 (%)
0
5
10
15
20

Fig. S3

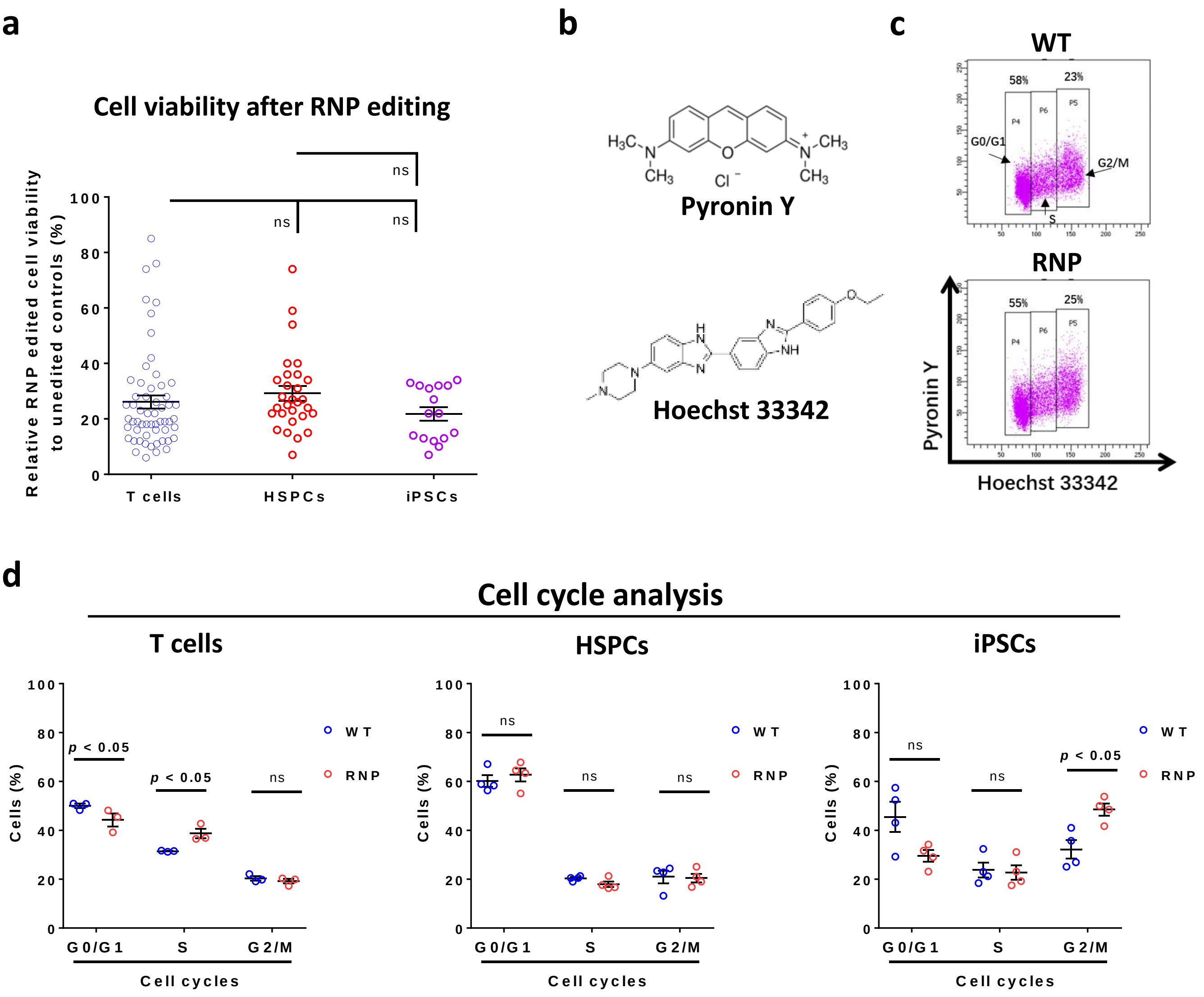

a
Cell viability after RNP editing
Relative RNP edited cell viability to unedited controls (%)
ns
ns
ns
T cells
HSPCs
iPSCs
b
Pyronin Y
Hoechst 33342
c
WT
58%
23%
G0/G1
G2/M
S
RNP
55%
25%
Pyronin Y
Hoechst 33342
d
Cell cycle analysis
T cells
HSPCs
iPSCs
Cells (%)
p < 0.05
ns
WT
RNP
G0/G1
S
G2/M
Cell cycles

Fig. S4

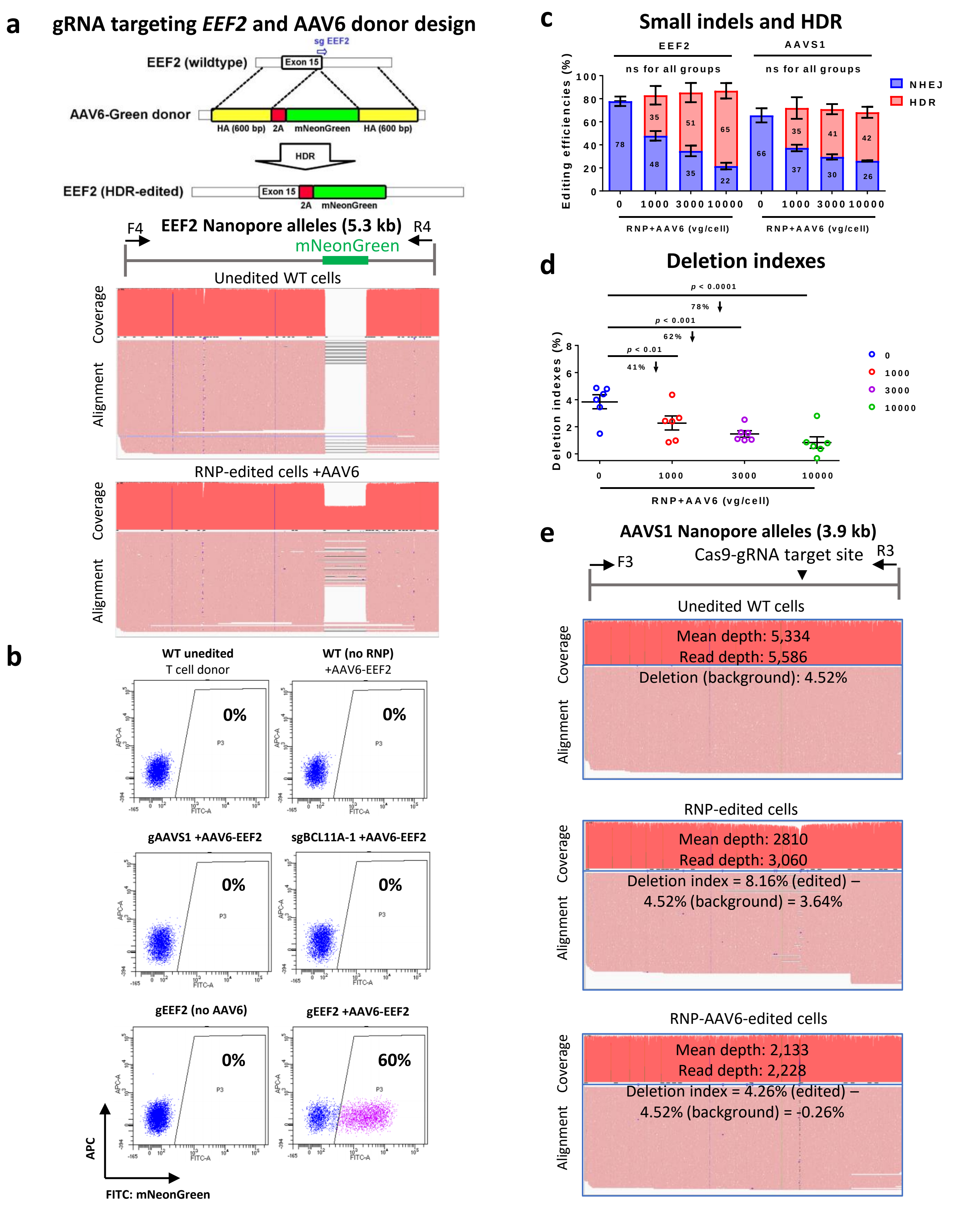
a
gRNA targeting EEF2 and AAV6 donor design
sg EEF2
EEF2 (wildtype)
Exon 15
AAV6-Green donor
HA (600 bp)
2A
mNeonGreen
HA (600 bp)
HDR
EEF2 (HDR-edited)
Exon 15
2A
mNeonGreen
F4
EEF2 Nanopore alleles (5.3 kb)
R4
mNeonGreen
Unedited WT cells
Coverage
Alignment
RNP-edited cells +AAV6
Coverage
Alignment
b
WT unedited
T cell donor
0%
WT (no RNP)
+AAV6-EEF2
0%
gAAVS1 +AAV6-EEF2
0%
sgBCL11A-1 +AAV6-EEF2
0%
gEEF2 (no AAV6)
0%
gEEF2 +AAV6-EEF2
60%
P3
APC-A
FITC-A
APC
FITC: mNeonGreen
c
Small indels and HDR
EEF2
AAVS1
ns for all groups
ns for all groups
Editing efficiencies (%)
NHEJ
HDR
78
35
48
51
35
65
22
66
35
37
41
30
42
26
0
1000
3000
10000
0
1000
3000
10000
RNP+AAV6 (vg/cell)
RNP+AAV6 (vg/cell)
d
Deletion indexes
p < 0.0001
78%
p < 0.001
62%
p < 0.01
41%
Deletion indexes (%)
0
1000
3000
10000
RNP+AAV6 (vg/cell)
e
AAVS1 Nanopore alleles (3.9 kb)
F3
Cas9-gRNA target site
R3
Unedited WT cells
Coverage
Alignment
Mean depth: 5,334
Read depth: 5,586
Deletion (background): 4.52%
RNP-edited cells
Coverage
Alignment
Mean depth: 2810
Read depth: 3,060
Deletion index = 8.16% (edited) – 4.52% (background) = 3.64%
RNP-AAV6-edited cells
Coverage
Alignment
Mean depth: 2,133
Read depth: 2,228
Deletion index = 4.26% (edited) – 4.52% (background) = -0.26%

Fig. S5

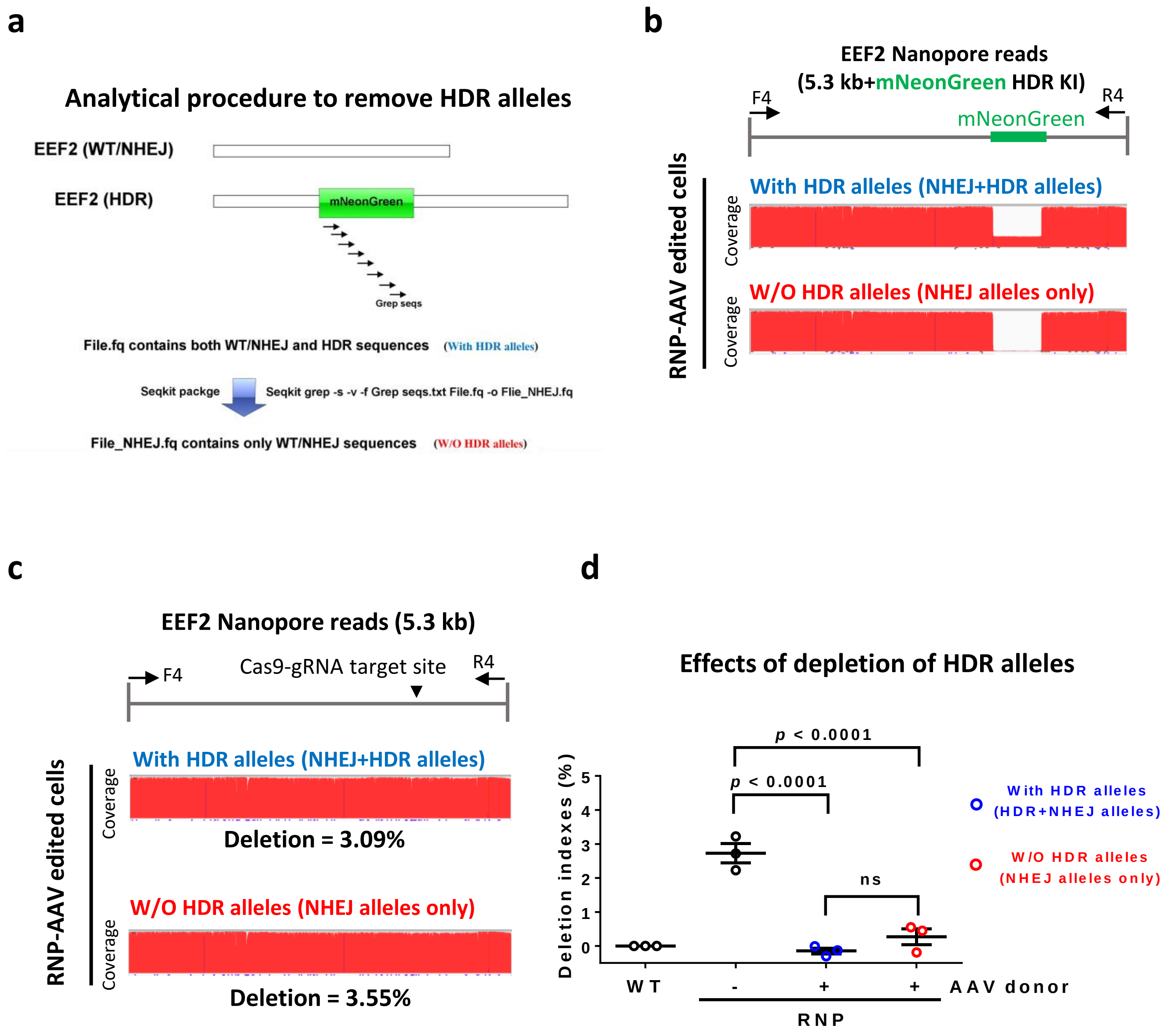
a
Analytical procedure to remove HDR alleles
EEF2 (WT/NHEJ)
EEF2 (HDR)
mNeonGreen
Grep seqs
File.fq contains both WT/NHEJ and HDR sequences (With HDR alleles)
Seqkit packge
Seqkit grep -s -v -f Grep seqs.txt File.fq -o Flie_NHEJ.fq
File_NHEJ.fq contains only WT/NHEJ sequences (W/O HDR alleles)
b
EEF2 Nanopore reads
(5.3 kb+mNeonGreen HDR KI)
F4
R4
mNeonGreen
RNP-AAV edited cells
Coverage
With HDR alleles (NHEJ+HDR alleles)
W/O HDR alleles (NHEJ alleles only)
c
EEF2 Nanopore reads (5.3 kb)
F4
Cas9-gRNA target site
R4
RNP-AAV edited cells
Coverage
With HDR alleles (NHEJ+HDR alleles)
Deletion = 3.09%
W/O HDR alleles (NHEJ alleles only)
Deletion = 3.55%
d
Effects of depletion of HDR alleles
p < 0.0001
p < 0.0001
ns
Deletion indexes (%)
5
4
3
2
1
0
WT
-
+
+
AAV donor
RNP
With HDR alleles (HDR+NHEJ alleles)
W/O HDR alleles (NHEJ alleles only)

Fig. S6

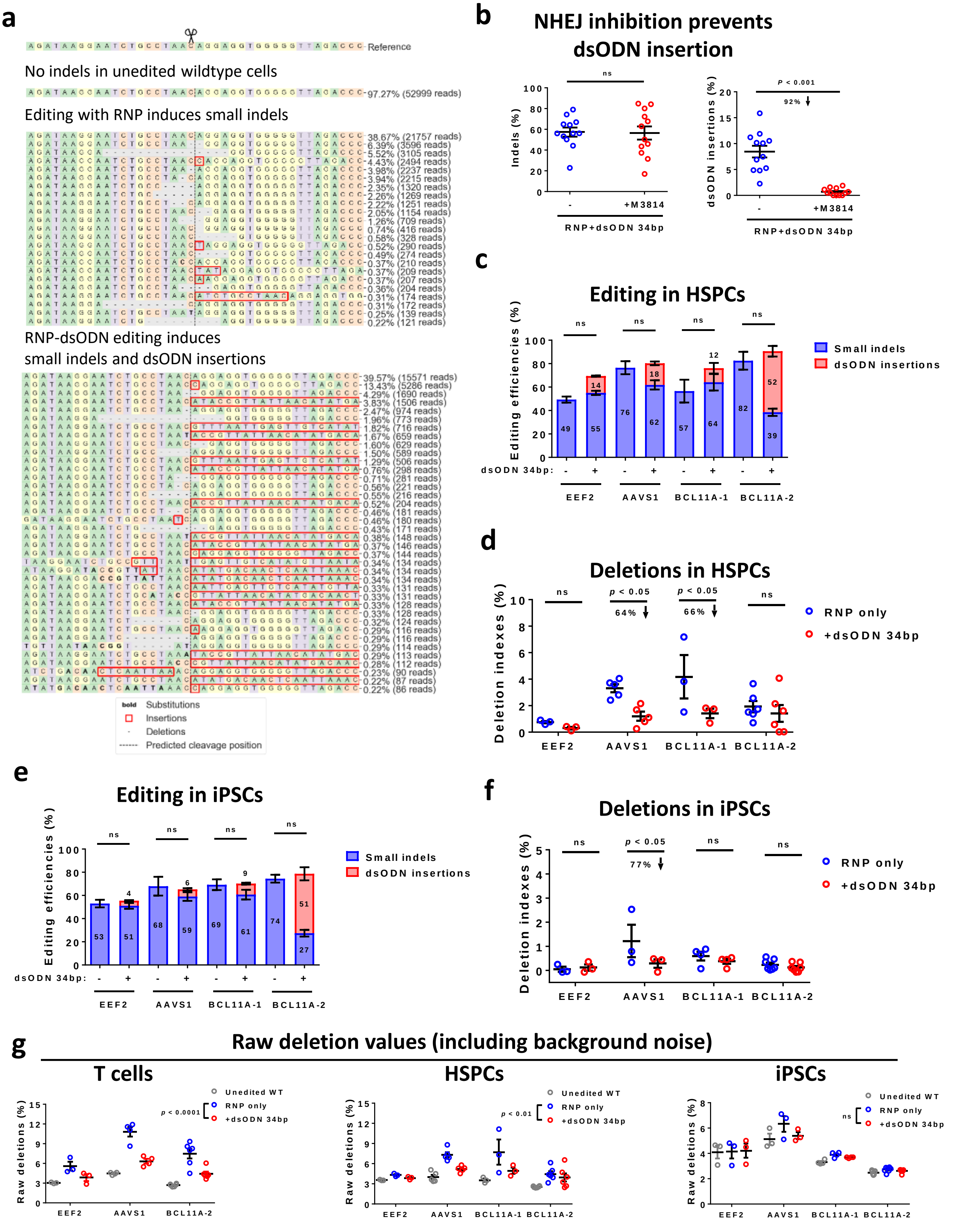

a
Reference
No indels in unedited wildtype cells
97.27% (52999 reads)
Editing with RNP induces small indels
RNP-dsODN editing induces
small indels and dsODN insertions
bold Substitutions
Insertions
Deletions
Predicted cleavage position
b
NHEJ inhibition prevents dsODN insertion
Indels (%)
dsODN insertions (%)
ns
P < 0.001
92%
+M3814
RNP+dsODN 34bp
c
Editing in HSPCs
Editing efficiencies (%)
Small indels
dsODN insertions
dsODN 34bp:
EEF2
AAVS1
BCL11A-1
BCL11A-2
d
Deletions in HSPCs
Deletion indexes (%)
p < 0.05
64%
66%
RNP only
+dsODN 34bp
e
Editing in iPSCs
f
Deletions in iPSCs
77%
g
Raw deletion values (including background noise)
T cells
HSPCs
iPSCs
Raw deletions (%)
Unedited WT
p < 0.0001
p < 0.01

Fig. S7

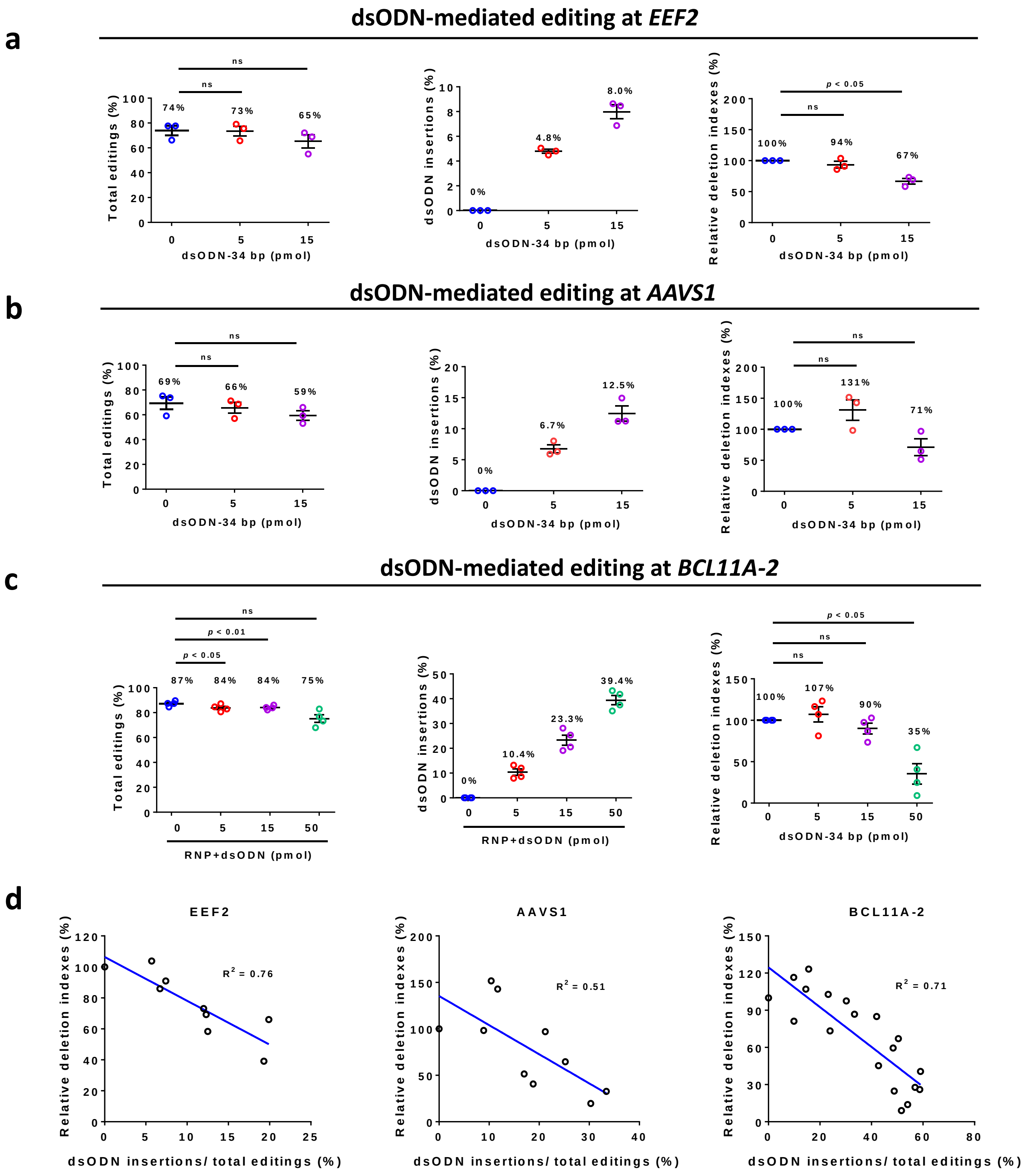
a
dsODN-mediated editing at EEF2
Total editings (%)
dsODN-34 bp (pmol)
74%
73%
65%
ns
ns
dsODN insertions (%)
0%
4.8%
8.0%
Relative deletion indexes (%)
100%
94%
67%
p < 0.05
b
dsODN-mediated editing at AAVS1
69%
66%
59%
0%
6.7%
12.5%
100%
131%
71%
c
dsODN-mediated editing at BCL11A-2
RNP+dsODN (pmol)
87%
84%
84%
75%
p < 0.05
p < 0.01
0%
10.4%
23.3%
39.4%
100%
107%
90%
35%
d
EEF2
AAVS1
BCL11A-2
R2 = 0.76
R2 = 0.51
R2 = 0.71
dsODN insertions/ total editings (%)

Fig. S8

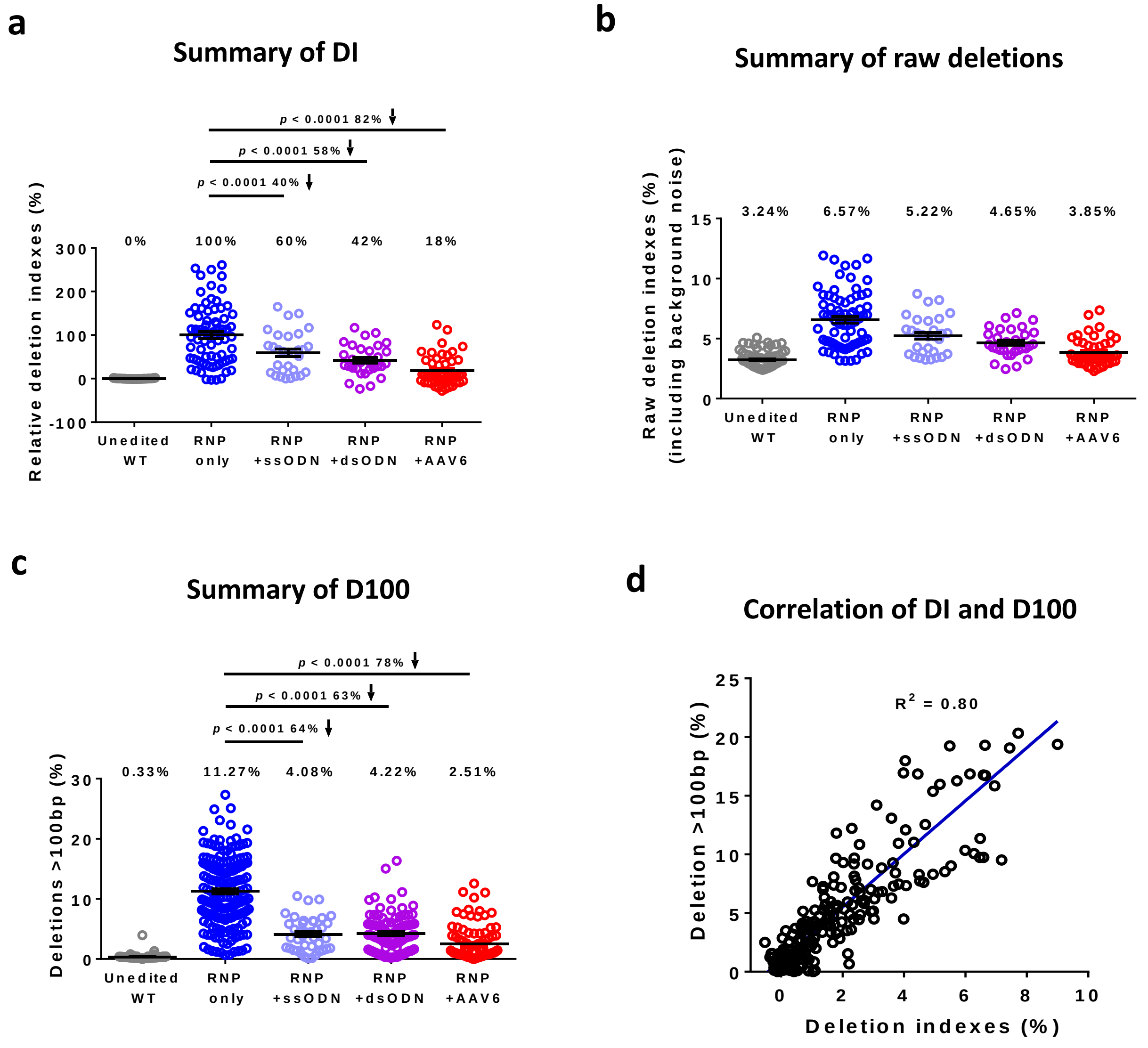
a
Summary of DI
p < 0.0001 82%
p < 0.0001 58%
p < 0.0001 40%
0%
100%
60%
42%
18%
Relative deletion indexes (%)
300
200
100
0
-100
Unedited WT
RNP only
RNP +ssODN
RNP +dsODN
RNP +AAV6
b
Summary of raw deletions
Raw deletion indexes (%)
(including background noise)
3.24%
6.57%
5.22%
4.65%
3.85%
15
10
5
0
Unedited WT
RNP only
RNP +ssODN
RNP +dsODN
RNP +AAV6
c
Summary of D100
p < 0.0001 78%
p < 0.0001 63%
p < 0.0001 64%
0.33%
11.27%
4.08%
4.22%
2.51%
Deletions >100bp (%)
30
20
10
0
Unedited WT
RNP only
RNP +ssODN
RNP +dsODN
RNP +AAV6
d
Correlation of DI and D100
R^2 = 0.80
Deletion >100bp (%)
25
20
15
10
5
0
0
2
4
6
8
10
Deletion indexes (%)

Fig. S9

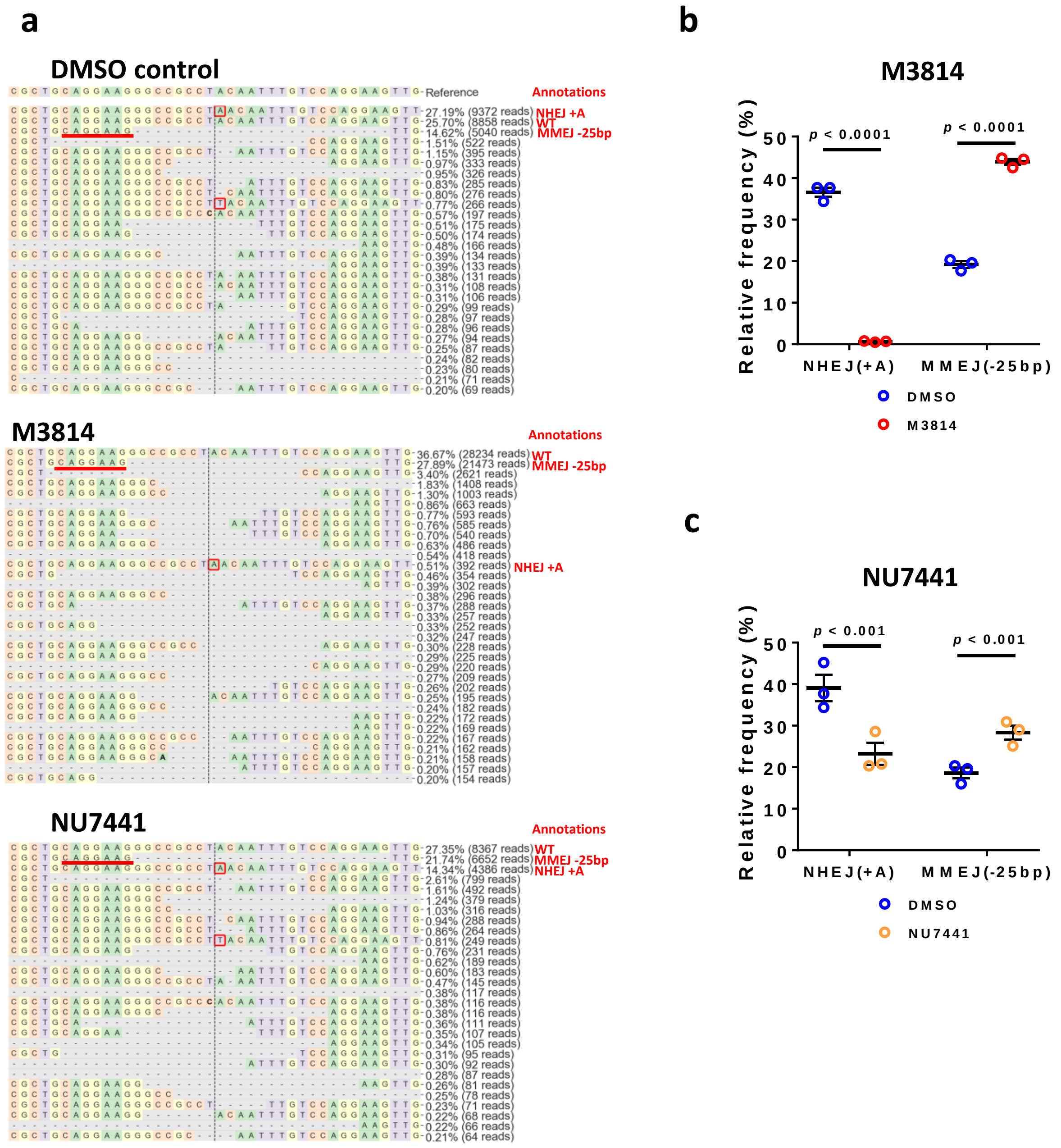

a
b
c
DMSO control
Reference
Annotations
NHEJ +A
WT
MMEJ -25bp
M3814
Annotations
WT
MMEJ -25bp
NHEJ +A
NU7441
Annotations
WT
MMEJ -25bp
NHEJ +A
M3814
Relative frequency (%)
p < 0.0001
p < 0.0001
NHEJ(+A)
MMEJ(-25bp)
DMSO
M3814
NU7441
Relative frequency (%)
p < 0.001
p < 0.001
NHEJ(+A)
MMEJ(-25bp)
DMSO
NU7441

Fig. S10

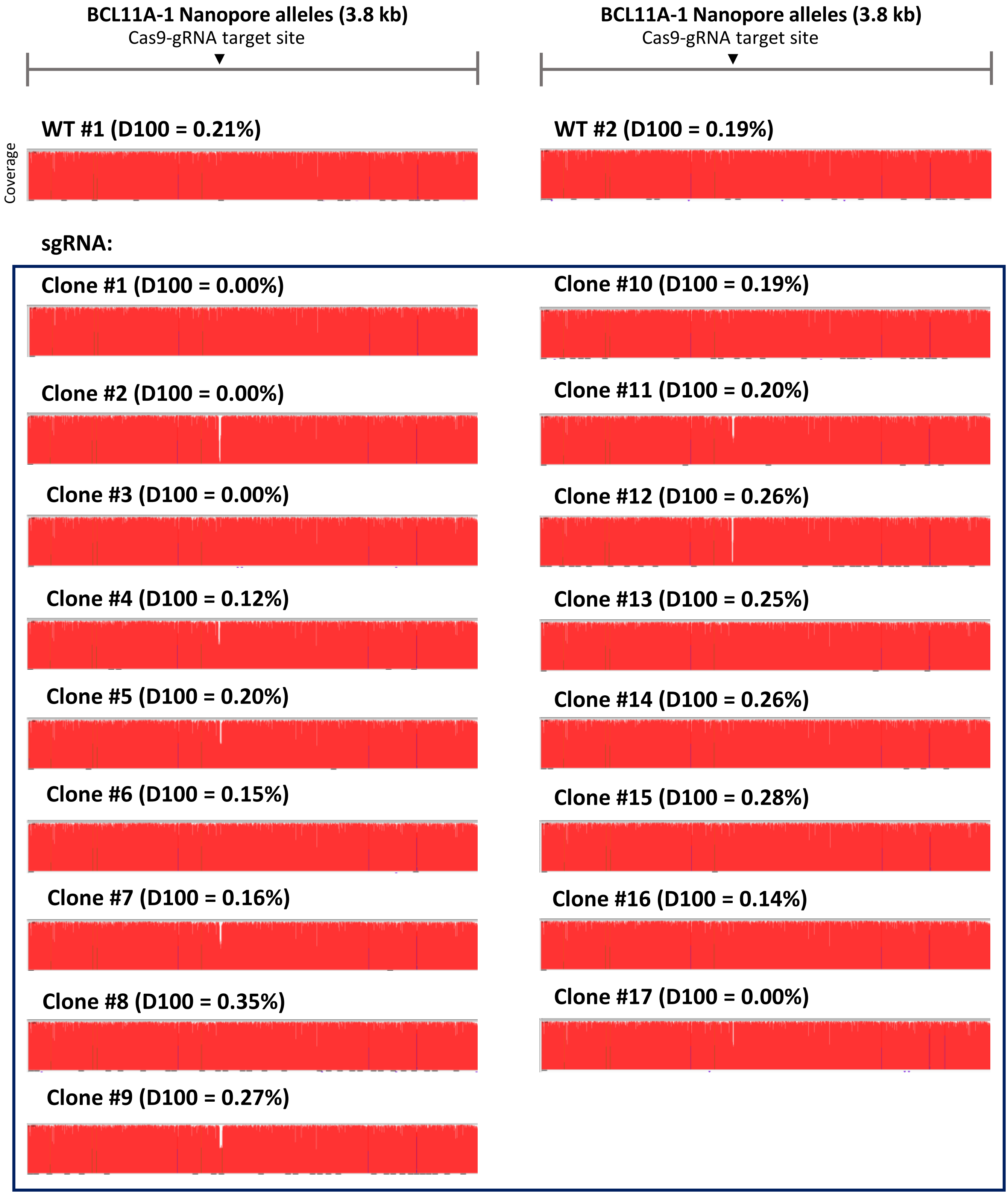
BCL11A-1 Nanopore alleles (3.8 kb)
Cas9-gRNA target site
BCL11A-1 Nanopore alleles (3.8 kb)
Cas9-gRNA target site
WT #1 (D100 = 0.21%)
WT #2 (D100 = 0.19%)
Coverage
sgRNA:
Clone #1 (D100 = 0.00%)
Clone #10 (D100 = 0.19%)
Clone #2 (D100 = 0.00%)
Clone #11 (D100 = 0.20%)
Clone #3 (D100 = 0.00%)
Clone #12 (D100 = 0.26%)
Clone #4 (D100 = 0.12%)
Clone #13 (D100 = 0.25%)
Clone #5 (D100 = 0.20%)
Clone #14 (D100 = 0.26%)
Clone #6 (D100 = 0.15%)
Clone #15 (D100 = 0.28%)
Clone #7 (D100 = 0.16%)
Clone #16 (D100 = 0.14%)
Clone #8 (D100 = 0.35%)
Clone #17 (D100 = 0.00%)
Clone #9 (D100 = 0.27%)

Continued

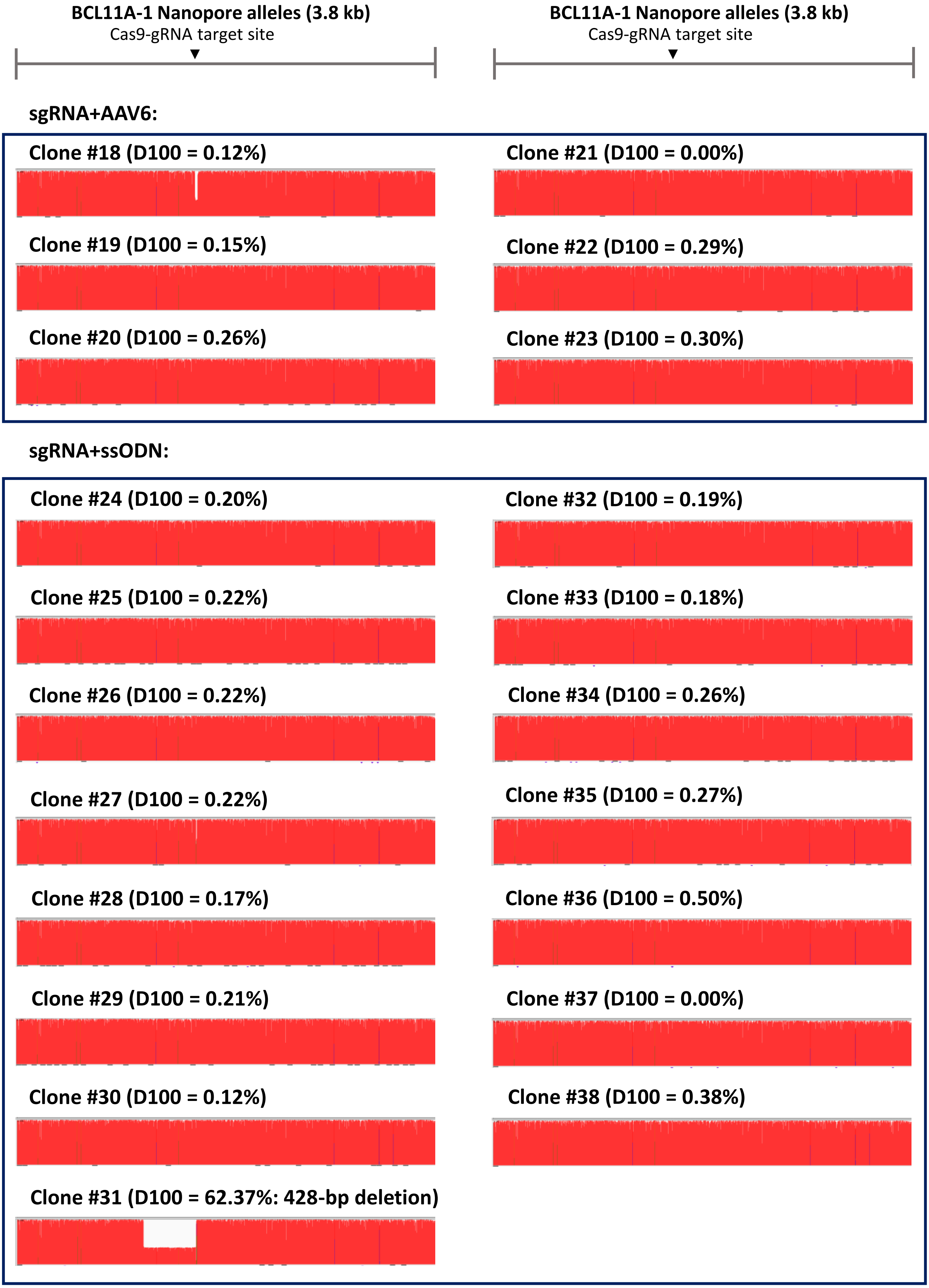

BCL11A-1 Nanopore alleles (3.8 kb)
Cas9-gRNA target site
BCL11A-1 Nanopore alleles (3.8 kb)
Cas9-gRNA target site
sgRNA+AAV6:
Clone #18 (D100 = 0.12%)
Clone #21 (D100 = 0.00%)
Clone #19 (D100 = 0.15%)
Clone #22 (D100 = 0.29%)
Clone #20 (D100 = 0.26%)
Clone #23 (D100 = 0.30%)
sgRNA+ssODN:
Clone #24 (D100 = 0.20%)
Clone #32 (D100 = 0.19%)
Clone #25 (D100 = 0.22%)
Clone #33 (D100 = 0.18%)
Clone #26 (D100 = 0.22%)
Clone #34 (D100 = 0.26%)
Clone #27 (D100 = 0.22%)
Clone #35 (D100 = 0.27%)
Clone #28 (D100 = 0.17%)
Clone #36 (D100 = 0.50%)
Clone #29 (D100 = 0.21%)
Clone #37 (D100 = 0.00%)
Clone #30 (D100 = 0.12%)
Clone #38 (D100 = 0.38%)
Clone #31 (D100 = 62.37%: 428-bp deletion)

Continued

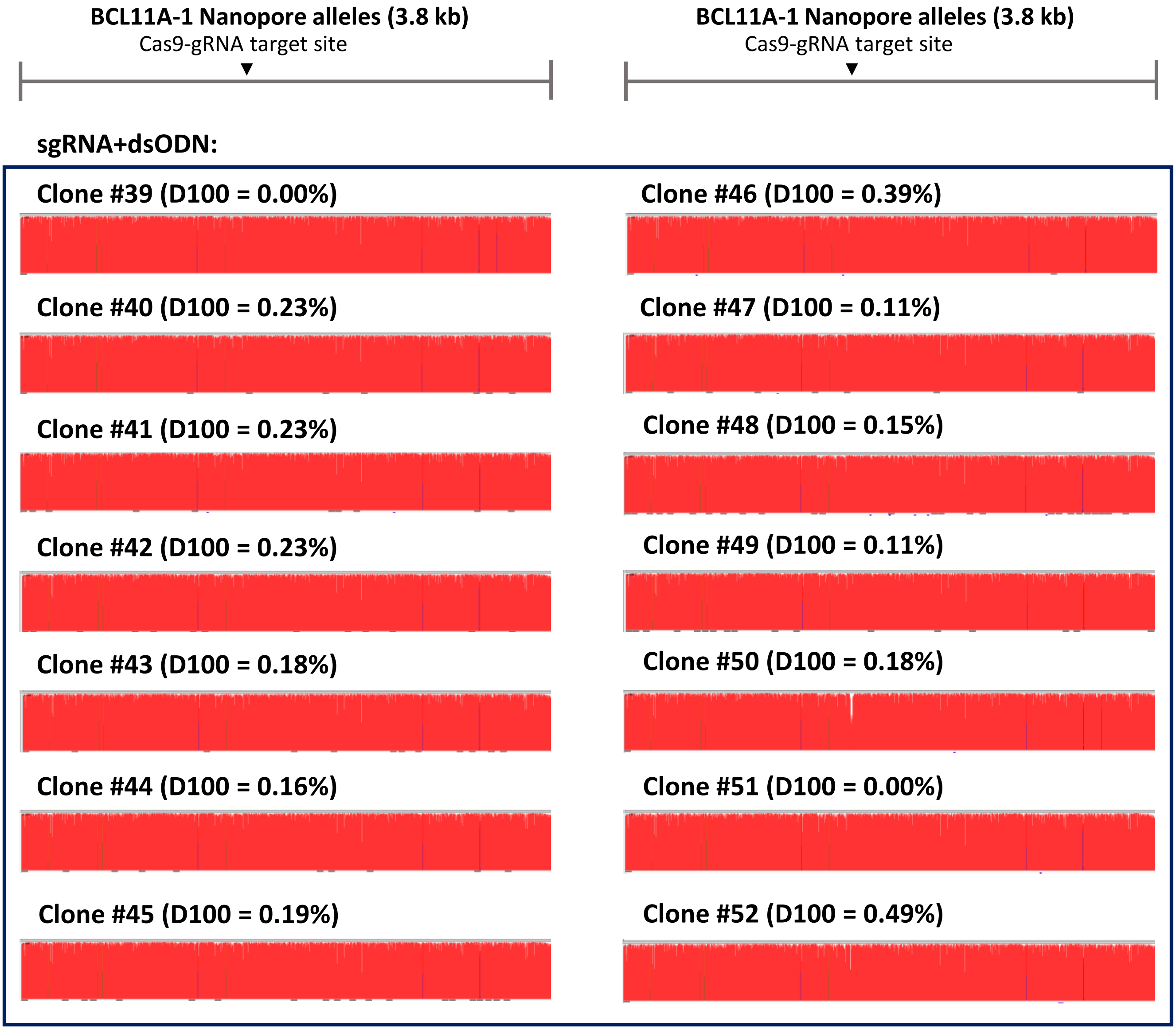
BCL11A-1 Nanopore alleles (3.8 kb)
Cas9-gRNA target site
BCL11A-1 Nanopore alleles (3.8 kb)
Cas9-gRNA target site
sgRNA+dsODN:
Clone #39 (D100 = 0.00%)
Clone #46 (D100 = 0.39%)
Clone #40 (D100 = 0.23%)
Clone #47 (D100 = 0.11%)
Clone #41 (D100 = 0.23%)
Clone #48 (D100 = 0.15%)
Clone #42 (D100 = 0.23%)
Clone #49 (D100 = 0.11%)
Clone #43 (D100 = 0.18%)
Clone #50 (D100 = 0.18%)
Clone #44 (D100 = 0.16%)
Clone #51 (D100 = 0.00%)
Clone #45 (D100 = 0.19%)
Clone #52 (D100 = 0.49%)

Fig. S11

AAV6 HDR donor sequences (Left HA-Insert-Right HA)

EEF2-AAV6

CAGCTTGTGGACCCCCTAAATCACTGAATTCCCAGGGGAGGGGCTCTCCTATCCCCAGTGTGAGAAGGGCTCTGGGCCTGGAGCTCTGAAGGCCTACGCCCTG
GGCCGGTAGAGCAGCCGAGCTGTAGCACAGGGTTGTCCCAAACGAGCAGCGGCATGAGGCCCATGAGTGGCCTGCTAGGCCCTTCGTGAAGTGCTGGGCACC
AGGCCGAGTGTCTGGTCTGCAGGGTGACTCAGGCTGAGGAACTAGCCTGAGCTCCTGACAGGACTTTCCTTCTGCCCTGCCACCTTCTCGATGGCCCAGTGAGC
CTCTCGCTTCCCTCTGCAGGCTTCACCGCTGACCTGAGGTCCAACACGGGCGGCCAGGCGTTCCCCCAGTGTGTGTTTGACCACTGGCAGATCCTGCCCGGAGA
CCCCTTCGACAACAGCAGCCGCCCCAGCCAGGTGGTGGCGGAGACCCGCAAGCGCAAGGGCCTGAAAGAAGGCATCCCTGCCCTGGACAACTTCCTGGACA
AATTGCAGTGTACTAATTATGCTCTCTTGAAATTGGCTGGAGATGTTGAGAGCAACCCAGGTCCCATGGTGAGCAAGGGCGAGGAGGATAACATGGCCTCTCTC
CCAGCGACACATGAGTTACACATCTTTGGCTCCATCAACGGTGTGGACTTTGACATGGTGGGTCAGGGCACCGGCAATCCAAATGATGGTTATGAGGAGTTAAA
CCTGAAGTCCACCAAGGGTGACCTCCAGTTCTCCCCCTGGATTCTGGTCCCTCATATCGGGTATGGCTTCCATCAGTACCTGCCCTACCCTGACGGGATGTCGCC
TTTCCAGGCCGCCATGGTAGATGGCTCCGGATACCAAGTCCATCGCACAATGCAGTTTGAAGATGGTGCCTCCCTTACTGTTAACTACCGCTACACCTACGAGG
GAAGCCACATCAAAGGAGAGGCCCAGGTGAAGGGGACTGGTTTCCCTGCTGACGGTCCTGTGATGACCAACTCGCTGACCGCTGCGGACTGGTGCAGGTCGA
AGAAGACTTACCCCAACGACAAAACCATCATCAGTACCTTTAAGTGGAGTTACACCACTGGAAATGGCAAGCGCTACCGGAGCACTGCGCGGACCACCTACAC
CTTTGCCAAGCCAATGGCGGCTAACTATCTGAAGAACCAGCCGATGTACGTGTTCCGTAAGACGGAGCTCAAGCACTCCAAGACCGAGCTCAACTTCAAGGAG
TGGCAAAAGGCCTTTACCGATGTGATGGGCATGGACGAGCTGTACAAGTAACGCGTGCGGCCCTTCCTGCAGCGCCTGCCGCCCCGGGGACTCGCAGCACCC
ACAGCACCACGTCCTCGAATTCTCAGACGACACCTGGAGACTGTCCCGACACAGCGACGCTCCCCTGAGAGGTTTCTGGGGCCCGCTGCGTGCCATCACTCAA
CCATAACACTTGATGCCGTTTCTTTCAATATTTATTTCCAGAGTCCGGAGGCAGCAGACACGCCCTCTTAGTAGGGACTTAATGGCCGGTCGGGGAGGGGGAG
GCGGGATGGGACACCCAACACTTTTTCCATTTCTTCAGAGGGAAACTCAGATGTCCAAACTAATTTTAACAAACGCATTAAGAGGTTTATTTGGGTACATGGCCC
GCAGTGGCTTTTGCCCCAGAAAGGGGAAAGGAACACGCGGGTAGATGATTTCTAGCAGGCAGGAAGTCCTGTGCGGTGTCACCATGAGCACCTCCAGCTGTAC
TAGTGCCATTGGAATAATAAATTTGATAAGGTGGTGACTCTGTTCTGCATTTTTCACGGTGTCTTCGCAGGGGAGCGGGGCTGCCCAGTACTGGGCTCCCTGGAG
CCTAGAAGGGGACCCGGGCCCT

AAVS1-AAV6

GGGCATCTCTCCTCCCTCACCCAACCCCATGCCGTCTTCACTCGCTGGGTTCCCTTTTCCTTCTCCTTCTGGGGCCTGTGCCATCTCTCGTTTCTTAGGATGGCCTT
CTCCGACGGATGTCTCCCTTGCGTCCCGCCTCCCCTTCTTGTAGGCCTGCATCATCACCGTTTTTCTGGACAACCCCAAAGTACCCCGTCTCCCTGGCTTTAGCCA
CCTCTCCATCCTCTTGCTTTCTTTGCCTGGACACCCCGTTCTCCTGTGGATTCGGGTCACCTCTCACTCCTTTCATTTGGGCAGCTCCCCTACCCCCCTTACCTCTCT
AGTCTGTGCTAGCTCTTCCAGCCCCCTGTCATGGCATCTTCCAGGGGTCCGAGAGCTCAGCTAGTCTTCTTCCTCCAACCCGGGCCCCTATGTCCACTTCAGGAC
AGCATGTTTGCTGCCTCCAGGGATCCTGTGTCCCCGAGCTGGGACCACCTTATATTCCCAGGGCCGGTTAATGTGGCTCTGGTTCTGGGTACTTTTATCTGTCCCC
TCCACCCCACAGTGGGGCCACTAGGGACAGGATTGGTGACAGAAAAGCCCCATCCTTAGGCCTCCTCCTTCCTAGTCTCCTGATATTGGGTCTAACCCACGCGT
AGTTTAAACTTAGGCAGATTCCTTATCTGGTGACACACCCCCATTTCCTGGAGCCATCTCTCTCCTTGCCAGAACCTCTAAGGTTTGCTTACGATGGAGCCAGAG
AGGATCCTGGGAGGGAGAGCTTGGCAGGGGGTGGGAGGGAAGGGGGGGATGCGTGACCTGCCCGGTTCTCAGTGGCCACCCTGCGCTACCCTCTCCCAGAA
CCTGAGCTGCTCTGACGCGGCCGTCTGGTGCGTTTCACTGATCCTGGTGCTGCAGCTTCCTTACACTTCCCAAGAGGAGAAGCAGTTTGGAAAAACAAAATCAG
AATAAGTTGGTCCTGAGTTCTAACTTTGGCTCTTCACCTTTCTAGTCCCCAATTTATATTGTTCCTCCGTGCGTCAGTTTTACCTGTGAGATAAGGCCAGTAGCCAG
CCCCGTCCTGGCAGGGCTGTGGTGAGGAGGGGGGTGTCCGTGTGGAAAACTCCCTTTGTGAGAATGGTGCGTCCTAGGTGTTCACCAGGTCGTGGCCGCCTCT
ACTCCCTTTCTCTTTCTCCATCCTTCTTTCCTTAAAGAGTCCCCAGTGCTATCTGGGACATATTCCTCCGCCCAGAGCAGGGTCCCGCTTCCCTAAGGCCCTG

BCL11A-1-AAV6

GGGCAATACAGACTGGTTCTGTGATGACAAATAACTCCTAGCTCATTCCTAATGATTTATCACCAAATGTTCTTTCTTCAGCTGGAATTTAAAATATGGACTCATC
CGTAAAATAGGAATAATAATAGTATATGCTTCATAGGGTTTGTATGAAAATAAAATGAGTGCGTATTTGTAAAGTTCCTAGAGCAGAGTAAGTGCTCCGAGCTTG
TGAACTAAAATGCTGCCTCCTGGTATTTATTAGTTACACCTCAGCAGAAACAAAGTTATCAGGCCCTTTCCCCAATTCCTAGTTTGGGTCAGAAGAAAAGGGAAA
AGGGAGAGGAAAAAGGAAAAGAATATGACGTCAGGGGGAGGCAAGTCAGTTGGGAACACAGATCCTAACACAGTAGCTGGTACCTGATAGGTGCCTATATGT
GATGGATGGGTGGACAGCCCGACAGATGAAAAATGGACAATTATGAGGAGGGGAGAGTGCAGACAGGGGAAGCTTCACCTCCTTTACAATTTTGGGAGTCCA
CACGGCATGGCATACAAATTATTTCATTCCCATTGAGAAATAAAATCCAATTCTCCATCACCAAGAGAGCCTTCCGAAAGAGGCCCCCCTGGGCAAACGGCCAC
CGATGGAGAGGTCTGCCAGTCCTCTTCTACCCCACCCACGCCCCCACCCTAATCAGAACGCGTAGTTTAAACCTGTGATAAAAGCAACTGTTAGCTTGCACTAG
ACTAGCTTCAAAGTTGTATTGACCCTGGTGTGTTATGTCTAAGAGTAGATGCCATATCTCTTTTCTGGCCTATGTTATTACCTGTATGGACTTTGCACTGGAATCAG
CTATCTGCTCTTACTTATGCACACCTGGGGCATAGAGCCAGCCCTGTATCGCTTTTCAGCCATCTCACTACAGATAACTCCCAAGTCCTGTCTAGCTGCCTTCCTT
ATCACAGGAATAGCACCCAAGGTCCATCAGTACCTCAGAGTAGAACCCCCTATAAACTAGTCTGGTTTGCCCATGGGGCACAGTCAGGCTGTTTTCCAGGGTGG
GGTGCAGACATTCTCTGCCTGTTGTGATGCTTACATATAACGTCATAACAGACACACGTATGTGTTGTGATCCCTGTGGTTTGAGAGTTTGGAGCTTCCCTAAAAG
TCAAAATATTCTCAATGGGCCCTCAATCAGCACATACACACAAAAGGTACCTGGAAAACTGTAATTCTTTTCCTGCTCAAAGACAGGCAATTCAATACCCCTTCC
CCCAACCAAAAACCCT

BCL11A-2-AAV6

GGCTACACTTCCTTTTCCTTTCCTCTCCATTTCATCCCTTTCCAAAAAGTGTTTAGACAAATAGTTTCCCAGACTTGGTTTTATCATGCTGGGTTGACAAAGGTTGTG
TACAGAGCTGGAATAATTTTTTCTTCTTTCTACTGTTGGCACATCAATATCTTTTTTTCTGCAAAGAAGGGGCTAAGCTTGCACGAAAACACCGGTGGGAACCCAC
TGGAGAAGGGCATGGGTGGTTTTTGGTTCGGTTGGTGGCTGATCTGATGGGTGGTATAGGCAGAATGTCTGGCTGCCCCTTCAGATTCCTGTAGATTCCGTAGGC
GACCAACATGGGTTTAAACGGTGGGGGTGGGGGACTGTCTGCTCTTTTTGATGCAAATATCTTCACTCTCCTTGGTGCTTCTGTGGTTGTGCCTATGAATTTAGGA
CCTTATGCCTCATAGTGTTGAGTCAGAGCAAAACAGTTTACATCAAAGGCCACTTATTTAATTTTCCTCAGAAAACTCTGTGAGGATAATTTCTCCTTCTTATAAAC
GGAATGCGAATGTGTACACAAATGTGTACATTATCCCACCGAAGGGTGATGCTCTTTACTTTTATAATCTAGATTTTAAAAAATTTGGTGGCACTTGATTTCAGGT
CCTCAGACTCCAGAATTTATACTCACTGATTAGTAATGCATTATGCAAATAATTCAGTTTACATATCCAAATACTGTTTTGCATCATTCACACCTCATGTCTTGAAA
AAAAAAGTGAAATGTTTCCTCAAAATAAACAAAACCTGCAGAAGAGTTTATTGTAAGGGTGAAAATTAAAGAGGAACCCATTTGCCTGGGAACTTTTGAGGAAA
ATTTTAATACTCCTGCATAAATAATTTTGCAATTATTTTTGATTCCTACCTAATCAATATACCTTGCCACGCAAGGTATATTCTATTATCTTTGGCTTTCTGAATTTTT
CAATTTTCAGAAAAAGAAGCC

Table S1. gRNA sequences

Site	gRNA sequences
EEF2	CTTCCTGGACAAATTGTAGG
AAVS1	TAAGGAATCTGCCTAACAGG
BCL11A-1	CTAACAGTTGCTTTTATCAC
BCL11A-2	GTAGGCGACCAACATGGGGT

Table S2. ssODN sequences for HDR editing
(* indicates phosphorothioate bond)

Site	ssODN sequences
EEF2	G*C*C*TGAAAGAAGGCATCCCTGCCCTGGACAACTTCCTGGACAAATTGTAGTACTAAGCGGCCCTTCCTGCAGCGCCTGCCGCCCCGGGGACTCGCAGCACCCA*C*A*G
AAVS1	C*C*C*ATCCTTAGGCCTCCTCCTTCCTAGTCTCCTGATATTGGGTCTAACCCGAATTCTTAGGCAGATTCCTTATCTGGTGACACACCCCCATTTCCTGGAGCCA*T*C*T
BCL11A-1	A*C*G*GCCACCGATGGAGAGGTCTGCCAGTCCTCTTCTACCCCACCCACGCCCCCACCCTAATCAGAGGCCAAACCCTTCCTGGAGCCTGTGGGTGTGGCACAACAGGGTATAAAAGCAACTGTTAGCTTGCACTAGACTAGC*T*T*C
BCL11A-2	G*T*C*TGGCTGCCCCTTCAGATTCCTGTAGATTCCGTAGGCGACCAACATGGGAATTCGGTGGGGGTGGGGGACTGTCTGCTCTTTTTGATGCAAATATCTTCAC*T*C*T

Table S3. EEF2 (long-range 5287 bp) forward primers with barcodes (BC, in red) and reverse primer

ID	Primer sequences (5'-3')
EEF2-F-BC1	ATGATAACTAGGAAGTCTTGGGCTCCTCAGTC
EEF2-F-BC2	CATCTCATCTCGAAGTCTTGGGCTCCTCAGTC
EEF2-F-BC3	CCTGAAGACGTTAAGTCTTGGGCTCCTCAGTC
EEF2-F-BC4	GCAGAAGTCTATAAGTCTTGGGCTCCTCAGTC
EEF2-F-BC5	GTTCAGTAAGACAAGTCTTGGGCTCCTCAGTC
EEF2-F-BC6	TACCAGTCAATCAAGTCTTGGGCTCCTCAGTC
EEF2-F-BC7	TGCTCATCTGCTAAGTCTTGGGCTCCTCAGTC
EEF2-F-BC8	TGTTGGAACTTCAAGTCTTGGGCTCCTCAGTC
EEF2-F-BC9	AGGAGACATCAGAAGTCTTGGGCTCCTCAGTC
EEF2-F-BC10	AACGACTATGTCAAGTCTTGGGCTCCTCAGTC
EEF2-F-BC11	GTAGCTGATTCCAAGTCTTGGGCTCCTCAGTC
EEF2-F-BC12	TAAGACACTCGTAAGTCTTGGGCTCCTCAGTC
EEF2-F-BC13	GATACATATCGAAAGTCTTGGGCTCCTCAGTC
EEF2-F-BC14	TTCTGGTGACTGAAGTCTTGGGCTCCTCAGTC
EEF2-F-BC15	AGTTGGAGGTTCAAGTCTTGGGCTCCTCAGTC
EEF2-F-BC16	ACTCGTCGTCTAAAGTCTTGGGCTCCTCAGTC
EEF2-F-BC17	AAAGATCCAAGTCTTGGGCTCCTCAGTC
EEF2-F-BC18	AATATGCTAAGTCTTGGGCTCCTCAGTC

EEF2-F-BC19	ACTCGAGTAAGTCTTGGGCTCCTCAGTC
EEF2-F-BC20	AGATGATGAAGTCTTGGGCTCCTCAGTC
EEF2-F-BC21	ATTGTACGAAGTCTTGGGCTCCTCAGTC
EEF2-F-BC22	CCACTGACAAGTCTTGGGCTCCTCAGTC
EEF2-F-BC23	GATCTTACAAGTCTTGGGCTCCTCAGTC
EEF2-F-BC24	GGAGCGATAAGTCTTGGGCTCCTCAGTC
EEF2-F-BC25	ACGCCACGTTAAGTCTTGGGCTCCTCAGTC
EEF2-F-BC26	CACTCTCAGGAAGTCTTGGGCTCCTCAGTC
EEF2-F-BC27	CAGTGACCAGAAGTCTTGGGCTCCTCAGTC
EEF2-F-BC28	CCAGGCTCTTAAGTCTTGGGCTCCTCAGTC
EEF2-F-BC29	AGATTACTCTAAGTCTTGGGCTCCTCAGTC
EEF2-F-BC30	CGACTAGACCAAGTCTTGGGCTCCTCAGTC
EEF2-F-BC31	GATCTGACAGAAGTCTTGGGCTCCTCAGTC
EEF2-F-BC32	GCAACTGTCTAAGTCTTGGGCTCCTCAGTC
EEF2-Reverse	GACCAACCAGGCCAAGCAAA

Table S4. AAVS1 (long-range 3928 bp) forward primers with barcodes (BC, in red) and reverse primer

ID	Primer sequences (5'-3')
AAVS1-F-BC1	AAGAGGAGTGCAAACAGGAAGTGAACGG
AAVS1-F-BC2	CTGGAAATTGCAAACAGGAAGTGAACGG
AAVS1-F-BC3	CTTGTTGGTGCAAACAGGAAGTGAACGG
AAVS1-F-BC4	TATGCGTTTGCAAACAGGAAGTGAACGG
AAVS1-F-BC5	TGTTCAAGTGCAAACAGGAAGTGAACGG
AAVS1-F-BC6	AAACTTTGTGCAAACAGGAAGTGAACGG
AAVS1-F-BC7	GGACCGATTGCAAACAGGAAGTGAACGG
AAVS1-F-BC8	AATTATCCTGCAAACAGGAAGTGAACGG
AAVS1-F-BC9	AAACAAACTGCAAACAGGAAGTGAACGG
AAVS1-F-BC10	ACCGCCTATGCAAACAGGAAGTGAACGG
AAVS1-F-BC11	CCAGGTTCTGCAAACAGGAAGTGAACGG
AAVS1-F-BC12	CCTAACGCTGCAAACAGGAAGTGAACGG
AAVS1-F-BC13	CGCTCTTCTGCAAACAGGAAGTGAACGG
AAVS1-F-BC14	GGGTAACGTGCAAACAGGAAGTGAACGG
AAVS1-F-BC15	TATACGACTGCAAACAGGAAGTGAACGG
AAVS1-F-BC16	AACGGACTTGCAAACAGGAAGTGAACGG
AAVS1-F-BC17	GACAACGCTGCAAACAGGAAGTGAACGG
AAVS1-F-BC18	TTAGTCCGTGCAAACAGGAAGTGAACGG
AAVS1-F-BC19	ATTCCGGATGCAAACAGGAAGTGAACGG
AAVS1-F-BC20	CCGACATCTGCAAACAGGAAGTGAACGG
AAVS1-F-BC21	ACCGTTTATGCAAACAGGAAGTGAACGG
AAVS1-F-BC22	GGGTAACGATTGCAAACAGGAAGTGAACGG
AAVS1-F-BC23	TATACGACTATGCAAACAGGAAGTGAACGG
AAVS1-F-BC24	AACGGACTAGTGCAAACAGGAAGTGAACGG
AAVS1-F-BC25	GACAACGCAATGCAAACAGGAAGTGAACGG

AAVS1-F-BC26	ATGATAACTAGGTGCAAACAGGAAGTGAACGG
AAVS1-F-BC27	CATCTCATCTCGTGCAAACAGGAAGTGAACGG
AAVS1-F-BC28	CCTGAAGACGTTTGCAAACAGGAAGTGAACGG
AAVS1-F-BC29	GCAGAAGTCTATTGCAAACAGGAAGTGAACGG
AAVS1-F-BC30	GTTCAGTAAGACTGCAAACAGGAAGTGAACGG
AAVS1-F-BC31	TACCAGTCAATCTGCAAACAGGAAGTGAACGG
AAVS1-F-BC32	TGCTCATCTGCTTGCAAACAGGAAGTGAACGG
AAVS1-F-BC33	TGTTGGAACTTCTGCAAACAGGAAGTGAACGG
AAVS1-F-BC34	AGGAGACATCAGTGCAAACAGGAAGTGAACGG
AAVS1-F-BC35	AACGACTATGTCTGCAAACAGGAAGTGAACGG
AAVS1-F-BC36	GTAGCTGATTCCTGCAAACAGGAAGTGAACGG
AAVS1-F-BC37	TAAGACACTCGTTGCAAACAGGAAGTGAACGG
AAVS1-F-BC38	GATACATATCGATGCAAACAGGAAGTGAACGG
AAVS1-F-BC39	TTCTGGTGACTGTGCAAACAGGAAGTGAACGG
AAVS1-F-BC40	AGTTGGAGGTTCTGCAAACAGGAAGTGAACGG
AAVS1-F-BC41	ACTCGTCGTCTATGCAAACAGGAAGTGAACGG
AAVS1-Reverse	CGACCTACTCTCTTCCGCAT

Table S5. BCL11A-1 (long-range 3863 bp) forward primers with barcodes (BC, in red) and reverse primer

ID	Primer sequences (5’-3’)
BCL11A-1-F-BC1	AAGAGGAGGTGTGGTGTTCGGAGTCCTA
BCL11A-1-F-BC2	CTGGAAATGTGTGGTGTTCGGAGTCCTA
BCL11A-1-F-BC3	CTTGTTGGGTGTGGTGTTCGGAGTCCTA
BCL11A-1-F-BC4	TATGCGTTGTGTGGTGTTCGGAGTCCTA
BCL11A-1-F-BC5	TGTTCAAGGTGTGGTGTTCGGAGTCCTA
BCL11A-1-F-BC6	AAACTTTGGTGTGGTGTTCGGAGTCCTA
BCL11A-1-F-BC7	GGACCGATGTGTGGTGTTCGGAGTCCTA
BCL11A-1-F-BC8	AATTATCCGTGTGGTGTTCGGAGTCCTA
BCL11A-1-F-BC9	AAACAAACGTGTGGTGTTCGGAGTCCTA
BCL11A-1-F-BC10	ACCGCCTAGTGTGGTGTTCGGAGTCCTA
BCL11A-1-F-BC11	CCAGGTTCGTGTGGTGTTCGGAGTCCTA
BCL11A-1-F-BC12	CCTAACGCGTGTGGTGTTCGGAGTCCTA
BCL11A-1-F-BC13	CGCTCTTCGTGTGGTGTTCGGAGTCCTA
BCL11A-1-F-BC14	TTAGTCCGGTGTGGTGTTCGGAGTCCTA
BCL11A-1-F-BC15	GACAACGCGTGTGGTGTTCGGAGTCCTA
BCL11A-1-F-BC16	GGGTAACGGTGTGGTGTTCGGAGTCCTA
BCL11A-1-F-BC17	TATACGACGTGTGGTGTTCGGAGTCCTA
BCL11A-1-F-BC18	AACGGACTGTGTGGTGTTCGGAGTCCTA
BCL11A-1-F-BC19	AGACTCGTGTGTGGTGTTCGGAGTCCTA
BCL11A-1-F-BC20	ATTCCGGAGTGTGGTGTTCGGAGTCCTA
BCL11A-1-F-BC21	ACTCGAGAGTGTGGTGTTCGGAGTCCTA
BCL11A-1-F-BC22	TTAGTCCGACGTGTGGTGTTCGGAGTCCTA
BCL11A-1-F-BC23	GACAACGCTTGTGTGGTGTTCGGAGTCCTA

BCL11A-1-F-BC24	GGGTAACGTAGTGTGGTGTTCGGAGTCCTA
BCL11A-1-F-BC25	TATACGACATGTGTGGTGTTCGGAGTCCTA
BCL11A-1-F-BC26	ACGCCACGTTGTGTGGTGTTCGGAGTCCTA
BCL11A-1-F-BC27	CACTCTCAGGGTGTGGTGTTCGGAGTCCTA
BCL11A-1-F-BC28	CAGTGACCAGGTGTGGTGTTCGGAGTCCTA
BCL11A-1-F-BC29	CCAGGCTCTTGTGTGGTGTTCGGAGTCCTA
BCL11A-1-F-BC30	AGATTACTCAGTGTGGTGTTCGGAGTCCTA
BCL11A-1-F-BC31	CGACTAGACCGTGTGGTGTTCGGAGTCCTA
BCL11A-1- Reverse	AGGAGCGGCAGTTTAAGTCT

Table S6. BCL11A-2 (long-range 5313 bp) forward primers with barcodes (BC, in red) and reverse primer

ID	Primer sequences (5'-3')
BCL11A-2-F-BC1	AAGAGGAGGCTGTGCTTTCTTCTATGATTCCTC
BCL11A-2-F-BC2	CTGGAAATGCTGTGCTTTCTTCTATGATTCCTC
BCL11A-2-F-BC3	CTTGTTGGGCTGTGCTTTCTTCTATGATTCCTC
BCL11A-2-F-BC4	TATGCGTTGCTGTGCTTTCTTCTATGATTCCTC
BCL11A-2-F-BC5	TGTTCAAGGCTGTGCTTTCTTCTATGATTCCTC
BCL11A-2-F-BC6	AAACTTTGGCTGTGCTTTCTTCTATGATTCCTC
BCL11A-2-F-BC7	GGACCGATGCTGTGCTTTCTTCTATGATTCCTC
BCL11A-2-F-BC8	AATTATCCGCTGTGCTTTCTTCTATGATTCCTC
BCL11A-2-F-BC9	AAACAAACGCTGTGCTTTCTTCTATGATTCCTC
BCL11A-2-F-BC10	ACCGCCTAGCTGTGCTTTCTTCTATGATTCCTC
BCL11A-2-F-BC11	CCAGGTTCGCTGTGCTTTCTTCTATGATTCCTC
BCL11A-2-F-BC12	CCTAACGCGCTGTGCTTTCTTCTATGATTCCTC
BCL11A-2-F-BC13	CGCTCTTCGCTGTGCTTTCTTCTATGATTCCTC
BCL11A-2-F-BC14	GATCTGACAGGCTGTGCTTTCTTCTATGATTCCTC
BCL11A-2-F-BC15	GCAACTGTCTGCTGTGCTTTCTTCTATGATTCCTC
BCL11A-2-F-BC16	GCTGATCGGAGCTGTGCTTTCTTCTATGATTCCTC
BCL11A-2-F-BC17	GACGACTAAGGCTGTGCTTTCTTCTATGATTCCTC
BCL11A-2-F-BC18	GTCCGGTGAAGCTGTGCTTTCTTCTATGATTCCTC
BCL11A-2-F-BC19	GTGCATATCCGCTGTGCTTTCTTCTATGATTCCTC
BCL11A-2-F-BC20	TAAGGAATCCGCTGTGCTTTCTTCTATGATTCCTC
BCL11A-2-F-BC21	TATGCTATGCGCTGTGCTTTCTTCTATGATTCCTC
BCL11A-2-F-BC22	TCGCGACACTGCTGTGCTTTCTTCTATGATTCCTC
BCL11A-2-F-BC23	TTCGGATGGTGCTGTGCTTTCTTCTATGATTCCTC
BCL11A-2-Reverse	TGAAATCTCCCTTCTTTACGGTTCT

Table S7. EEF2 (PE150) forward primers with barcodes (BC, in red) and reverse primer

ID	Primer sequences (5'-3')
EEF2-F-BC1	TCACGCAGTCTCCAGGTGTCGTCTG
EEF2-F-BC2	GATGTCAGTCTCCAGGTGTCGTCTG
EEF2-F-BC3	TAGGCCAGTCTCCAGGTGTCGTCTG
EEF2-F-BC4	GACCACAGTCTCCAGGTGTCGTCTG

EEF2-F-BC5	CAGTGCAGTCTCCAGGTGTCGTCTG
EEF2-F-BC6	CCAATCAGTCTCCAGGTGTCGTCTG
EEF2-F-BC7	AGATCCAGTCTCCAGGTGTCGTCTG
EEF2-F-BC8	CTTGACAGTCTCCAGGTGTCGTCTG
EEF2-F-BC9	AGTTGCAGTCTCCAGGTGTCGTCTG
EEF2-F-BC10	GCATACAGTCTCCAGGTGTCGTCTG
EEF2-F-BC11	CTATCCAGTCTCCAGGTGTCGTCTG
EEF2-F-BC12	ACTCGCAGTCTCCAGGTGTCGTCTG
EEF2-F-BC13	GGCATCAGTCTCCAGGTGTCGTCTG
EEF2-F-BC14	GTCTTCAGTCTCCAGGTGTCGTCTG
EEF2-F-BC15	TGACTCAGTCTCCAGGTGTCGTCTG
EEF2-F-BC16	TTATTCAGTCTCCAGGTGTCGTCTG
EEF2- Reverse	GTTTGACCACTGGCAGATCC

Table S8. AAVS1 (PE150) forward primers with barcodes (BC, in red) and reverse primer

ID	Primer sequences (5'-3')
AAVS1-F-BC1	TCACGGGCAAGGAGAGAGATGGC
AAVS1-F-BC2	GATGTGGCAAGGAGAGAGATGGC
AAVS1-F-BC3	TAGGCGGCAAGGAGAGAGATGGC
AAVS1-F-BC4	GACCAGGCAAGGAGAGAGATGGC
AAVS1-F-BC5	CAGTGGGCAAGGAGAGAGATGGC
AAVS1-F-BC6	CCAATGGCAAGGAGAGAGATGGC
AAVS1-F-BC7	AGATCGGCAAGGAGAGAGATGGC
AAVS1-F-BC8	CTTGAGGCAAGGAGAGAGATGGC
AAVS1-F-BC9	AGTTGGGGCAAGGAGAGAGATGGC
AAVS1-F-BC10	GCATATGGCAAGGAGAGAGATGGC
AAVS1-F-BC11	CTATCAGGCAAGGAGAGAGATGGC
AAVS1-F-BC12	ACTCGTGGCAAGGAGAGAGATGGC
AAVS1-F-BC13	GGCATTGGCAAGGAGAGAGATGGC
AAVS1-F-BC14	GTCTTGGGCAAGGAGAGAGATGGC
AAVS1-F-BC15	TGACTGGGCAAGGAGAGAGATGGC
AAVS1-F-BC16	TTATTTGGCAAGGAGAGAGATGGC
AAVS1-Reverse	GGCTCTGGTTCTGGGTACTT

Table S9. BCL11A-1 (PE150) forward primers with barcodes (BC, in red) and reverse primer

ID	Primer sequences (5'-3')
BCL11A-1-F-BC1	TCACGTCCATCACCAAGAGAGCCT
BCL11A-1-F-BC2	GATGTTCCATCACCAAGAGAGCCT
BCL11A-1-F-BC3	TAGGCTCCATCACCAAGAGAGCCT
BCL11A-1-F-BC4	GACCATCCATCACCAAGAGAGCCT
BCL11A-1-F-BC5	CAGTGTCCATCACCAAGAGAGCCT
BCL11A-1-F-BC6	CCAATTCCATCACCAAGAGAGCCT
BCL11A-1-F-BC7	AGATCTCCATCACCAAGAGAGCCT

BCL11A-1-F-BC8	CTTGATCCATCACCAAGAGAGCCT
BCL11A-1-F-BC9	AGTTGGTCCATCACCAAGAGAGCCT
BCL11A-1-F-BC10	GCATATTCCATCACCAAGAGAGCCT
BCL11A-1-F-BC11	CTATCATCCATCACCAAGAGAGCCT
BCL11A-1-F-BC12	ACTCGTTCCATCACCAAGAGAGCCT
BCL11A-1-F-BC13	GGCATTTCCATCACCAAGAGAGCCT
BCL11A-1-F-BC14	GTCTTGTCCATCACCAAGAGAGCCT
BCL11A-1-F-BC15	TGACTGTCCATCACCAAGAGAGCCT
BCL11A-1-F-BC16	TTATTTTCCATCACCAAGAGAGCCT
BCL11A-1- Reverse	AGACATAACACACCAGGGTCA

Table S10. BCL11A-2 (PE150) forward primers with barcodes (BC, in red) and reverse primer

ID	Primer sequences (5'-3')
BCL11A-2-F-BC17	AAACAGCACAACCACAGAAGCACCAA
BCL11A-2-F-BC18	ATTCCGCACAACCACAGAAGCACCAA
BCL11A-2-F-BC19	CCGACACACAACCACAGAAGCACCAA
BCL11A-2-F-BC20	AGCTTGCACAACCACAGAAGCACCAA
BCL11A-2-F-BC21	ATGAAGCACAACCACAGAAGCACCAA
BCL11A-2-F-BC22	CACCTCCACAACCACAGAAGCACCAA
BCL11A-2-F-BC23	GACAACCACAACCACAGAAGCACCAA
BCL11A-2-F-BC24	CGAGGTCACAACCACAGAAGCACCAA
BCL11A-2-F-BC25	GTCTGACACAACCACAGAAGCACCAA
BCL11A-2-F-BC26	GAAGGCCACAACCACAGAAGCACCAA
BCL11A-2-F-BC27	GGGATTCACAACCACAGAAGCACCAA
BCL11A-2-F-BC28	GGTTCTCACAACCACAGAAGCACCAA
BCL11A-2-F-BC29	TCCTTGCACAACCACAGAAGCACCAA
BCL11A-2-F-BC30	AAAGTTCACAACCACAGAAGCACCAA
BCL11A-2-F-BC31	AAGCATCACAACCACAGAAGCACCAA
BCL11A-2-F-BC32	TTCTATCACAACCACAGAAGCACCAA
BCL11A-2-F-BC33	TCACGCACAACCACAGAAGCACCAA
BCL11A-2-F-BC34	GATGTCACAACCACAGAAGCACCAA
BCL11A-2-F-BC35	TAGGCCACAACCACAGAAGCACCAA
BCL11A-2-F-BC36	GACCACACAACCACAGAAGCACCAA
BCL11A-2-F-BC37	CAGTGCACAACCACAGAAGCACCAA
BCL11A-2-F-BC38	CCAATCACAACCACAGAAGCACCAA
BCL11A-2-F-BC39	AGATCCACAACCACAGAAGCACCAA
BCL11A-2-F-BC40	CTTGACACAACCACAGAAGCACCAA
BCL11A-2-F-BC41	AGTTGGCACAACCACAGAAGCACCAA
BCL11A-2-F-BC42	GCATATCACAACCACAGAAGCACCAA
BCL11A-2-F-BC43	CTATCACACAACCACAGAAGCACCAA
BCL11A-2-F-BC44	ACTCGTCACAACCACAGAAGCACCAA
BCL11A-2-F-BC45	GGCATTCACAACCACAGAAGCACCAA
BCL11A-2-F-BC46	GTCTTGCACAACCACAGAAGCACCAA

BCL11A-2-F-BC47	TGACTGCACAACCACAGAAGCACCAA
BCL11A-2-F-BC48	TTATTTCACAACCACAGAAGCACCAA
BCL11A-2-Reverse	GCTTGCACGAAAACACCGGT

Table S11. Primers for qPCR analysis to detect mega-deletions (up to 160 kbp) after cleavage at BCL11A-1

ID	Primer sequences (5’-3’)
BCL11A-1-q-160-F	GTCATAGAAACTTCCTGTGC
BCL11A-1-q-160-R	CCACAAAGTTAGAGCTAGTG
BCL11A-1-q-80-F	TCACAATCAGGGAAAGTTGC
BCL11A-1-q-80-R	ACCTCAGTCTTTCCAGTTCT
BCL11A-1-q-40-F	GCATCAGTTCAGGTATGTAG
BCL11A-1-q-40-R	CATGAGAACCTAGCCAACAT
BCL11A-1-q+40-F	CAAGGTCTGGTTTACTTCTG
BCL11A-1-q+40-R	AATCCAGGTTCCCACGATAC
BCL11A-1-q+80-F	TGAACACCTATGGTCTGACT
BCL11A-1-q+80-R	GTGAAGGCTGATGTTTCGAG
BCL11A-1-q+160-F	CTAACTCCTGGCTTGTTGCA
BCL11A-1-q+160-R	CATTCACAGAAGGTGAGGGT
AAVS1-F	GGCAAGGAGAGAGATGGC
AAVS1-R	GGCTCTGGTTCTGGGTACTT
β-ACTIN-F	TCGTGCGTGACATTAAGGAG
β-ACTIN-R	GGCAGCTCGTAGCTCTTCTC

Reference sequences for nanopore sequencing data alignment

EEF2:

AAGTCTTGGGCTCCTCAGTCGGGAGCTAGAAGTAAGGAGCATAGAAGGAATATAGGGTGA
TACTTGTGCCAGCCACAACTTTTGTTCTTAGCCCAGTGAGGGCATTTCTGGAGAAGCTCC
AAGCCCCAGGGCACAGCACACCCCTGCTCATCTGTGTCCCTGGGCACAGGTGGGTGGCGG
CCGTCGACCCATACCCAGGGAGGAGGGCAGTGATTCTGAAGAGGATGTTTCCTGACAGCC
CCGACGCAGCAGTCAGGGCCTTGTGGAAGCCACCAGTCTGCCCTGGGACCTGGCAGGAGG
GCCACCAACTCCGGGAGGGACCTGCCCTGGCCCTCCAGGGTCCAGCACAACCATCCCTGC
GGCCTGCTGCCGCCTTCTGCTTCTTCCAGAACAATCTTGATGATGGGCCGCTACGTGGAG
CCCATCGAGGATGTGCCTTGTGGGAACATTGTGGGCCTCGTGGGCGTGGACCAGTTCCTG
GTGAAGACGGGCACCATCACCACCTTCGAGCACGCGCACAACATGCGGGTGATGAAGTTC
AGCGTCAGCCCTGTTGTCAGAGTGGCCGTGGAGGCCAAGAACCCGGCTGACCTGCCCAAG
CTGGTGGAGGGGCTGAAGCGGCTGGCCAAGTCCGACCCCATGGTGCAGGTGGGCACGGAG
TGCACCTGCTGGGAGCAGACACCCTGAGGGGGGACGGCTTGTGTCCTGAGTTGTGGCTGT
GTGGGTTGCTGTGGTTGGTGGATTGGGGTGACTGGTCATGAAAGGAGGGGTTCTTGGCTG
TGGCTGTAAATGGAGACTCCTGCCCAGGCAGCCACCACCCTCTCCTAGGCTGCTCTGCGC
GTGTTCACTGTAGCTTGTGGGACTTAACATTTCTTCAGGAGTTTGTGCTGGTCCCAGTTT
CCCAGCACCTTCCCTGAAATCTTGTTCTGGCGGGGTCCCTAGCTAAAGGGAAGCTGGGAG
CCTGGTCCCGCCTGTTCCGAGCCGCCTACCCCTTTGGTGCTGACCCGCATCCCTTTCAGT
GCATCATCGAGGAGTCGGGAGAGCATATCATCGCGGGCGCCGGCGAGCTGCACCTGGAGA

TCTGCCTGAAGGACCTGGAGGAGGACCACGCCTGCATCCCCATCAAGGTGAGGCGCCAGT
GACCAGCCTTCCCCACGCCCCACCCCGGACACCTGCCCTCTGCTTTAAAGCTGAGCTGAG
CTAGGCTCTGCAGACGCCTAGACTTGATCTCGGTCTTGGTCTCAGCTTCTTAAGTTCCTC
AAGTTTCTTCTTTGCAGTCTTCCACAGGCATGTGGGGCTGTTTTTGTTGTTGTTGTTGTT
TTTTTTGAGATAGTCTCGCTGTGTCATCCAGGCTGGAGTGCAGTGGCGTGATCTTGGCTC
GCTAGAACCTCCACCTCCCGGGTTCAAGCGATTCTCTTGCCTCACTCAGCTTCCTGAGTA
ACTGGGACTACAGGCGCGTGCTACCACGCCCGGCTAATTTTTTTTTGTATTTTTAGTAGA
GGCGGGGTTTCACTGTGTTAGTAAGTCAAGATGGTCTCGATCTCCTGACCTCGTGATCCC
CCTGCCTCGGCCTCCCAAAGTGCTGGGATTACAGGTGTGAGCCACCATGCCTGGCCCAGG
GCTATTTTTTTTTTTTTTTTTTTTTTTTTTTTGAGACAAGAGTCTTGCTCTGTTGCTTAG
GCTGGAGTGCAATGGCGTGATCTTGGCTCACTGCAAGCTCTGCTTCCCAGGTTCACGCCA
TTCTCCTGCCTCAGCCTCCTGTAGCTGGGACTTCAGGCGCCTGCCATCATGCCCGGCTAA
TTTTTTGCATTTTTAGTAGAGACGGGGTTTCACCGTGTTAGCCAGGATGGTCTGGATCTC
CTGACCTCGTGATCCACCCGCCTCGGCCTCCCAAAGTGCTGGGATTACAGGCGTGAGCCA
CCACGCCCGGCCTCCGGGGCAGTTTTTGAGTTACATCCTGCTGTCTTCTGTCCACAAACA
ACACAAATGCTCTGGAGGGGTTGGGACAGGCCTGCTCCAGACCTCGTTTCTTCCCTGTTA
ATGCTTAAAATTCCACAAGCCCGTGGTTTCTGCGCCGGAGAGCTCAGTGGAGCCCCTGCT
CCTTGTCCTTGTCTGGAGTGAGATGCGCTTGGCACTGCAACACTGACTCCGCTTCGTTCT
GATTGACGTGGCTCTACCAGGCTGTATGAGGTCCGCCTTGCTAGGAAAAGCTTTTAAGGA
TGCGTCTGTGTGTAAGGTCACCTCTTTCTCCAGGCAAGAGTGGGACTTAACCTCTTTTTG
CAGAAATCTGACCCGGTCGTCTCGTACCGCGAGACGGTCAGTGAAGAGTCGAACGTGCTC
TGCCTCTCCAAGTCCCCCAACAAGCACAACCGGCTGTACATGAAGGCGCGGCCCTTCCCC
GACGGCCTGGCCGAGGACATCGATAAAGGCGAGGTGTCCGCCCGTCAGGAGCTCAAGCAG
CGGGCGCGCTACCTGGCCGAGAAGTACGAGTGGGACGTGGCTGAGGCCCGCAAGATCTGG
TGCTTTGGGCCCGACGGCACCGGCCCCAACATCCTCACCGACATCACCAAGGGTGTGCAG
TACCTCAACGAGATCAAGGACAGTGTGGTGGCCGGCTTCCAGTGGGCCACCAAGGAGGTG
AGGCACGGCTCAGCATGTGCAGACCACACCCGTTTCCAGGCTCTAGAGGGACCTCATGGT
CCTGCCTCCCGAGACAGAGACCCTAATGGGGCCAAGGCGGGCAAGGCCCCAGGTCCCCCT
GGTGGAGACCTGCAGGACTTGGCAGGTGGAGGGCAAGCAGCCGAGGTGTGTCCGGCCCTT
GACGGTGGCTCTCCCCCTCCCCCAGGGCGCACTGTGTGAGGAGAACATGCGGGGTGTGCG
CTTCGACGTCCACGACGTCACCCTGCACGCCGACGCCATCCACCGCGGAGGGGGCCAGAT
CATCCCCACAGCACGGCGCTGCCTCTATGCCAGTGTGCTGACCGCCCAGCCACGCCTCAT
GGAGCCCATCTACCTTGTGGAGATCCAGGTGAGGTCTACCCGCCCACCGCTGACCCTGCC
ACCGTCCTGCCCAGCGGCCACTGACAGGTTTTCTTTCCCTTCTGGCAGTGTCCAGAGCAG
GTGGTCGGTGGCATCTACGGGGTTTTGAACAGGAAGCGGGGCCACGTGTTCGAGGAGTCC
CAGGTGGCCGGCACCCCCATGTTTGTGGTCAAGGCCTATCTGCCCGTCAACGAGTCCTTT
GGTGAGTGCCTGCCCGGTGTGGCCTGCAGAGCCTGGCAGGCTGGTTTGGGGGACAGAAGC
CCAGTTAAGCTTAGCAAGGTGTTAAAGGAGGCGTCCTGATGGGAGCAGGTGATGGATGGA
GCAGGTGGTCCAGTTTCTGACAGCTTGTGGACCCCCTAAATCACTGAATTCCCAGGGGAG
GGGCTCTCCTATCCCCAGTGTGAGAAGGGCTCTGGGCCTGGAGCTCTGAAGGCCTACGCC
CTGGGCCGGTAGAGCAGCCGAGCTGTAGCACAGGGTTGTCCCAAACGAGCAGCGGCATGA
GGCCCATGAGTGGCCTGCTAGGCCCTTCGTGAAGTGCTGGGCACCAGGCCGAGTGTCTGG
TCTGCAGGGTGACTCAGGCTGAGGAACTAGCCTGAGCTCCTGACAGGACTTTCCTTCTGC
CCTGCCACCTTCTCGATGGCCCAGTGAGCCTCTCGCTTCCCTCTGCAGGCTTCACCGCTG

ACCTGAGGTCCAACACGGGCGGCCAGGCGTTCCCCCAGTGTGTGTTTGACCACTGGCAGA
TCCTGCCCGGAGACCCCTTCGACAACAGCAGCCGCCCCAGCCAGGTGGTGGCGGAGACCC
GCAAGCGCAAGGGCCTGAAAGAAGGCATCCCTGCCCTGGACAACTTCCTGGACAAATTGT
AGGCGGCCCTTCCTGCAGCGCCTGCCGCCCCGGGGACTCGCAGCACCCACAGCACCACGT
CCTCGAATTCTCAGACGACACCTGGAGACTGTCCCGACACAGCGACGCTCCCCTGAGAGG
TTTCTGGGGCCCGCTGCGTGCCATCACTCAACCATAACACTTGATGCCGTTTCTTTCAAT
ATTTATTTCCAGAGTCCGGAGGCAGCAGACACGCCCTCTTAGTAGGGACTTAATGGGCCG
GTCGGGGAGGGGGAGGCGGGATGGGACACCCAACACTTTTTCCATTTCTTCAGAGGGAAA
CTCAGATGTCCAAACTAATTTTAACAAACGCATTAAGAGGTTTATTTGGGTACATGGCCC
GCAGTGGCTTTTGCCCCAGAAAGGGGAAAGGAACACGCGGGTAGATGATTTCTAGCAGGC
AGGAAGTCCTGTGCGGTGTCACCATGAGCACCTCCAGCTGTACTAGTGCCATTGGAATAA
TAAATTTGATAAGGTGGTGACTCTGTTCTGCATTTTTCACGGTGTCTTCGCAGGGGAGCG
GGGCTGCCCAGTACTGGGCTCCCTGGAGCCTAGAAGGGGACCCGGGCCCTAGTTAGGTGC
AGCCTGGGGCTGCCTCAGTGTTAGGTGGAACGTTCTGGAATGGTGGGAATGCCCTACCCC
TGTGTCATTCAGAGAAGCAGCTGCCAGCTGCGCGGGTCTGCTGAGCATTTGAAGTAGGAT
CAGTGCGGCAAGGAATTACGAGATGTCACTTTGAACGCATTTGGATGGCCCTGTGGAGCG
AGGGGCTCTGGATTGAACTTCGCAGGTTTCAGCAACTTTCCGAATTGCTGACGAGCTCAC
AAGTTCTATCTGCCATCAGGATTTTCTGTGGTCACCCCAGTCCTGACTAGTTTATTAGAA
ACCCATTTTTTTTTTTTTTTTTTTTTGAGATGGAGTCTCACCGTTTGCTAGGCTGGAGTGG
AATGGCACCATCTCTGCTCACTGCAACCTCCACCTCCCGGGTTCAAGCAATTCTGCCTCG
GGCTCCTGGGTAGCTGGGATTACATGCGTGTGCTACCACGCCCAGCCAATTTTTATATTT
TTAGTAGAGATGAGGTTTCACCATGTTGGCCTGGATGATCTTGATCACTCGACCTTGTGA
TCCACCCGCCTTGGCCTCCCAAAGCGTTGGGATTGTACCACTGTGCCCGGCCTTGTAAAC
ACTTTTTTAGAGGCAGAGTCTTGCTGTCTCCCAGATGAGTATAGTGGTGCAGTCAGCTCA
CTGCATCCTCCACCTCCTGGACTCTCCTGCCTCAGCCTGCCAGGTAGCTGGGACTCCAGG
CATGTGCCACCATGCCCAGCTAATTTTTATTTATTTTGAGACAGAATTTTGCTTGGCCTG
GTTGGTC

EEF2 with mNeonGreen insertion:
AAGTCTTGGGCTCCTCAGTCGGGAGCTAGAAGTAAGGAGCATAGAAGGAATATAGGGTGA
TACTTGTGCCAGCCACAACTTTTGTTCTTAGCCCAGTGAGGGCATTTCTGGAGAAGCTCC
AAGCCCCAGGGCACAGCACACCCCTGCTCATCTGTGTCCCTGGGCACAGGTGGGTGGCGG
CCGTCGACCCATACCCAGGGAGGAGGGCAGTGATTCTGAAGAGGATGTTTCCTGACAGCC
CCGACGCAGCAGTCAGGGCCTTGTGGAAGCCACCAGTCTGCCCTGGGACCTGGCAGGAGG
GCCACCAACTCCGGGAGGGACCTGCCCTGGCCCTCCAGGGTCCAGCACAACCATCCCTGC
GGCCTGCTGCCGCCTTCTGCTTCTTCCAGAACAATCTTGATGATGGGCCGCTACGTGGAG
CCCATCGAGGATGTGCCTTGTGGGAACATTGTGGGCCTCGTGGGCGTGGACCAGTTCCTG
GTGAAGACGGGCACCATCACCACCTTCGAGCACGCGCACAACATGCGGGTGATGAAGTTC
AGCGTCAGCCCTGTTGTCAGAGTGGCCGTGGAGGCCAAGAACCCGGCTGACCTGCCCAAG
CTGGTGGAGGGGCTGAAGCGGCTGGCCAAGTCCGACCCCATGGTGCAGGTGGGCACGGAG
TGCACCTGCTGGGAGCAGACACCCTGAGGGGGGACGGCTTGTGTCCTGAGTTGTGGCTGT
GTGGGTTGCTGTGGTTGGTGGATTGGGGTGACTGGTCATGAAAGGAGGGGTTCTTGGCTG
TGGCTGTAAATGGAGACTCCTGCCCAGGCAGCCACCACCCTCTCCTAGGCTGCTCTGCGC
GTGTTCACTGTAGCTTGTGGGACTTAACATTTCTTCAGGAGTTTGTGCTGGTCCCAGTTT

CCCAGCACCTTCCCTGAAATCTTGTTCTGGCGGGGTCCCTAGCTAAAGGGAAGCTGGGAG
CCTGGTCCCGCCTGTTCCGAGCCGCCTACCCCTTTGGTGCTGACCCGCATCCCTTTCAGT
GCATCATCGAGGAGTCGGGAGAGCATATCATCGCGGGCGCCGGCGAGCTGCACCTGGAGA
TCTGCCTGAAGGACCTGGAGGAGGACCACGCCTGCATCCCCATCAAGGTGAGGCGCCAGT
GACCAGCCTTCCCCACGCCCCACCCCGGACACCTGCCCTCTGCTTTAAAGCTGAGCTGAG
CTAGGCTCTGCAGACGCCTAGACTTGATCTCGGTCTTGGTCTCAGCTTCTTAAGTTCCTC
AAGTTTCTTCTTTGCAGTCTTCCACAGGCATGTGGGGCTGTTTTTGTTGTTGTTGTT
TTTTTTGAGATAGTCTCGCTGTGTCATCCAGGCTGGAGTGCAGTGGCGTGATCTTGGCTC
GCTAGAACCTCCACCTCCCGGGTTCAAGCGATTCTCTTGCCTCACTCAGCTTCCTGAGTA
ACTGGGACTACAGGCGCGTGCTACCACGCCCGGCTAATTTTTTTTTGTATTTTTAGTAGA
GGCGGGGTTTCACTGTGTTAGTAAGTCAAGATGGTCTCGATCTCCTGACCTCGTGATCCC
CCTGCCTCGGCCTCCCAAAGTGCTGGGATTACAGGTGTGAGCCACCATGCCTGGCCCAGG
GCTATTTTTTTTTTTTTTTTTTTTTTTTTTTTGAGACAAGAGTCTTGCTCTGTTGCTTAG
GCTGGAGTGCAATGGCGTGATCTTGGCTCACTGCAAGCTCTGCTTCCCAGGTTCACGCCA
TTCTCCTGCCTCAGCCTCCTGTAGCTGGGACTTCAGGCGCCTGCCATCATGCCCGGCTAA
TTTTTTGCATTTTTAGTAGAGACGGGGTTTCACCGTGTTAGCCAGGATGGTCTGGATCTC
CTGACCTCGTGATCCACCCGCCTCGGCCTCCCAAAGTGCTGGGATTACAGGCGTGAGCCA
CCACGCCCGGCCTCCGGGGCAGTTTTTGAGTTACATCCTGCTGTCTTCTGTCCACAAACA
ACACAAATGCTCTGGAGGGGTTGGGACAGGCCTGCTCCAGACCTCGTTTCTTCCCTGTTA
ATGCTTAAAATTCCACAAGCCCGTGGTTTCTGCGCCGGAGAGCTCAGTGGAGCCCCTGCT
CCTTGTCCTTGTCTGGAGTGAGATGCGCTTGGCACTGCAACACTGACTCCGCTTCGTTCT
GATTGACGTGGCTCTACCAGGCTGTATGAGGTCCGCCTTGCTAGGAAAAGCTTTTAAGGA
TGCGTCTGTGTGTAAGGTCACCTCTTTCTCCAGGCAAGAGTGGGACTTAACCTCTTTTTG
CAGAAATCTGACCCGGTCGTCTCGTACCGCGAGACGGTCAGTGAAGAGTCGAACGTGCTC
TGCCTCTCCAAGTCCCCCAACAAGCACAACCGGCTGTACATGAAGGCGCGGCCCTTCCCC
GACGGCCTGGCCGAGGACATCGATAAAGGCGAGGTGTCCGCCCGTCAGGAGCTCAAGCAG
CGGGCGCGCTACCTGGCCGAGAAGTACGAGTGGGACGTGGCTGAGGCCCGCAAGATCTGG
TGCTTTGGGCCCGACGGCACCGGCCCCAACATCCTCACCGACATCACCAAGGGTGTGCAG
TACCTCAACGAGATCAAGGACAGTGTGGTGGCCGGCTTCCAGTGGGCCACCAAGGAGGTG
AGGCACGGCTCAGCATGTGCAGACCACACCCGTTTCCAGGCTCTAGAGGGACCTCATGGT
CCTGCCTCCCGAGACAGAGACCCTAATGGGGCCAAGGCGGGCAAGGCCCCAGGTCCCCCT
GGTGGAGACCTGCAGGACTTGGCAGGTGGAGGGCAAGCAGCCGAGGTGTGTCCGGCCCTT
GACGGTGGCTCTCCCCCTCCCCCAGGGCGCACTGTGTGAGGAGAACATGCGGGGTGTGCG
CTTCGACGTCCACGACGTCACCCTGCACGCCGACGCCATCCACCGCGGAGGGGGCCAGAT
CATCCCCACAGCACGGCGCTGCCTCTATGCCAGTGTGCTGACCGCCCAGCCACGCCTCAT
GGAGCCCATCTACCTTGTGGAGATCCAGGTGAGGTCTACCCGCCCACCGCTGACCCTGCC
ACCGTCCTGCCCAGCGGCCACTGACAGGTTTTCTTTCCCTTCTGGCAGTGTCCAGAGCAG
GTGGTCGGTGGCATCTACGGGGTTTTGAACAGGAAGCGGGGCCACGTGTTCGAGGAGTCC
CAGGTGGCCGGCACCCCCATGTTTGTGGTCAAGGCCTATCTGCCCGTCAACGAGTCCTTT
GGTGAGTGCCTGCCCGGTGTGGCCTGCAGAGCCTGGCAGGCTGGTTTGGGGGACAGAAGC
CCAGTTAAGCTTAGCAAGGTGTTAAAGGAGGCGTCCTGATGGGAGCAGGTGATGGATGGA
GCAGGTGGTCCAGTTTCTGACAGCTTGTGGACCCCCTAAATCACTGAATTCCCAGGGGAG
GGGCTCTCCTATCCCCAGTGTGAGAAGGGCTCTGGGCCTGGAGCTCTGAAGGCCTACGCC
CTGGGCCGGTAGAGCAGCCGAGCTGTAGCACAGGGTTGTCCCAAACGAGCAGCGGCATGA

GGCCCATGAGTGGCCTGCTAGGCCCTTCGTGAAGTGCTGGGCACCAGGCCGAGTGTCTGG
TCTGCAGGGTGACTCAGGCTGAGGAACTAGCCTGAGCTCCTGACAGGACTTTCCTTCTGC
CCTGCCACCTTCTCGATGGCCCAGTGAGCCTCTCGCTTCCCTCTGCAGGCTTCACCGCTG
ACCTGAGGTCCAACACGGGCGGCCAGGCGTTCCCCCAGTGTGTGTTTGACCACTGGCAGA
TCCTGCCCGGAGACCCCTTCGACAACAGCAGCCGCCCCAGCCAGGTGGTGGCGGAGACCC
GCAAGCGCAAGGGCCTGAAAGAAGGCATCCCTGCCCTGGACAACTTCCTGGACAAATTGC
AGTGTACTAATTATGCTCTCTTGAAATTGGCTGGAGATGTTGAGAGCAACCCAGGTCCCA
TGGTGAGCAAGGGCGAGGAGGATAACATGGCCTCTCTCCCAGCGACACATGAGTTACACA
TCTTTGGCTCCATCAACGGTGTGGACTTTGACATGGTGGGTCAGGGCACCGGCAATCCAA
ATGATGGTTATGAGGAGTTAAACCTGAAGTCCACCAAGGGTGACCTCCAGTTCTCCCCCT
GGATTCTGGTCCCTCATATCGGGTATGGCTTCCATCAGTACCTGCCCTACCCTGACGGGA
TGTCGCCTTTCCAGGCCGCCATGGTAGATGGCTCCGGATACCAAGTCCATCGCACAATGC
AGTTTGAAGATGGTGCCTCCCTTACTGTTAACTACCGCTACACCTACGAGGGAAGCCACA
TCAAAGGAGAGGCCCAGGTGAAGGGGACTGGTTTCCCTGCTGACGGTCCTGTGATGACCA
ACTCGCTGACCGCTGCGGACTGGTGCAGGTCGAAGAAGACTTACCCCAACGACAAAACCA
TCATCAGTACCTTTAAGTGGAGTTACACCACTGGAAATGGCAAGCGCTACCGGAGCACTG
CGCGGACCACCTACACCTTTGCCAAGCCAATGGCGGCTAACTATCTGAAGAACCAGCCGA
TGTACGTGTTCCGTAAGACGGAGCTCAAGCACTCCAAGACCGAGCTCAACTTCAAGGAGT
GGCAAAAGGCCTTTACCGATGTGATGGGCATGGACGAGCTGTACAAGTAACGCGTGCGGC
CCTTCCTGCAGCGCCTGCCGCCCCGGGGACTCGCAGCACCCACAGCACCACGTCCTCGAA
TTCTCAGACGACACCTGGAGACTGTCCCGACACAGCGACGCTCCCCTGAGAGGTTTCTGG
GGCCCGCTGCGTGCCATCACTCAACCATAACACTTGATGCCGTTTCTTTCAATATTTATT
TCCAGAGTCCGGAGGCAGCAGACACGCCCTCTTAGTAGGGACTTAATGGGCCGGTCGGGG
AGGGGGAGGCGGGATGGGACACCCAACACTTTTTCCATTTCTTCAGAGGGAAACTCAGAT
GTCCAAACTAATTTTAACAAACGCATTAAGAGGTTTATTTGGGTACATGGCCCGCAGTGG
CTTTTGCCCCAGAAAGGGGAAAGGAACACGCGGGTAGATGATTTCTAGCAGGCAGGAAGT
CCTGTGCGGTGTCACCATGAGCACCTCCAGCTGTACTAGTGCCATTGGAATAATAAATTT
GATAAGGTGGTGACTCTGTTCTGCATTTTTCACGGTGTCTTCGCAGGGGAGCGGGGCTGC
CCAGTACTGGGCTCCCTGGAGCCTAGAAGGGGACCCGGGCCCTAGTTAGGTGCAGCCTGG
GGCTGCCTCAGTGTTAGGTGGAACGTTCTGGAATGGTGGGAATGCCCTACCCCTGTGTCA
TTCAGAGAAGCAGCTGCCAGCTGCGCGGGTCTGCTGAGCATTTGAAGTAGGATCAGTGCG
GCAAGGAATTACGAGATGTCACTTTGAACGCATTTGGATGGCCCTGTGGAGCGAGGGGCT
CTGGATTGAACTTCGCAGGTTTCAGCAACTTTCCGAATTGCTGACGAGCTCACAAGTTCT
ATCTGCCATCAGGATTTTCTGTGGTCACCCCAGTCCTGACTAGTTTATTAGAAACCCATT
TTTTTTTTTTTTTTTTTTGAGATGGAGTCTCACCGTTTGCTAGGCTGGAGTGGAATGGCA
CCATCTCTGCTCACTGCAACCTCCACCTCCCGGGTTCAAGCAATTCTGCCTCGGGCTCCT
GGGTAGCTGGGATTACATGCGTGTGCTACCACGCCCAGCCAATTTTTATATTTTTAGTAG
AGATGAGGTTTCACCATGTTGGCCTGGATGATCTTGATCACTCGACCTTGTGATCCACCC
GCCTTGGCCTCCCAAAGCGTTGGGATTGTACCACTGTGCCCGGCCTTGTAAACACTTTTT
TAGAGGCAGAGTCTTGCTGTCTCCCAGATGAGTATAGTGGTGCAGTCAGCTCACTGCATC
CTCCACCTCCTGGACTCTCCTGCCTCAGCCTGCCAGGTAGCTGGGACTCCAGGCATGTGC
CACCATGCCCAGCTAATTTTTATTTATTTTGAGACAGAATTTTGCTTGGCCTGGTTGGTC

AAVS1:

TGCAAACAGGAAGTGAACGGGGAAGGGAGGGGGCTTCTCATCTGGGTGCGGGAACCCCAC
ATGGTACCTGTTAGACACGGCAAAACCCCCGTCACCACCCACAGGTGGCGCTTCCAGTGC
TCAGACTAGGGAAGAGGTTCCAGCCCCTCCTCCTTCAGAGCCAGGAGTCCTGGCCCCCAG
CCCCTCCTGCCTTAAACCCAGCCAGGTCCTTCCAAGGGTCAAGCTCGGAAACCACCCCAG
CAGATACTCTGCAGGAACGAAGCCGTGGGCCCAGGGCTATGCAGGGTGGAGGAAGGCCAC
CCTGTGCTGGGACAGACTCAGGGGCCTGGGCGGGACTCCCAGAGGGGTGAGACAGCTGCA
CACCTGTGTGCCTGGGCCCCAGGCTGTCACACTCCAGTTCACTGAGGCCCCCTCTGCACG
GGGCCCTGCAGCCAGGGGCTGACACGGGCCACCGTTTCTCATTCTTCCCTTAGGGGTCCA
AAACTTGGGGGGACAAAAGCCGAAGTCCAGGGGGTCGGAGGAGGGACTTGCCCCAGGCCT
TGTGGACACTGGGTGGGCTCCGGGACCTGAACTGGAGCTGAGGAAGGAGTGAAGCTAAAC
TCCTAGATCCACGGGATAAATTACCCCCCAAGTCCCTCACCTCTCCAAAGCTGCCCATCT
GGAGGAGGCGGGAGGGAGCTACGAGGGCCAAGAGCATGAGGTCATGGAAACTCGGGCTGT
GAAGGGGCCGCACGTGCCCTGGGAACGGGATGAACTCGGCTCGTTTATTTCCACCCAGTT
GTCATGGCGATAGGGGAGGGGGGCAAGGAGAGCAATGGGCCTTTCCCTTTCAAGGACCTG
CCCAGTACAGGCATCCCTGTGAAAGATGCCTGAGGCCTGGGCACCAGGGACTCCAGAGTC
CAGGCCCAACCCCTCCCCATTCAACCCAGGAGGCCAGGCCCCAGCCCTTCCGCCCTCAGA
TGAAGGAGTCCAGGCCCCCAGCCTCTCCCCATTCAGACCCAGGGGTCCAGGCCCAGCCCC
GCCTCCCTAAGACCCAGAAGTCCAGGCCCCCAGCCCCTCCTCCCTCAGACCCACGAGTCC
AGGCCCCAGCCCCTCCTCCCTCGGACCCAGGAGTCCAGGCCCCCAGTCCCTCCACCCTCA
GACCCAGGAGTCCAGGCCCCAGCCCCTCCTCCCTCGGACCCAGGAGTCCAGGCCCCAGCC
CCTCCTCTCTCAAACCCAGGAGCCCAGGCCCCCAGCTCTTCTCTGTTCAGCCCTAAGAAT
CCTGGCTCCAGCCCCTCCTACTCTAGCCCCCAACCCCCTAGCCACTAAGGCAATTGGGGT
GCAGGAATGGGGGCAGGGTACCAGCCTCACCAAGTGGTTGATAAACCCACGTGGGGTACC
CTAAGAACTTGGGAACAGCCACAGCAGGGGGGCGATGCTTGGGGACCTGCCTGGAGAAGG
ATGCAGGACGAGAAACACAGCCCCAGGTGGAGAAACTGGCCGGGAATCAAGAGTCACCCA
GAGACAGTGACCAACCATCCCTGTTTTCCTAGGACTGAGGGTTTCAGTGCTAAAACTAGG
CTGTCCTGGGCAAACAGCATAAGCTGGTCACCCCACACCCAGACCTGACCCAAACCCAGC
TCCCCTGCTTCTTGGCCACGTAACCTGAGAAGGGAATCCCTCCTCTCTGAACCCCAGCCC
ACCCCAATGCTCCAGGCCTCCTGGGATACCCCGAAGAGTGAGTTTGCCAAGCAGTCACCC
CACAGTTGGAGGAGAATCCACCCAAAAGGCAGCCTGGTAGACAGGGCTGGGGTGGCCTCT
CGTGGGGTCCAGGCCAAGTAGGTGGCCTGGGGCCTCTGGGGGATGCAGGGGAAGGGGGAT
GCAGGGGAACGGGGATGCAGGGGAACGGGGCTCAGTCTGAAGAGCAGAGCCAGGAACCCC
TGTAGGGAAGGGGCAGGAGAGCCAGGGGCATGAGATGGTGGACGAGGAAGGGGGACAGGG
AAGCCTGAGCGCCTCTCCTGGGCTTGCCAAGGACTCAAACCCAGAAGCCCAGAGCAGGGC
CTTAGGGAAGCGGGACCCTGCTCTGGGCGGAGGAATATGTCCCAGATAGCACTGGGGACT
CTTTAAGGAAAGAAGGATGGAGAAAGAGAAAGGGAGTAGAGGCGGCCACGACCTGGTGAA
CACCTAGGACGCACCATTCTCACAAAGGGAGTTTTCCACACGGACACCCCCCTCCTCACC
ACAGCCCTGCCAGGACGGGGCTGGCTACTGGCCTTATCTCACAGGTAAAACTGACGCACG
GAGGAACAATATAAATTGGGGACTAGAAAGGTGAAGAGCCAAAGTTAGAACTCAGGACCA
ACTTATTCTGATTTTGTTTTTCCAAACTGCTTCTCCTCTTGGGAAGTGTAAGGAAGCTGC
AGCACCAGGATCAGTGAAACGCACCAGACGGCCGCGTCAGAGCAGCTCAGGTTCTGGGAG
AGGGTAGCGCAGGGTGGCCACTGAGAACCGGGCAGGTCACGCATCCCCCCCTTCCCTCCC
ACCCCCTGCCAAGCTCTCCCTCCCAGGATCCTCTCTGGCTCCATCGTAAGCAAACCTTAG
AGGTTCTGGCAAGGAGAGAGATGGCTCCAGGAAATGGGGGTGTGTCACCAGATAAGGAAT

CTGCCTAACAGGAGGTGGGGGTTAGACCCAATATCAGGAGACTAGGAAGGAGGAGGCCTA
AGGATGGGGCTTTTCTGTCACCAATCCTGTCCCTAGTGGCCCCACTGTGGGGTGGAGGGG
ACAGATAAAAGTACCCAGAACCAGAGCCACATTAACCGGCCCTGGGAATATAAGGTGGTC
CCAGCTCGGGGACACAGGATCCCTGGAGGCAGCAAACATGCTGTCCTGAAGTGGACATAG
GGGCCCGGGTTGGAGGAAGAAGACTAGCTGAGCTCTCGGACCCCTGGAAGATGCCATGAC
AGGGGGCTGGAAGAGCTAGCACAGACTAGAGAGGTAAGGGGGGTAGGGGAGCTGCCCAAA
TGAAAGGAGTGAGAGGTGACCCGAATCCACAGGAGAACGGGGTGTCCAGGCAAAGAAAGC
AAGAGGATGGAGAGGTGGCTAAAGCCAGGGAGACGGGGTACTTTGGGGTTGTCCAGAAAA
ACGGTGATGATGCAGGCCTACAAGAAGGGGAGGCGGGACGCAAGGGAGACATCCGTCGGA
GAAGGCCATCCTAAGAAACGAGAGATGGCACAGGCCCCAGAAGGAGAAGGAAAAGGGAAC
CCAGCGAGTGAAGACGGCATGGGGTTGGGTGAGGGAGGAGAGATGCCCGGAGAGGACCCA
GACACGGGGAGGATCCGCTCAGAGGACATCACGTGGTGCAGCGCCGAGAAGGAAGTGCTC
CGGAAAGAGCATCCTTGGGCAGCAACACAGCAGAGAGCAAGGGGAAGAGGGAGTGGAGGA
AGACGGAACCTGAAGGAGGCGGCAGGGAAGGATCTGGGCCAGCCGTAGAGGTGACCCAGG
CCACAAGCTGCAGACAGAAAGCGGCACAGGCCCAGGGGAGAGAATGCAGGTCAGAGAAAG
CAGGACCTGCCTGGGAAGGGGAAACAGTGGGCCAGAGGCGGCGCAGAAGCCAGTAGAGCT
CAAAGTGGTCCGGACTCAGGAGAGAGACGGCAGCGTTAGAGGGCAGAGTTCCGGCGGCAC
AGCAAGGGCACTCGGGGGCGAGAGGAGGGCAGCGCAAAGTGACAATGGCCAGGGCCAGGC
AGATAGACCAGACTGAGCTATGGGAGCTGGCTCAGGTTCAGGAGAGGGCAGGGCAGGGAA
GGAGACAAAGTCCAGGACCGGCTGGAGGGGCTCAACATCGGAAGAGGGGAAGTCGAGGGA
GGGATGGTAAGGAGGACTGCATGGGTCAGCACAGGCTGCCAAAGCCAGGGCCAGTTAAAG
CGACTCCAATGCGGAAGAGAGTAGGTCG

BCL11A-1:
GTGTGGTGTTCGGAGTCCTAAGAGCCCCCACTAGCTCAGAAATGGACTTAGTTGACCTCC
CCCATTAGCAGCATGGAGAGTCAAGGAGATGACTTCTACCTTGCCAAAGGCCTTGGGAAG
AAAGACAGCATCAAGGTCTCACACAACACTCCAGGGAGGCAGCTGCTGCCCAGTGCTGTG
GACAGCAAAGCTTCAGTGCAGGAAATTAAGATTCCCCCTGCCTCCCCCTCCCCCATCCTC
ATCAGCTTGGCCATGGCAGGGCTGGGGGATCAGAGGTGAACAGGAAGCAGAAGGACCCCT
GGGGGAGACAGGGCCTCCAGTGGGACCAGAGCTGAGTGGCCTCAGGCAGTGGCGGAAGCT
GATTAAAGGAAGGTACGGGGAGTGGAGGGGAAGTGGACAAAAGACAGGACAGCCATCTTA
GACAACAATGCAAGGGGGAGAAACTGAAGAAAACAGAACAGAGACCACTACTGGCAATAA
ACAGAGAGAAAGTGAAGCCCCATGGGTGAGGCACACCTACATTACTTAAGAAACCTGAGC
ACATTCTTACGCCTAGGGCAATAAATACATCCTTGAGCTACACAGGCTAAGCAAGAGTGA
GAGAGGGTGATGCTGACAGGCCACATGGGAGAGTGGGAAGACGTGGGCTGGGAGCTGGGA
GTTTGGCTTCTCATCTGTGCATGGCCTCTAAACTGGGCAGTGACCATGGCCTGGTCACCT
CCCCACTCTGGACCTGGGTTGCCCCTCTGTAAACAAGGAGGTTGTAATAAATTATCTCCA
ATACCCTAATGTCTTATAAATCTTATGCAATTTTTGCCAAGATGGGAGTATGGGGAGAGA
AGAGTGGAAACGGCCCAGAGCTCAGTGAGATGAGATATCAAAGGGGACGAAAAGTGTTCA
TTCCATCTCCCTAATCTCCAATTGGCAAAGCCAGACTTGGGGCAATACAGACTGGTTCTG
TGATGACAAATAACTCCTAGCTCATTCCTAATGATTTATCACCAAATGTTCTTTCTTCAG
CTGGAATTTAAAATATGGACTCATCCGTAAAATAGGAATAATAATAGTATATGCTTCATA
GGGTTTGTATGAAAATAAAATGAGTGCGTATTTGTAAAGTTCCTAGAGCAGAGTAAGTGC
TCCGAGCTTGTGAACTAAAATGCTGCCTCCTGGTATTTATTAGTTACACCTCAGCAGAAA

CAAAGTTATCAGGCCCTTTCCCCAATTCCTAGTTTGGGTCAGAAGAAAAGGGAAAAGGGA
GAGGAAAAAGGAAAAGAATATGACGTCAGGGGGAGGCAAGTCAGTTGGGAACACAGATCC
TAACACAGTAGCTGGTACCTGATAGGTGCCTATATGTGATGGATGGGTGGACAGCCCGAC
AGATGAAAAATGGACAATTATGAGGAGGGGAGAGTGCAGACAGGGGAAGCTTCACCTCCT
TTACAATTTTGGGAGTCCACACGGCATGGCATACAAATTATTTCATTCCCATTGAGAAAT
AAAATCCAATTCTCCATCACCAAGAGAGCCTTCCGAAAGAGGCCCCCCTGGGCAAACGGC
CACCGATGGAGAGGTCTGCCAGTCCTCTTCTACCCCACCCACGCCCCCACCCTAATCAGA
GGCCAAACCCTTCCTGGAGCCTGTGATAAAAGCAACTGTTAGCTTGCACTAGACTAGCTT
CAAAGTTGTATTGACCCTGGTGTGTTATGTCTAAGAGTAGATGCCATATCTCTTTTCTGG
CCTATGTTATTACCTGTATGGACTTTGCACTGGAATCAGCTATCTGCTCTTACTTATGCA
CACCTGGGGCATAGAGCCAGCCCTGTATCGCTTTTCAGCCATCTCACTACAGATAACTCC
CAAGTCCTGTCTAGCTGCCTTCCTTATCACAGGAATAGCACCCAAGGTCCATCAGTACCT
CAGAGTAGAACCCCCTATAAACTAGTCTGGTTTGCCCATGGGGCACAGTCAGGCTGTTTT
CCAGGGTGGGGTGCAGACATTCTCTGCCTGTTGTGATGCTTACATATAACGTCATAACAG
ACACACGTATGTGTTGTGATCCCTGTGGTTTGAGAGTTTGGAGCTTCCCTAAAAGTCAAA
ATATTCTCAATGGGCCCTCAATCAGCACATACACACAAAAGGTACCTGGAAAACTGTAAT
TCTTTTCCTGCTCAAAGACAGGCAATTCAATACCCCTTCCCCCAACCAAAAACCCTTGCC
ACCATGGGAGCCTGGGGCAGAGAAGGCACAGTGAAGTCAAACTGTAATTCCAGGCTCTAA
ATGGTGCTGTCATTTTTCTGAGAGTCTCTAAATTACAAGGGTGTTTTCACTATTCTTAGC
TATTTTTTAAAACACCTAAGAAACATACTGCAGCTCTGGAAAAGAGAACAAACAAACCAA
AGAGAAGGGATCCAGAGGTCACCCTCATATGTGAAAAGTCAATTGATAATGAAGGCTTTA
GGATAACCGGAGGGGAGATGATTGAAAGCAATGCACCTGTGCAGGAAATGGATTACGGAA
ACAGGGAATTGTTCATGAAATCCCAGAAAACCAGAACCGGGAAAGTTCTGGAAGTCGGAA
AAACAAATCATGACTTAAGCAATGGAAGTCCAATACACGTTTACAGAATGCCTTGTCCCA
CGAGGCAACACAGGCTACCACAGATGGGGGACAGGGTGGGAGTGGACCATCCCAGTGGTG
TTACTGAGGGGCAAAGGGATAGCCCTATGAGGCAAGTGTCCAGGGCAGAACTGGAGCTTT
GTGAAACCATTTCCCAGGCAGAGACAGAGCACTAGGCTGGTGCTGCCAGTCTGACAATAA
GTCTGCCATTGTCCTCTGGTCAGCTCTGGACACACAGCAAAAGTGAGTTCAGAGTAGCCT
GAAGCAGGAAAGAGGGAAGAGAGGAGGATAACACCTATCTTCCACTTTGCTGCAGGTTCA
AGGCAAGGATTTGAGACAGTTACCCCTTCTGGAAGAGCCTGGTGAGTACATCTCTCCTGC
CTTGTACAACCCTCTCTCCTCACCGACTTTCTCTCCCAGCAGCCAGCAGGGGCCTGGGCC
ATTTATGGAATGCAAGCCCTGACCACACAGACTTACTTACATGCCAGGACAGCCACCAGG
TAGCCTTTCCCACTCTAGGTTCCACTGTGAGTGCTCTCTCTCTCTCTCTCTCTCACTATG
CTCCCAAGAGGAGTCTTACATCAACCCCTTCCTCAAATCTCCCTCACTGGATGTCACAGT
CATAGGCCTGAAAAGCAGCATGCAAACTGAATTTTTGTAAAGCAGGACCCATTTCCCCAT
GGACAGTCATAAGAGATGAGTGAACACAATGTAGCACTTAATTTCTGTCTTCACGATTAC
TTCACGATAAATCTGGATTCCAAAGGGACTATAAGCTCTCACATGGAAGGAAGCAAGATC
TCTACTCCTCCCCCAGTGTTGAGTGGACAGGGAGTACACCGCAGACACCTGTTGGCCAAC
CAATTCTAATTCCCTTTAGCTAGCATCCCCTAAGCTAGAGCTAGAGCTAGAGCTATTTCC
TTGCAGCCTTCCTTTTCTCTAGCAAAGTCCTTCCATGCAGTAGCTAATGACCTGTAAACA
CTTAATGAGCTAGAGAAACATTCCATTGAAAGGAATACCACTGTGCATCCTTTTGTAAAG
AGGGGGGAAAATCTTTTGTAAAACGAAGCATCGCCTTTAACTGCTCTGTTTGATCAAGTC
AGATTTTTCAGAATATGAATAGCTAGTATTCAAGCATATATGAACTGTCTTTAAGTTAAT
CAATCCCTAGAAACTAGCCCTCAGGTTAGCAGGCCAAGGATATATGAGAGTGCTTTGAAG

TCTAGACTTAAACTGCCGCTCCT

BCL11A-2:
GCTGTGCTTTCTTCTATGATTCCTCAGAAACTCTTGTCGACTAGAGGAACAAGAACCTTA
CTCTAAAACCCAATTTTAGGGGAGGAGAGAAAATTTTTAAAAGACTTTAAATTAATTACT
TGAAGGCCAGAGGCATTTGCTTATGCCTATAATCTCAGAAATTTGGAAGGCCACAGAGGG
AAGATTGCTTGAAGCTAGGAGTTCAAGACCAGCCTGGGCAACATAGCAAAACCCTGTCTC
TACAAAAAGCTTAATAAATAAAATAAAATAATTGCTGAGATTTAGCTATCTTTTCAAAGA
AAATATAAAATTTGGCCCAGTTTTTTATCTATCAAACCTAGACCTAAAATCATCTTGTCT
CATTCCCTCTGTCAACATCTGCTGAGCTCTCATAATACATAAAACATCATAGATAAAGGA
GTCACAGGATTAGACCTACCGTTCCATACCAGTGTGTTACAGCCTGTCATGAAGCAGACT
CAGAGAATTTGGCTACTACTTAGTACTAACATTTAATTTAGTTTTAAAATGTCTGCGATA
GAGCAGATTCAGGATTATGCCTGTAAGATGACATTCCAACTCACCTGCATTTAAATATGA
ATAAAGAGTGAGCAGAAGGGACCAGGTAAAAATGGCACATGGGAGGGCATCGCACATGTG
AATTTTAGCTTCCCACACGCAAAGGCATGGGGGGACAAGGCAGGAAGCATTGCTCTAGAT
CTGAGAATCACCACACACCAGAGAGGGCATGGGAGGTGACACACAAACCCAGCAGAGAAC
GTTGAGAAAGAGTTTTAAGAGCCTACTCATCATCAGAGTTCACTTAAAAGTCCATCCAGA
ATCAGAACGTGGGATGGGGTGCCCCAAGGCATTCCTGGGGGCCGGGCGTGGATTTTAAGG
ACTCATCAATTACTGTTCAAGTTCAGGTCAAGCTGGATGGACACCATCCACATGGTGTTG
CTCGATGGGCAAAGATCAAGAGGCAGTCGTTCACCACCAGCCGTATTTCCTGGGGCTGCT
TGTTACTGACATTCTGTCATTCAAGGTGGGTCCTGAAGCCACAGTAAGCACCAAATCTGC
TCCCTACAATCCACAGCAAAAGGAGAGTTCCTTCTTTTAAAGAGCGATGGCACACATCTC
TCAACTCTTGAGAGAAAATGGACAGAAGTGGGATTTTTCAATCCTGATACCAGCAGGAAA
CAGGGAGTCTCTGGTAGGTACTACCAAACCAAGAAGCTTCTTTTCTCCCAAGTACACCAG
GAACCCCTTTCAAATACAGGCGCTACAAAAAAAGAAATAGGTGTGAAAGCGGGGCAAGAA
AGGGAACCTGAGGACTATGCATGCTCTGTCTATGGGTATCAACACCTTTAAGGCATTTTG
CGGTCTTTTTGATTCTATAACCTCCATATTGAGAGACATAAAACACAGAACATTACACCT
GTCAGGTGCTGGGCCCTGAACTATCTCCAGGCAGCTGAAAGTCAGTACATCAGCTTTTTA
ATGATGTGAAAGTTAAAGATTAAAAACATGTTGGAACGCTTACTTTATGCAGTGCCAAAA
ATGTGTCTATAACCTGTAAGGAGAGATGGGTGTGGAACATCGTAGGAAAAAGGTGACTGC
ACTCATGCTGCAGAGAAAAAATAAGGTCCTCACTTGCCTGCCTGCCTGCCTGCCTGCCTG
CCTGCCTGCCTGCCTGCCTTCTTTCCTTACAAGAGAAATCGCTCCTGAAATCTGACGTGC
TTTGCAACAGCAGTAAGTTGACCACTGGAGAAAGTGAGGCTGGAACCACTGGAATCTTGT
AGATTTCTGAGGCCAGTTCTGTTCTGACACATGCAGCTGGGAGCCACTGGTTTAGGAATT
CACGTGGGCATGGGACTGTGCTTATTCAGAAAGGGCTCCCAGTCACATCCTGTATTCTCC
TAAATTACTCCAATGACAAAGAAAACTCAGTGAAGACAAAGAGATCGTTTTCATAGGCAT
TGTCATTTGCCAATCCATAACTCAGGAATTCATGGCTATGCTTGGATAATTCTCTCTAGA
CTTGGAGTGTTAAAAGTTTGCACCTAGTTATTCTCCCCTCTCTCTGGCAGGAGAATCATG
TCACTGGCAAGCAATTATTGTATCCTCTTCAAATAATTCGGCCTAAATTTTCATTTATGC
CTGTGTATTTATGCACAAATGTGTGTATGATATAGTATTATGGCTTCTTTTTCTGAAAAT
TGAAAAATTCAGAAAGCCAAAGATAATAGAATATACCTTGCGTGGCAAGGTATATTGATT
AGGTAGGAATCAAAAATAATTGCAAAATTATTTATGCAGGAGTATTAAAATTTTCCTCAA
AAGTTCCCAGGCAAATGGGTTCCTCTTTAATTTTCACCCTTACAATAAACTCTTCTGCAG
GTTTTGTTTATTTTGAGGAAACATTTCACTTTTTTTTTCAAGACATGAGGTGTGAATGAT

GCAAAACAGTATTTGGATATGTAAACTGAATTATTTGCATAATGCATTACTAATCAGTGA
GTATAAATTCTGGAGTCTGAGGACCTGAAATCAAGTGCCACCAAATTTTTTAAAATCTAG
ATTATAAAAGTAAAGAGCATCACCCTTCGGTGGGATAATGTACACATTTGTGTACACATT
CGCATTCCGTTTATAAGAAGGAGAAATTATCCTCACAGAGTTTTCTGAGGAAAATTAAAT
AAGTGGCCTTTGATGTAAACTGTTTTGCTCTGACTCAACACTATGAGGCATAAGGTCCTA
AATTCATAGGCACAACCACAGAAGCACCAAGGAGAGTGAAGATATTTGCATCAAAAAGAG
CAGACAGTCCCCCACCCCCACCCCATGTTGGTCGCCTACGGAATCTACAGGAATCTGAAG
GGGCAGCCAGACATTCTGCCTATACCACCCATCAGATCAGCCACCAACCGAACCAAAAAC
CACCCATGCCCTTCTCCAGTGGGTTCCCACCGGTGTTTTCGTGCAAGCTTAGCCCCTTCT
TTGCAGAAAAAAAGATATTGATGTGCCAACAGTAGAAAGAAGAAAAAATTATTCCAGCTC
TGTACACAACCTTTGTCAACCCAGCATGATAAAACCAAGTCTGGGAAACTATTTGTCTAA
ACACTTTTTGGAAAGGGATGAAATGGAGAGGAAAGGAAAAGGAAGTGTAGCCTAGTGGTC
AGCACACACACACACACACACACACACACACACACTCACACACACACACACACACACT
CACACAAATCCTTGAGGGTGCCAGGTTACATTTCTACAACAGCTGCCTTCCATTGTCTAC
TGCCAGTGAAGCCAAACCAGCCTAAAATTTAATTTAATCTGCTGTAAACCCAAGTATCAG
GCACTTAGATGGCAGCTAAAAGCGCCACTCTCAGATAACTGAGTGACACTTGCTAATAAT
AGCCTCTGTGTGAAGCGAGAGAGAGCGGACATTTTGTAAAACCAGAGGGACCCGGGCCTG
GGCCTGGGCCTGGGTCTGGGAATGGTGTGATGTGGTTAGGCCAGTCTCAGCTTCCAGGCA
GCAGATACGGCAGAGACGAGGCCCACCATGGCTCCCCTGTGACGGGCTGCACGAAGCCCA
CCAGTGACCTAGCTTTCAAGTCACACAGCGACAGTCCTGGCTTTTGCTATCTAGAAGAAC
AAAATTCCCCTTTCCAGCTCTGCAATTGCCACTCTGCTCCCTTTCATCTTCCATCTTGTG
CCCCAGTCCTCCCTTAAAGGGAAGGCTGCTTAACCCTTGACGTGTTCTTTGTCCCCTTCT
TTCTGAGAGTGTGGTCAATCTCCTCTCAGGCAAATCATCTTTCTCTAATAGCCAGGGCTC
AAATGGCCAAAGAAAGGTCCCAAAGCCTTGTCTTCATAACACTACCAGCCACGTGAGCAC
GTCCCAGCTGCTTCTTCCTGACCCTCACCACGAGGCACAGAGGCACCTGTGCTGAAACTC
AAGCAAACGCCACATCTGACTCTGCCTCATGCACTGAAAAAGACAAGGGATCTCTGTTCT
TCCAGGAGAATGGGGGACAGTGCACAGTACAGACCCTTAAATGACCCCTTCACAATGAAC
TTCCCAGATACAAGACTTGAGCTCACAGGTACTTTCTTGCCCATAGGACACTTGTCCCCT
TGCTACCTCTGAGCCAAACCCTTGGTGAAGAAACAACACCGACTAAACAAGGCCATACTG
CTCTCCACCAGTCAGGCAATACCTTTATTTTCTGGAAAAATCTCAAATACTACAAAAGTA
GAGAGAATGTTATAAATCAGTCCCCAAGTCCACATCTTCCAGCTCCAACCACCACAGCTC
ACAGTCATTCTTGTCTTCTGTAACCCTATCGCCTCCCTAGCCCATTTTATTATTTAAGTT
GATCCCAGACGTCATATCATTTCATCAGTAAACACCTCTGTATGTATTTTTTAAACATAG
GAACTCGTTTTTTTTCTTTTAAATACAACTGCAAAACCATTATCACACCTAAAAATAATA
AAGTCACAAATAAAGTCCCGTATTACCAAATAGGGGAGTTATTTTAAACTCAGAAATACT
TCTTTCCATCTGGGTCATGTTTTGGAGGATCCCTTCCAGTGAGGTTCAAGACTGAAGCTG
CCAAGAAAAGCTTCAGAGAATCACACTCTCTAAAAAGGGCCTTGTGTAAAGCCACCTTGC
ATGTCAGCCCAAGCTCCTGCCCCTTGCAGTCCTTTGCCCTGTTCAGGGCTGAATGCACCC
TCTGAGAGTCTCTGTGACAGTCCTCCCACATCGACTGCCCCACAGGCCCTGAGTCTCCCT
CACTCCCCACCATGTGGCCCATCACCACATCCGACAAGCAGGTGCACTCCCAGTGTTCAG
AACTGGAATTCATTTGTCTTGGAAGAAAGTGTTCTCCTGGACGGTTCTGCACAGAATTCA
TTCACAGAGCATGTCTTGGATATACAGCTGACTAGATATGTCAGTTATGTTCCAAATGGG
TTTTCACAGGTCAAGGTTCTGCAGGCAAGAGCAAATACTATTTTTAGACAGGATAAGGTC
AAAATGCTTTTAGAGACAGTGAGGTCTACAATAAAACCTATTGATCAGCCTCTGGAACTA

GTGCTCCAGAGAGACACAGAGATTGAAACAAAAATTGCCTGGCCGTTTTCTCTGTAGACT
CTGTGGGTAAAAGCTTGTTGGACTTAATATCTTTCAGGATATAAAATAAGTAAGAAGTAA
CTGTGTACAGATTACTGAAGTCCATTTAACCAGATGCAGAGGCAGGTCACAGAAAATACA
CTCATGGCAGAACCGTAAAGAAGGGAGATTTCA

Supplementary Figure Legends

Fig. S1 Procedures for determining deletion indexes after RNP editing. **a** Primers for long-range PCR. The barcoded primers were used to amplify 4-6 kb DNA flanking the RNP target sites. Eight to 98 amplicons were combined for nanopore sequencing. **b** Schematic overview for nanopore sequencing data analysis. The demultiplexed fastq data were mapped to reference sequences using Minimap2. The deletion indexes were calculated using Samtools. **c** Deletion indexes analyzed by Samtools and ImageJ are highly correlated. Substracting background noise, two methods gave rise to identical deletion indexes. **d** Raw data of background deletions. 2-6% background noise was detected in unedited cells, reflecting the ONT sequencing errors. The levels of background deletions are amplicon-dependent. The data in **d** were shown as mean ± SEM, and the significance was determined by the two-way ANOVA test. Adjusted *p* values were indicated. "ns" means no significance ($p > 0.05$).

Fig. S2 Analysis of deletions of different sizes. **a-c** Analysis of large deletions in T cells (**a**), HSPCs (**b**) and iPSCs (**c**). The percentages of deletions larger than 100-bp (D100), 500-bp (D500), 1000-bp (D1000), 1500-bp (D1500), and 2000-bp (D2000) were shown. **d-f** Linear correlation of alleles of D100 vs. D500 (**d**), D500 vs. D1000 (**e**), D100 vs. D1000 (**f**).

Fig. S3 Cell viability and cell cycle profiles in T cells, HSPCs, and iPSCs after RNP editing. **a** Cell viability after editing in T cells, HSPCs, and iPSCs was comparable. Alive cells were enumerated 3 days after RNP transfection. The cell numbers of edited cells were normalized to those of unedited cells. **b** Chemical structure of Pyronin Y and Hoechst 33342 used in cell cycle analysis. **c** Representative FACS data of HSPC cell cycle profiles of unmanipulated cells (WT) and RNP edited cells (RNP). The G0/G1, S, and G2/M phases were gated and depicted with arrows in the FACS plot. **d** Cell cycle profiles of WT control and edited T cells, HSPCs, and iPSCs one day after RNP nucleofection. The data in **a** were examined by the one-way ANOVA test. The data in **d** were analyzed by the two-way ANOVA test. Adjusted *p* values were indicated. "ns" means no significance ($p > 0.05$).

Fig. S4 AAV6 donor-mediated HDR reduces large deletions. **a** Schematic of HDR editing with Cas9-sgRNA RNPs and promoter-less mNeonGreen AAV6 donor templates. Representative IGV presentations of PCR amplicons before and after mNeonGreen knock-in were shown. **b** Representative FACS plots. APC was an irrelevant channel. As controls, omitting gRNA or AAV donors or using mismatched gRNA and AAV donors led to 0% HDR editing. **c, d** Effects of MOI of AAV6 donors on the frequencies of NHEJ and HDR (**c**) and deletion index (**d**). Data are shown as mean ± SEM of n = 6 independent experiments from 2 editing loci. **e** Visualization of representative deletion data of the AAVS1 locus in HSPCs. Unedited wild-type cells showed a background deletion. This noise was subtracted from deletions in edited cells to obtain deletion indexes. The data in **c** and

d were statistically analyzed by the two-way ANOVA test. Adjusted *p* values were indicated. "ns" means no significance ($p > 0.05$).

Fig. S5 Removal of HDR alleles does not affect analytical results. **a** Removal of HDR alleles with mNeonGreen knock-in at *EEF2* by Seqkit tools. **b** Representative IGV display nanopore reads mapped to the HDR sequence before and after removal of HDR reads. **c** Illustration of nanopore reads mapped to the wild-type sequence. **d** Deletion indexes analyzed with or without the HDR alleles. The data in **d** was statistically analyzed by the one-way ANOVA test. Adjusted *p* values were indicated. "ns" means no significance ($p > 0.05$).

Fig. S6 Insertion of double-stranded oligonucleotides reduces large deletions. **a** Examples of small indels and dsODN insertions analyzed by amplicon sequencing and CRISPResso2. The long red boxes indicate the insertion of perfect or partially deleted 34bp-dsODN in the forward or reverse orientation. **b** The insertion of dsODN depends on the NHEJ repair pathway. The NHEJ inhibitor M3814 (2 μM) barely affects total indels but almost completely blocked dsODN insertion. **c, e** Frequencies of small indels (NHEJ) and dsODN insertions (treated as HDR by CRISPResso2 analysis) in HSPCs (**c**) and iPSCs (**e**). **d, f** dsDNA insertions decrease deletion indexes of HSPCs (**d**) and iPSCs (**f**). **g** Raw data of deletion analysis by Samtools. Error bars represent mean ± SEM of n = 3-6 independent experiments. Data in **b** were statistically analyzed by Student's T-test. The data in **c-g** were statistically analyzed by the two-way ANOVA test. Adjusted *p* values were indicated. "ns" means no significance ($p > 0.05$).

Fig. S7 Insertion of double-stranded oligonucleotides reduces large deletions in a dosage-dependent manner. **a-c** Analysis of total editings, dsODN insertions, and relative large deletions in T cells with RNP and indicated amounts of 34bp dsODN at *EEF2* (**a**), *AAVS1* (**b**), and *BCL11A-2* (**c**) target sites. **d** Correlation analysis of relative dsODN insertions (insertion frequencies/total editings) and relative deletions (normalized deletion indexes to editing without dsODNs) in edited T cells at *EEF2*, *AAVS1*, and *BCL11A-2* target sites, respectively. Raw data were from Additional file 1: Fig. S7, b for *EEF2* and *AAVS1* analysis, respectively. The data from Fig. 4b and Additional file 1: Fig. S7c were combined for *BCL11A-2* analysis. For **a-c**, error bars represent mean ± SEM of n = 3-4 independent experiments. The data in **a-c** were statistically analyzed by the one-way ANOVA test. Adjusted *p* values were indicated. "ns" means no significance ($p > 0.05$).

Fig. S8 Summary of large deletions in T cells and HSPCs by different gene-editing approaches. **a-b** Summary of relative deletion indexes (**a**) and raw deletions (**b**) of edited human cells. All the results of edited T cells and HSPCs presented in Fig. 2-4 were summarized (n = 20-50). **c** Summary of D100 data in T cells and HSPCs. **d** Correlation analysis of deletion indexes and D100 (percentage of alleles with deletion larger than 100-bp). The data in **a** and **c** were statistically analyzed by the one-way ANOVA test. Adjusted *p* values were indicated.

Fig. S9 Effects of NHEJ inhibitors on editing outcomes at the *EEF2* locus. **a** Representative editing outcomes analyzed by amplicon sequencing and CRISPResso2. WT indicates the unedited allele. Predominant NHEJ +A and MMEJ -25bp alleles were indicated. The microhomology of MMEJ was underlined in red. **b** and **c** Relative frequencies of +A NHEJ and -25bp MMEJ editings in the absence

and presence of the NHEJ inhibitors M3814 (**b**) or NU7441 (**c**). The data in **b** and **c** were statistically analyzed by the two-way ANOVA test. Adjusted *p* values were indicated. "ns" means no significance ($p > 0.05$).

Fig. S10 Analysis of large deletions in iPSC single-cell clones. Coverage of nanopore reads of WT cells (WT #1 and WT #2) and 52 edited iPSC single-cell clones shown in Fig. 6. The percentages of reads with deletions larger than 100 bp (D100) were indicated.

Fig. S11 AAV6 HDR donor sequences used in this study. Blue indicates the left homology arm; green denotes the right homology arm; the inserted sequences are shown in red.
