# Supplementary material for: Effective control of large deletions after double-strand breaks by homology-directed repair and dsODN insertion
Source: Genome Biol. 2021 Aug 20;22:236. doi: 10.1186/s13059-021-02462-4 (PMC8377869; doi:10.1186/s13059-021-02462-4)
Supplement: Supplementary file 2 — Additional file 2. Review history. [file 13059_2021_2462_MOESM2_ESM.docx]

Review History

**First round of review**

**Reviewer 1**

**Are you able to assess all statistics in the manuscript, including the appropriateness of statistical tests used?** Yes, and I have assessed the statistics in my report.

**Comments to author:**

Wen et al. investigate the occurrence of large deletions at the target locus, an often-overlooked side effect of CRISPR genome editing that may have serious implications for the genome editing field. While the appearance of on-target effects has already been described in mice (e.g., Adikusuma et al., 2018; Kosicki et al., 2018; Owens et al., 2019; Shin et al., 2017) and human cells (e.g. Kosicki et al., 2018; Weisheit et al., 2020), not much is known about factors that influence their occurrence. Therefore, the manuscript takes up an important issue with the aim to develop guidelines ensuring on-target locus integrity, which could help to increase the reliability and safety of CRISPR-based applications. The authors identify large deletions after genome editing in several different human cell types at four different loci by NHEJ or HDR-editing using ssODN or AAV-based donors, which is a good selection of the most commonly used Cas9-based editing methods in clinically relevant human cell types. The entire analysis is based on long-range PCR followed by Nanopore sequencing, which allows generation of sequences of many kilobases. While the work deals with a timely and important topic for the genome editing field and applies cutting-edge technologies, I have several major and minor concerns that I believe severely affect the reliability of experimental conclusions and impact of the manuscript for the field:

Major issues

1.     a) Quantification of on-target effects in the entire paper is based on long-range PCR followed by Nanopore sequencing. This is problematic, as the PCR introduces experimental bias that will skew the results. The PCR will only work if the occurring deletions do not affect one of the two primer-binding sites. If they do, the deletion will not be detected. Furthermore, deletions are not the only on-target effects that may occur after editing. Several recent papers have shown that large insertions or rearrangements can also be present, and these may or may not be detected by the performed long-range PCR, depending on their size. For example, a large insertion may increase locus size to a degree where it can no longer serve as a template for PCR. Or an inversion may put the two primer binding sites on the same strand, again preventing PCR amplification. A recent study by Weisheit et al. 2020, which already investigated on-target effects in human cells, described such issues with PCR amplification of the edited locus. They even exploited these PCR amplification issues to perform on-target detection by qPCR quantification. Using those methods, they detected on-targets in 10 to 40% of edited iPSC clones. In contrast, in the manuscript the detected rates are much lower, especially in iPSCs. Distressingly, this major discrepancy between published data and the manuscript is not mentioned or discussed and the paper is not even mentioned. It appears that the authors miss a large proportion of on-target effects due to the issues with their analysis method.
b) Another potential issue introduced by long-read sequencing is PCR amplification bias. When amplifying the target locus in a pool of edited cells, large deletions with shorter PCR amplicon lengths might be amplified preferably, leading to an overestimation of large deletions in the analysis. As the entire manuscript is based on long-range PCR, this should have been investigated and excluded by experimental controls.
Taken together, the flaws of the long-read sequencing analysis may severely affect the reliability of the presented data and therefore, the manuscript in its current form should not be published. Bias could be reduced by complementing the analysis of unwanted editing outcomes using additional methods, e.g. based on droplet digital PCR, quantitative PCR or analysis of single cell clones. Furthermore, it could be investigated, if analyzing the read coverage in deep sequencing-based methods that rely on short reads might be a more suitable analysis tool.

2.     Throughout the manuscript, the "deletion index" is used interchangeably with quantification of "large deletions" (e.g., Fig. 1e). However, the deletion index also contains all small/medium-sized indels. Higher editing efficiencies with many small indels might therefore strongly affect the deletion index, without necessarily meaning that more large deletions are present. Even though the editing efficiencies are mentioned separately, discriminating between different sizes of deletions should be performed during analysis of the sequencing data to optimize data interpretation.

3.     The authors claim that large deletions are reduced in the presence of ssODN or AAV. However, this seems trivial as this effect is driven by the increase in HDR editing, which by their definition are reads that contain the intended edit and no deletions. So if there are more HDR reads at the expense of NHEJ, it is logical that occurrence of large deletions will go down. The data interpretation would be clearer if HDR-reads were removed during the sequencing analysis and quantification was based only on reads without HDR. But even then, I think the analysis will not be as informative as it could be: when performing HDR-based editing in cultured cells - which is the main topic of the manuscript - the intention is usually to obtain cells that have either HDR on both alleles (in diploid cell lines, such as iPSCs) or on one allele with WT sequence on the other. Deep-sequencing analysis will however not tell us if large deletions occur in cells together with HDR, or only in those with NHEJ, which are not relevant for the intended outcome. Such an analysis would require on-target detection based on clones, which the authors did not perform. To make this relevant for the field, this should be done.

4.     In many instances throughout the manuscript the authors overstate their data, claiming that they see an effect while the results do not show any significant changes and/or no p value is given (e.g., when referring to the results in Figure S3c, S4d). This is especially apparent in the iPSC data, which is not significant in the large majority of the experiments. The authors should tone down their claims so they fit to the results, and they should remove the iPSC data from the manuscript as it is not informative.

5.     It is confusing that the authors do not see any or only a very minor effect of the NHEJ or the MMEJ inhibitors on indel frequency. Earlier work has confirmed that this should have an effect and I am therefore wondering if the inhibitors are active. Furthermore, since this experiment should be a validation of the method, I would suggest that the authors move the data and text of Figure 5 more to the beginning of the manuscript.

6.     The title of the manuscripts already suggests that the addition of dsODNs to genome editing experiments can control the occurrence of large deletions. However, it is not discussed anywhere that adding double-stranded DNA fragments poses a high risk of random integrations into the genome. It would not be helpful for the field to be able to reduce on-target effects but at the same time increase the risk of messing up the genome by random integrations. It would therefore be important to investigate this potential problem, e.g., by a method similar to GUIDE-seq, which is based on genomic DNA fragmentation, circularization and sequencing by using the dsODN donor as primer binding site.

Minor issues:

1. CRISPR editing may also induce loss-of-heterozygosity at the target locus, which is not even mentioned in the manuscript (see e.g., Ikeda et al., 2018; Weisheit et al., 2020). This even less known phenomenon should at least be discussed, although it could also be investigated somewhat easily by looking at altered SNPs in the sequencing data.

2. On p.2, line 32, the authors mention that double cut HDR donors increase HDR efficiency (probably to cite their own studies). However, to my understanding, this strategy is not used in the following manuscript, which I find confusing.

3. The text states a PCR product range of 4-6 kb, however, in Figure S1a legends it says 2-3 kb?

4. The calculation of deletions by ImageJ is mentioned and compared to the quantification by Samtools. Since the ImageJ quantification is not applied again, is it necessary to mention it at all?

5. p.3, Line 2: Which figure/data is the <1% referring to?

6. The text states that a mean background deletion of 3-4% for WT cells (p.3, line 6) exists, however, in figure legend S1d (+ data) a range of 2-6% is mentioned, which is conflicting. Furthermore, a mean does not indicate a range, but a certain value.

7. Figure legend S1a) mentions "dozens of amplicons". Please use a more scientific term to describe what was sequenced.

8. Figure 1a is mentioned after Figure 1b + 1c. Please streamline text or order of figure panels.

9. The resolution of Figure 1a and S4a is insufficient for proper inspection.

10. p.3, Line 29: I do not see which feature in the figure this sentence is referring to.

11. p.4, Line 39: Where does the conclusion come from?

12. p. 5, Line 54: reference to figure is wrong.

13. p.7, Line 13: What do the authors mean by "more significant small deletions"? Blocking NHEJ rather decreases small deletions?

14. Statistics: The statistical analysis used in this manuscript seems inappropriate for the presented data. In particular, multiple t-tests were performed in the majority of the experiments without correcting for multiple testing. Due to accumulating alpha-error with multiple testing, Bonferroni-Holm correction or similar methods should be used to be able to make an appropriate conclusion about the significance of the results. Omission of these correction methods may over-estimate the significance of the results and strongly increases the chance of false-positive significance statements. This is the case for the following data:
*     Fig. 2 b, c
*     Fig. 3 b, c
*     Fig. 4 b, c
*     Fig. S2 d, e
*     Fig. S4 b,c,d,e,f
Furthermore, paired Students t-test can only be used to compare two different groups. However, here it was also used to compare multiple groups, which seems inappropriate. Methods such as One-way ANOVA may need to be used instead. This affects the following data:
*     Fig. 4 d, e
*     Fig. S1 d
*     Fig. S2 b,c
*     Fig. S3 d
*     Fig. S4 g
*     Fig. S5
*     Fig. 1 d,e: here even Two-Way ANOVA might be more appropriate
*     Fig. S3 c: not clear what is actually compared in these data
*     Fig. S3 d: Two-way ANOVA would be appropriate, seems like the data is only significant for AAVS1 but not for EEF2; Two-Way ANOVA is required to discriminate these effects
I recommend asking a statistician to look at this in more detail.

**Reviewer 2**

**Are you able to assess all statistics in the manuscript, including the appropriateness of statistical tests used?** Yes, and I have assessed the statistics in my report.

**Comments to author:**

In this report, Wen et al. investigated long deletion outcomes after gene editing by performing Cas9 RNP electroporation of HSPCs, T cells or iPSCs followed by NanoPore long read (4-6 kb) or Illumina short-read sequencing. They find that a donor DNA template, whether ssODN or AAV, that supports HDR repair suppresses long deletions. Similarly, dsODN to produce NHEJ-mediated targeted integration also suppresses long deletions. In contrast, chemical inhibition of NHEJ repair increases the frequency of long deletion repair. Together this work suggests that including DNA templates along with Cas9 RNPs can control gene editing allelic outcomes and could be considered for applications in which long deletions are undesirable.

Overall I think these are novel and interesting observations. A strength is the effects are tested at 4 different loci and in 3 different clinically relevant cell contexts with 3 different DNA donor template types. One finding that remains surprising and unexplained is the relatively low frequency of long deletions in iPSCs as compared to T cells and HSPCs. The 2018 report from Kosicki et al (Nat Biotech) described long deletions in mouse ES cells so it seems a little surprising that human pluripotent cells would be largely resistant to this phenomenon.

A few comments:
1.     It would be very informative for the authors to report the cell cycle profile of the T-cells, HSPCs and iPSCs at the time of RNP delivery (fraction of cells in G0, G1, S, G2/M phases). It is possible that the differences in long deletions between cell types may be merely attributable to differences in cell cycle profile rather than other cell type differences.
2.     Furthermore the authors should describe the cell viability as well as the induction of p53 in the T-cells, HSPCs and iPSCs following gene editing. It is well described that pluripotent cells are particularly sensitive to p53-dependent responses to DSBs. It is possible that there is greater p53-dependent cell death and cell cycle arrest in iPSCs, so the differences in repair types might reflect the viability of cells after editing. Perhaps the cells with long deletions are the most susceptible to cell death and induce the most p53.
3.     The results with the chemical inhibitors of DNA repair M8314, NU7441 and olaparib are interesting. It would be helpful to classify the type of indel mutations as NHEJ or MMEJ repair alleles after M8314, NU7441, olaparib treatment. This could help address the following questions:
a.     Why did the M3814 treatment reduce dsODN insertion but not impact overall indel frequency which also requires NHEJ? Did M3814 treatment shift the indel types away from classical NHEJ repair and towards MMEJ repair alleles?
b.     Did olaparib treatment increase the frequency of classical NHEJ repair and reduce the frequency of MMEJ repair?
4.     Are the deletions unidirectional or bidirectional? In the 2018 Kosicki report, a substantial fraction of the long deletions were unidirectional or asymmetric in length (with one long and one short arm) with respect to the Cas9 cleavage position. Did the presence of the DNA donors (AAV, ssODN, dsODN) change the frequency of unidirectional vs bidirectional long deletions or the relative symmetry of the two deletion arms? This would have implications as to the type of DNA repair processes that produce the long deletions.

**Reviewer 3**

**Are you able to assess all statistics in the manuscript, including the appropriateness of statistical tests used?** There are no statistics in the manuscript.

**Comments to author:**

In this manuscript, the authors sought to provide techniques that would decrease the number of large deletions and indels that occur as a result of the repair pathways for double-strand breaks caused by CRISPR-Cas9 editing. The authors looked at the deletions after gene editing in three cell types: human primary T cells, hematopoietic stem and progenitor cells (HPSCs), and human induced pluripotent stem cells (iPSCs). The use of single-stranded oligonucleotides for HDR resulted in a decrease in large deletions in T cells and HSPCs. A reduction in deletions was also observed in iPSCs but much less so than in the other two cell types. AAV6 were also tested as the donor for HDR to observe whether this would reduce large deletions. The results demonstrated a decrease in large deletions in T cells and HSPCs when using the AAV6 HDR donors. Since HDR primarily occurs in dividing cells, the authors also developed methods to reduce large deletions through NHEJ of double-strand breaks. They demonstrated that the insertion of short double-stranded oligonucleotides can result in massive reduction in large deletions in T cells and HSPCs. With these findings, the authors sought to bridge the gap in bringing CRISPR-Cas9 gene editing closer to clinical translation by minimizing the possibility of detrimental deletions caused by double-strand break repair. Overall the conclusions are well supported by the data.
Major comment:
1)     There is a lack of depth in discussion as to "why" there were significant decreases in deletion indexes with the addition of either ssODN, AAV6, or dsODN. Putative hypotheses must be discussed, and at least a few experimentally explored, even if a detailed mechanism of mode of action is not eventually elucidated.
Minor comments:
1)     The introduction needs more discussion of current work that has already been done to minimize the off-target effects caused by using the CRISPR-Cas9 system.
2)     Analysis of results needs to be further explored. For instance, there was a lack of further explanation on why there was an increase in deletion indexes at the EEF2 locus for iPSCs shown in Figure 3c.

**Authors Response**

**Point-by-point responses to the reviewers’ comments:**

*Reviewer #1: Wen et al. investigate the occurrence of large deletions at the target locus, an often-overlooked side effect of CRISPR genome editing that may have serious implications for the genome editing field. While the appearance of on-target effects has already been described in mice (e.g., Adikusuma et al., 2018; Kosicki et al., 2018; Owens et al., 2019; Shin et al., 2017) and human cells (e.g. Kosicki et al., 2018; Weisheit et al., 2020), not much is known about factors that influence their occurrence. Therefore, the manuscript takes up an important issue with the aim to develop guidelines ensuring on-target locus integrity, which could help to increase the reliability and safety of CRISPR-based applications. The authors identify large deletions after genome editing in several different human cell types at four different loci by NHEJ or HDR-editing using ssODN or AAV-based donors, which is a good selection of the most commonly used Cas9-based editing methods in clinically relevant human cell types. The entire analysis is based on long-range PCR followed by Nanopore sequencing, which allows generation of sequences of many kilobases. While the work deals with a timely and important topic for the genome editing field and applies cutting-edge technologies, I have several major and minor concerns that I believe severely affect the reliability of experimental conclusions and impact of the manuscript for the field:*

*Major issues*

*1. a) Quantification of on-target effects in the entire paper is based on long-range PCR followed by Nanopore sequencing. This is problematic, as the PCR introduces experimental bias that will skew the results. The PCR will only work if the occurring deletions do not affect one of the two primer-binding sites. If they do, the deletion will not be detected. Furthermore, deletions are not the only on-target effects that may occur after editing. Several recent papers have shown that large insertions or rearrangements can also be present, and these may or may not be detected by the performed long-range PCR, depending on their size. For example, a large insertion may increase locus size to a degree where it can no longer serve as a template for PCR. Or an inversion may put the two primer binding sites on the same strand, again preventing PCR amplification. A recent study by Weisheit et al. 2020, which already investigated on-target effects in human cells, described such issues with PCR amplification of the edited locus. They even exploited these PCR amplification issues to perform on-target detection by qPCR quantification. Using those methods, they detected on-targets in 10 to 40% of edited iPSC clones. In contrast, in the manuscript the detected rates are much lower, especially in iPSCs. Distressingly, this major discrepancy between published data and the manuscript is not mentioned or discussed and the paper is not even mentioned. It appears that the authors miss a large proportion of on-target effects due to the issues with their analysis method.*

*b) Another potential issue introduced by long-read sequencing is PCR amplification bias. When amplifying the target locus in a pool of edited cells, large deletions with shorter PCR amplicon lengths might be amplified preferably, leading to an overestimation of large deletions in the analysis. As the entire manuscript is based on long-range PCR, this should have been investigated and excluded by experimental controls.*

*Taken together, the flaws of the long-read sequencing analysis may severely affect the reliability of the presented data and therefore, the manuscript in its current form should not be published. Bias could be reduced by complementing the analysis of unwanted editing outcomes using additional methods, e.g. based on droplet digital PCR, quantitative PCR or analysis of single cell clones. Furthermore, it could be investigated, if analyzing the read coverage in deep sequencing-based methods that rely on short reads might be a more suitable analysis tool.*

**Response**:

We appreciate this reviewer’s insightful and constructive critiques of our work. We have conducted qPCR and single-cell cloning to address all the posed questions.

a) Mega-deletion. We agree that PCR amplification to detect large deletions is limited on primer-binding sites. If there is a mega-deletion, we will not obtain a long PCR product of 4kb - 6kb in length. Per request, we have designed primers to examine deletions up to 160kbp upstream and downstream of the cut site by qPCR. Reassuringly, we did not detect any mega-deletions. Even considering the sensitivity of qPCR assay, the data argue that mega-deletion is not a significant concern in our RNP-based editing.

b) Chromosome rearrangement. Our study focuses on large deletions. Although it is beyond the scope of our research, we still discussed this concern. When there are multiple DSBs in a single cell, chromosome translocation may occur at 1-5%, as demonstrated by Carl June’s 2020 publication in Science. However, in our studies, only one RNP is used; thus we expect much lower translocation events, if any. We will address this important issue in the near future.

c) PCR bias. It is well-known that PCR preferentially amplifies short fragments. As shown in single-cell cloning studies, this is a well-founded concern, but it does not nullify our conclusion. We have now illustrated in the manuscript as follows: “One concern in long PCR of mixed editing events is preferential amplification of short alleles, which would lead to artificially high-level long-range deletions. To address this issue, we analyzed the single-cell clones with both long and short alleles. In two typical clones（#7 and #8）with three distinct alleles, we found that a 1479-bp and a 2716-bp deletion allele constituted 67% and 80% reads, respectively, of the relevant single-cell clones, which is considerably higher than the expected 33%. In contrast, the 13-bp deletion allele represented only 7%. These data support the notion that current PCR technologies preferentially amplify sequences with a shorter length. Accordingly, the proportion of alleles with long deletions might have been overestimated, casting doubt on the reliability of our conclusions. However, two facts argue against this concern: 1) the bias is systematic and thus does not affect the conclusion of well-controlled studies; 2) D1000, particularly D2000, makes up less than 10% of the data (Fig. S2). Thus the influence is limited.”

d) Large deletions in single cells. We have now conducted single-cell cloning to assess the reliability of our presented data. Aggregating single-cell data showed consistency with bulk cell analysis. Thus, all our conclusions based on PCR of the bulk population still hold. We have now illustrated in the manuscript as follows: “Last, we compared the deletion indexes and D100 in bulk cell populations and aggregated single-cell clone data and found similar DI in bulk cells and mean deletions in single-cell clones (Fig. 6b). Therefore, the single-cell results consolidate the conclusions of bulk cell analysis.”

e) Definition of large deletion as D100 (deletion of over 100bp). We have re-analyzed our data and found that DI correlated very well with D100. We have now illustrated in the manuscript as follows: “To quantify the levels of large deletions precisely, we used the deletion index, whereas previous studies defined removing fragments of over 100 bp (D100) as large deletions. Therefore, we extended the analysis by calculating alleles with deletions of over 100, 500, 1000, 1500, or 2000 bp (Fig. S2a-c and S2d-f). We found that over 80% of large deletions were 100-1000 bp in length, while D2000 was 0.1% or lower (Fig. S2a-c). Furthermore, even though D100 was more sensitive than deletion index (DI) in assessing large deletions, deletion indexes correlated excellently with D100 (Fig. 2e, 3e, and S8c). We thus decided to report DI in detail and also summarized D100 data for comparison.”

*2. Throughout the manuscript, the "deletion index" is used interchangeably with quantification of "large deletions" (e.g., Fig. 1e). However, the deletion index also contains all small/medium-sized indels. Higher editing efficiencies with many small indels might therefore strongly affect the deletion index, without necessarily meaning that more large deletions are present. Even though the editing efficiencies are mentioned separately, discriminating between different sizes of deletions should be performed during analysis of the sequencing data to optimize data interpretation.*

**Response**: Per request, we have now re-analyzed our data, following the norm of this field. In theory, the deletion index also contains all small/medium-sized indels. In reality, the vast majority of alleles with large deletions are D100-500. Even though small/medium-sized indels occurred more often, it does not significantly impact the DI values. In addition, deletions of different sizes are not independent events. In other words, more medium-sized deletion correlated with more large deletions, as shown in Fig S2. As such, DI correlated very well with D100, as shown in Fig 2e, 3e, and S8c. The use of the DI metric has its merit, for it unnecessities the arbitrary threshold used in defining how large is large enough to be considered a large deletion. For example, one may consider 50-bp del, and another prefers 100-bp deletion.

And we considered deletions larger than 100 bp (D100) as large deletions and found a good correlation of DI and D100 in Fig. S8. The different sizes of deletions were also analyzed and showed in Fig. S2a-c. We have also added the statement in the Results session as follows:

“To quantify the levels of large deletions precisely, we used the deletion index, whereas previous studies defined removing fragments of over 100 bp (D100) as large deletions. Therefore, we extended the analysis by calculating alleles with deletions of over 100, 500, 1000, 1500, or 2000 bp (Fig. S2a-c and S2d-f). We found that over 80% of large deletions were 100-1000 bp in length, while D2000 was 0.1% or lower (Fig. S2a-c). Furthermore, even though D100 was more sensitive than deletion index (DI) in assessing large deletions, deletion indexes correlated excellently with D100 (Fig. 2e, 3e, and S8c). We thus decided to report DI in detail and also summarized D100 data for comparison.”

*3. The authors claim that large deletions are reduced in the presence of ssODN or AAV. However, this seems trivial as this effect is driven by the increase in HDR editing, which by their definition are reads that contain the intended edit and no deletions. So if there are more HDR reads at the expense of NHEJ, it is logical that occurrence of large deletions will go down. The data interpretation would be clearer if HDR-reads were removed during the sequencing analysis and quantification was based only on reads without HDR. But even then, I think the analysis will not be as informative as it could be: when performing HDR-based editing in cultured cells - which is the main topic of the manuscript - the intention is usually to obtain cells that have either HDR on both alleles (in diploid cell lines, such as iPSCs) or on one allele with WT sequence on the other. Deep-sequencing analysis will however not tell us if large deletions occur in cells together with HDR, or only in those with NHEJ, which are not relevant for the intended outcome. Such an analysis would require on-target detection based on clones, which the authors did not perform. To make this relevant for the field, this should be done.*

**Response**: We agree with your professional concern. The data with HDR gene editing were all analyzed with both HDR and NHEJ alleles. We have clearly stated the analysis process in the manuscript especially for the large fragment HDR knock-in in *EEF2* locus. To investigate the quantification based only on reads without HDR, we have removed the HDR reads as shown in Fig. S5 and compared the deletion indexes between alleles with HDR or without HDR. And we found there were no significant differences in deletion indexes. The detailed information was stated in the Results session and Fig. S5: “At the EEF2 locus, rather than a short fragment knock-in, AAV6 mediated a mNeonGreen reporter insertion. To determine whether the HDR alleles in the dataset artificially decrease the deletion indexes, we analyzed data after depletion of HDR alleles (Fig. S5a and S5b). We found no significant differences between the reads with HDR alleles and reads without HDR alleles (Fig. S5c and S5d). These data suggest that our analytical strategy precisely captures the deletion levels.”

*4. In many instances throughout the manuscript the authors overstate their data, claiming that they see an effect while the results do not show any significant changes and/or no p value is given (e.g., when referring to the results in Figure S3c, S4d). This is especially apparent in the iPSC data, which is not significant in the large majority of the experiments. The authors should tone down their claims so they fit to the results, and they should remove the iPSC data from the manuscript as it is not informative.*

**Response**: Compared with T cells and HSPCs, large deletions in gene-edited iPSCs were low and close to the background in wild-type iPSCs. We have double-checked our manuscript and avoided overstating our data. Changes in iPSCs were largely insignificant because of the low background deletions. These seemingly negative data are essential for the field of iPSCs, given their expanding applications in clinical cell therapy.

We found relatively low and near the unedited WT cells background in large deletion after RNP gene editing, which is different from other published finding (Weisheit I *et al*., *Cell Reports*, 2020). We have performed more experiments to address issue including cell cycle analysis. This part has been considerably modified as follows: “In the above studies, we observed considerably lower deletions in iPSCs compared to T cells and HSPCs. Our iPSC-RNP-editing results are seemingly at odds with a previous study using plasmid editing in which iPSCs with high-level Cas9 expression were drug-selected. We asked if cell death after electroporation of RNPs contributed to biased results in the three types of cells. We counted cell numbers two days after transfection relative to unmanipulated counterparts as a surrogate indicator of cell survival. No significant differences in viabilities were observed (Fig. S3a). We further investigated if changes in the cell cycle played a part. We profiled the cell cycle using Pyronin Y and Hoechst 33342 co-staining one day after RNP delivery (Fig. S3b and S3c). We observed no significant differences in G0/G1, S, and G2/M phases in HSPCs (Fig. S3d), while a slight increase of T cells in the S phase and iPSCs in the G2/M phase. However, we noted considerably more iPSCs in the G2/M phase than T cells or HSPCs (~50% vs. 20%). This striking distinction might have partly contributed to ~5-fold lower deletions in edited iPSCs since the HDR is the prevailing pathway in cycling iPSCs. Together, significant low-level deletion mutagenesis in iPSCs likely results from their intrinsic nature and transient low-level intracellular Cas9.”

*5. It is confusing that the authors do not see any or only a very minor effect of the NHEJ or the MMEJ inhibitors on indel frequency. Earlier work has confirmed that this should have an effect and I am therefore wondering if the inhibitors are active. Furthermore, since this experiment should be a validation of the method, I would suggest that the authors move the data and text of Figure 5 more to the beginning of the manuscript.*

**Response**: We have found a minor effect of NHEJ or MMEJ inhibitors on indel frequency in many loci, because NHEJ blockade is offset by MMEJ repair, which leads to more short deletions. To demonstrate the efficacy of inhibitors, we have presented one typical editing outcome with one predominant +A type NHEJ and a -25bp type MMEJ (the micro homology is CAGGAAG) at *EEF2* locus. We found a significant decrease in +A NHEJ frequencies and a significant increasing in -25bp MMEJ frequencies with either M3814 or NU7441 treatment (Fig. S9). We then blocked the MMEJ pathway with Olaparib. MMEJ inhibition reduced the NHEJ repair pathway but had no significant effect on MMEJ mutations (Fig. S9). But we did not find any significant changes of MMEJ alleles when treated with Olaparib. And our recent study has also shown that Olaparib cannot reduce MMEJ mutations (Ya-Wen Fu, et al., *Nucleic Acids Research*, 2021). Unfortunately, there are no potent MMEJ inhibitors available on the market. Therefore, for the time being, the effects of MMEJ can not be meticulously examined. However, our data still suggest that HDR and NHEJ play dominant roles in curtailing DI. However, this conclusion does not suggest that MMEJ does not impact the outcomes of large deletions. In contrast, if MMEJ is totally blocked, more damages to chromosomes will ensue.

*6. The title of the manuscripts already suggests that the addition of dsODNs to genome editing experiments can control the occurrence of large deletions. However, it is not discussed anywhere that adding double-stranded DNA fragments poses a high risk of random integrations into the genome. It would not be helpful for the field to be able to reduce on-target effects but at the same time increase the risk of messing up the genome by random integrations. It would therefore be important to investigate this potential problem, e.g., by a method similar to GUIDE-seq, which is based on genomic DNA fragmentation, circularization and sequencing by using the dsODN donor as primer binding site.*

**Response**: We agree that adding double-stranded DNA fragments poses a potential risk of random integrations into the genome. We have now discussed this point in the Discussion session as follows: “However, the dsODN pose a risk to random insertion at dsDNA breaks induced by replication or other stresses. Further investigation will gain more insight into this potential concern.”

*Minor issues:*

*1. CRISPR editing may also induce* *loss-of-heterozygosity at the target locus, which is not even mentioned in the manuscript (see e.g., Ikeda et al., 2018; Weisheit et al., 2020). This even less known phenomenon should at least be discussed, although it could also be investigated somewhat easily by looking at altered SNPs in the sequencing data.*

**Response**: We have discussed this phenomenon in the discussion part. The detailed information was stated in response to major issue 1.

*2. On p.2, line 32, the authors mention that double cut HDR donors increase HDR efficiency (probably to cite their own studies). However, to my understanding, this strategy is not used in the following manuscript, which I find confusing.*

**Response**: It is true that the double cut HDR donor technology has not been applied in this manuscript, but our data suggest that HDR donors, either in the forms of ssODN, AAV or plasmid, will lead to the decrease of large deletions. It will be helpful to mention this very popular HDR donor, which has been cited in over 250 papers. Similary, we mentioned other CRISPR editing associated adverse effects, such as off-target effects, authough we were not intended to address this issue in this manuscript. To clarify , we have changed the statement to “We have previously reported efficient HDR editing in cell lines and human iPSCs using plasmid donors”.

*3. The text states a PCR product range of 4-6 kb, however, in Figure S1a legends it says 2-3 kb?*

**Response**: We have corrected the typo.

*4. The calculation of deletions by ImageJ is mentioned and compared to the quantification by Samtools. Since the ImageJ quantification is not applied again, is it necessary to mention it at all?*

**Response**: We agree that the ImageJ quantification is not applied in large-scale data analysis, but we think it is necessary to emphasize the consistency of different quantification methods in our manuscript. This information signifies the reproducibility of our work.

*5. p.3, Line 2: Which figure/data is the <1% referring to?*

**Response**: This statement was confusing. To clarify, we have deleted “<1%” and changed the statement to “We first calculated the area under the curve using ImageJ to define significant deletions.”

*6. The text states that a mean background deletion of 3-4% for WT cells (p.3, line 6) exists, however, in figure legend S1d (+ data) a range of 2-6% is mentioned, which is conflicting. Furthermore, a mean does not indicate a range, but a certain value.*

**Response**: We have calculated the mean value and change the statement to “Data from four edited loci in wild-type (WT) cells showed a mean deletion of 3.3%”.

*7. Figure legend S1a) mentions "dozens of amplicons". Please use a more scientific term to describe what was sequenced.*

**Response**: Based on our raw data, we find the number of amplicons we used in a nanopore sequencing is ranging from 8 to 98. We have changed the legend to “Eight to 98 amplicons were combined for nanopore sequencing.”

*8. Figure 1a is mentioned after Figure 1b + 1c. Please streamline text or order of figure panels.*

**Response**: We have corrected this negligence.

*9. The resolution of Figure 1a and S4a is insufficient for proper inspection.*

**Response**: We have uploaded all high-resolution images in the revised version.

*10. p.3, Line 29: I do not see which feature in the figure this sentence is referring to.*

**Response**: We have pointed out that “A white area indicates an apparent deletion around the gRNA targeting sites in the coverage of alleles from RNP-edited cells.” in the figure legend.

*11. p.4, Line 39: Where does the conclusion come from?*

**Response**: We have removed this statement.

*12. p. 5, Line 54: reference to figure is wrong.*

**Response**: We have corrected the typo.

*13. p.7, Line 13: What do the authors mean by "more significant small deletions"? Blocking NHEJ rather decreases small deletions?*

**Response**: Blocking the NHEJ pathwy increases the MMEJ mediated deletions. To clarify, we have changed the statement to “NHEJ inhibition delays the DSB repair, leading to more MMEJ mediated deletions (Fig. S9) and large disruptions”.

*14. Statistics: The statistical analysis used in this manuscript seems inappropriate for the presented data. In particular, multiple t-tests were performed in the majority of the experiments without correcting for multiple testing. Due to accumulating alpha-error with multiple testing, Bonferroni-Holm correction or similar methods should be used to be able to make an appropriate conclusion about the significance of the results. Omission of these correction methods may over-estimate the significance of the results and strongly increases the chance of false-positive significance statements. This is the case for the following data:*

** Fig. 2 b, c*

** Fig. 3 b, c*

** Fig. 4 b, c*

** Fig. S2 d, e*

** Fig. S4 b,c,d,e,f*

*Furthermore, paired Students t-test can only be used to compare two different groups. However, here it was also used to compare multiple groups, which seems inappropriate. Methods such as One-way ANOVA may need to be used instead. This affects the following data:*

** Fig. 4 d, e*

** Fig. S1 d*

** Fig. S2 b,c*

** Fig. S3 d*

** Fig. S4 g*

** Fig. S5*

** Fig. 1 d,e: here even Two-Way ANOVA might be more appropriate*

** Fig. S3 c: not clear what is actually compared in these data*

** Fig. S3 d: Two-way ANOVA would be appropriate, seems like the data is only significant for AAVS1 but not for EEF2;* *Two-Way ANOVA is required to discriminate these effects*

*I recommend asking a statistician to look at this in more detail.*

**Response**: We thank this reviewer for the professional advice on statistical analysis.

In our revised version, we use One-Way ANOVA to analyze the statistics in Fig. S3a, S5d, S7a b c, S8a c and use Two-Way ANOVA to analyze the statistics in Fig. 1d e, 2b c d, 3b c d, 4b c d, 5b c d, S1d, S2g, S3d, S4c d, S6c d e f g, S9b. We use the Student’s T-test to analyze Fig. S6b. We also stated clearly in the figure legend. And we have double-checked all the “significant” statements in our manuscript.

*Reviewer #2: In this report, Wen et al. investigated long deletion outcomes after gene editing by performing Cas9 RNP electroporation of HSPCs, T cells or iPSCs followed by NanoPore long read (4-6 kb) or Illumina short-read sequencing. They find that a donor DNA template, whether ssODN or AAV, that supports HDR repair suppresses long deletions. Similarly, dsODN to produce NHEJ-mediated targeted integration also suppresses long deletions. In contrast, chemical inhibition of NHEJ repair increases the frequency of long deletion repair. Together this work suggests that including DNA templates along with Cas9 RNPs can control gene editing allelic outcomes and could be considered for applications in which long deletions are undesirable.*

*Overall I think these are novel and interesting observations. A strength is the effects are tested at 4 different loci and in 3 different clinically relevant cell contexts with 3 different DNA donor template types. One finding that remains surprising and unexplained is the relatively low frequency of long deletions in iPSCs as compared to T cells and HSPCs. The 2018 report from Kosicki et al (Nat Biotech) described long deletions in mouse ES cells so it seems a little surprising that human pluripotent cells would be largely resistant to this phenomenon.*

*A few comments:*

*1. It would be very informative for the authors to report the cell cycle profile of the T-cells, HSPCs and iPSCs at the time of RNP delivery (fraction of cells in G0, G1, S, G2/M phases). It is possible that the differences in long deletions between cell types may be merely attributable to differences in cell cycle profile rather than other cell type differences.*

**Response**: We thank this reviewer for the professional advice. We have performed the cell cycle profiling analysis before and after RNP delivery in three cell types. See below for a detailed reply.

*2. Furthermore the authors should describe the cell viability as well as the induction of p53 in the T-cells, HSPCs and iPSCs following gene editing. It is well described that pluripotent cells are particularly sensitive to p53-dependent responses to DSBs. It is possible that* *there is greater p53-dependent cell death and cell cycle arrest in iPSCs, so the differences in repair types might reflect the viability of cells after editing. Perhaps the cells with long deletions are the most susceptible to cell death and induce the most p53.*

**Response**: Per request, we have conducted experiments to assess cell viability and cell cycle analysis, which are now presented in Fig. S3 and described in detail as follows:

“In the above studies, we observed considerably lower deletions in iPSCs compared to T cells and HSPCs. Our iPSC-RNP-editing results are seemingly at odds with a previous study using plasmid editing in which iPSCs with high-level Cas9 expression were drug-selected. We asked if cell death after electroporation of RNPs contributed to biased results in the three types of cells. We counted cell numbers two days after transfection relative to unmanipulated counterparts as a surrogate indicator of cell survival. No significant differences in viabilities were observed (Fig. S3a). We further investigated if changes in the cell cycle played a part. We profiled the cell cycle using Pyronin Y and Hoechst 33342 co-staining one day after RNP delivery (Fig. S3b and S3c). We observed no significant differences in G0/G1, S, and G2/M phases in HSPCs (Fig. S3d), while a slight increase of T cells in the S phase and iPSCs in the G2/M phase. However, we noted considerably more iPSCs in the G2/M phase than T cells or HSPCs (~50% vs. 20%). This striking distinction might have partly contributed to ~5-fold lower deletions in edited iPSCs since the HDR is the prevailing pathway in cycling iPSCs. Together, significant low-level deletion mutagenesis in iPSCs likely results from their intrinsic nature and transient low-level intracellular Cas9.”

We have also discussed the effects of p53-dependent cell death and cell cycle arrest in iPSCs in the Discussion session as follows: “Of interest, although a previous report has shown that large, mono-allelic deletions are widespread in human iPSCs edited by plasmids transfection and puromycin selection. In contrast, human iPSCs in this study showed low-level large deletions and no appreciable mega-deletions surrounding the Cas9-sgRNA cut site. (Fig. S2). This discrepancy should attribute to the different approaches for delivering editing components. It is well-known that plasmid-mediated high-level expression of CRISPR increases off-target effects by over 10-fold relative to RNP delivery. As such, we speculate that RNP editing may also decrease large deletion incidents. Even so, RNP still led to salient disruptions in HSPCs and T cells. We reason that two factors might have contributed to the protection of iPSCs from significant damages: 1) In response to DSBs, iPSCs are prone to p53-dependent cell death and cell cycle arrest, leading to selection againt disrupted cells; 2) After RNP delivery, ~50% iPSCs were in the G2/M cell cycle，whereas only ~20% HSPCs or T cells were in the same phase. Cells in the G2/M phase are proficient in HDR, leaving less chance for erroneous DNA repair.”

*3. The results with the chemical inhibitors of DNA repair M8314, NU7441 and olaparib are interesting. It would be helpful to classify the type of indel mutations as NHEJ or MMEJ repair alleles after M3814, NU7441, olaparib treatment. This could help address the following questions:*

*a. Why did the M3814 treatment reduce dsODN insertion but not impact overall indel frequency which also requires NHEJ? Did M3814 treatment shift the indel types away from classical NHEJ repair and towards MMEJ repair alleles?*

*b. Did olaparib treatment increase the frequency of classical NHEJ repair and reduce the frequency of MMEJ repair?*

**Response**: We analyzed the data and presented the most predominant +A type NHEJ and -25bp type MMEJ (the microhomology is CAGGAAG indicated in the figures as listed below) in *EEF2* locus for detailed analysis. We found a significant decrease in +A type NHEJ frequencies and a significant increase in -25bp MMEJ type frequencies with either M3814 or NU7441 treatment (Fig. S9). Since the rise in MMEJ made up for the decrease in NHEJ, there was no change in the total indels.

We also blocked the MMEJ pathway with Olaparib. MMEJ inhibition reduced the NHEJ repair pathway but had no significant effect on MMEJ mutations (Fig. S9).

Probabally, Olaparib is not a strong or specific inhibitor of MMEJ.

*4. Are the deletions unidirectional or bidirectional? In the 2018 Kosicki report, a substantial fraction of the long deletions were unidirectional or asymmetric in length (with one long and one short arm) with respect to the Cas9 cleavage position. Did the presence of the DNA donors (AAV, ssODN, dsODN) change the frequency of unidirectional vs bidirectional long deletions or the relative symmetry of the two deletion arms? This would have implications as to the type of DNA repair processes that produce the long deletions.*

**Response**: To address this important question, we have conducted single-cell cloning analysis. We sequenced 11 clones edited with high-level Cas9 and sgRNA in the presence of M3814 to strikingly increase large deletions. We found four clones with unidirectional deletions, including 2 clones in one arm and 2 clones in another arm. And four clones showed the deletion occurred in asymmetric in length. These data indicated that large deletions are unidirectional or asymmetric in size. From our sequencing in bulk cells, we consider that the reduced large deletions are bidirectional. The single clone sequencing data are detailed as follows:

*Reviewer #3: In this manuscript, the authors sought to provide techniques that would decrease the number of large deletions and indels that occur as a result of the repair pathways for double-strand breaks caused by CRISPR-Cas9 editing. The authors looked at the deletions after gene editing in three cell types: human primary T cells, hematopoietic stem and progenitor cells (HPSCs), and human induced pluripotent stem cells (iPSCs). The use of single-stranded oligonucleotides for HDR resulted in a decrease in large deletions in T cells and HSPCs. A reduction in deletions was also observed in iPSCs but much less so than in the other two cell types. AAV6 were also tested as the donor for HDR to observe whether this would reduce large deletions. The results demonstrated a decrease in large deletions in T cells and HSPCs when using the AAV6 HDR donors. Since HDR primarily occurs in dividing cells, the authors also developed methods to reduce large deletions through NHEJ of*

*double-strand breaks. They demonstrated that the insertion of short double-stranded oligonucleotides can result in massive reduction in large deletions in T cells and HSPCs. With these findings, the authors sought to bridge the gap in bringing CRISPR-Cas9 gene editing closer to clinical translation by minimizing the possibility of detrimental deletions caused by double-strand break repair. Overall the conclusions are well supported by the data.*

*Major comment:*

*1) There is a lack of depth in discussion as to "why" there were significant decreases in deletion indexes with the addition of either ssODN, AAV6, or dsODN. Putative hypotheses must be discussed, and at least a few experimentally explored, even if a detailed mechanism of mode of action is not eventually elucidated.*

**Response**: We appreciate this insightful advice. We have recently reported that the addition of different DNA donors may influence the dynamics and patterns of CRISPR-Cas9 editing (Ya-Wen Fu, et al., *Nucleic Acids Research*, 2021). As such, timely repair of the DSB caused by CRISPR-Cas9 will minimize disruptive changes in the genome. We have now discussed the mechanism of the mode of action as follows:

“Following DSBs after CRISPR-Cas9 cleavage, DNA repair machinery is activated and recruited to promote end ligations through several damage repair pathways. These include NHEJ, alternative end-joining or microhomology-mediated end joining (alt-EJ/MMEJ), and HDR in the presence of a donor template flanked with homology arms. This process results in random repair outcomes in the absence of a donor template, leading to small indels at high frequencies and large deletions at lower frequencies. Our recent study examined the editing dynamics and patterns of CRISPR-Cas9 editing, revealing that short indels (such as +A or +T type NHEJ) occur faster than longer deletions (> 2 bp), and the AAV6-mediated HDR occurs faster than MMEJ but slower than NHEJ. As such, we hypothesize that the timely repair of DSBs decreases the events of large deletions, which is primarily a consequence of MMEJ repair. Our data in this study also shows that the inhibition of NHEJ by small molecules M3814 or NU7441 disrupts the NHEJ dependent repair. Therefore, NHEJ inhibition delays the DSB repair, leading to more MMEJ mediated deletions (Fig. S9) and large deletion mutations. On the other hand, in the presence of an HDR template, the damaged ends would have a greater chance to be fixed by homologous recombination promptly, thereby reducing the possibilities of large deletions. Cas9-gRNA predominantly creates blunt ends, which can be correctly linked by NHEJ. However, the reemergence of the Cas9-gRNA target site leads to a secondary cut. Our unique finding was that providing a short dsODN leads to its insertion at the DSBs, preventing further damage. Together, timely repair of DSBs decreases large deletions, whereas delayed restoration heightens the possibility of severe damage to the genome.”

*Minor comments:*

*1) The introduction needs more discussion of current work that has already been done to minimize the off-target effects caused by using the CRISPR-Cas9 system.*

**Response**: Considering the relation of off-target effects and our study, we have added discussions of optimized technology in reducing off-target effects in the background part as follows:

“Initial concerns about the off-target activity have been addressed by the development of sensitive detection methods (Tsai SQ *et al.*, *Nat Biotechnology*, 2015; Lazzarotto CR *et al.*, *Nat Biotechnology*, 2020; Frock RL *et al., Nat Biotechnology*, 2015), as well as modified Cas9 enzymes (Kleinstiver BP *et al., Nature*, 2016; Slaymaker IM *et al., Science*, 2016) and improved delivery protocols (Tsai SQ *et al.*, *Nat Rev Genet*, 2016) that limit this type of damage.”

*2) Analysis of results needs to be further explored. For instance, there was a lack of further explanation on why there was an increase in deletion indexes at the EEF2 locus for iPSCs shown in Figure 3c.*

**Response**: As detailed above, we have re-analyzed the data, such as D100, etc.

We observed an increase in the deletion indexes at the EEF2 locus of iPSCs (Fig. 3c). We noticed that the background noise of unedited iPSCs in the EEF2 locus was 4%. AAV6 donor addition appeared to increase deletion levels, and the added value was less than 0.5%. This result might arise from unknown perturbations. We have double-checked our data and found there is no mistake, such as sample switch. To ensure the authenticity of our data, we decided to publish this unexpected outlier result, even although we can not offer a satisfactory explanation for the time being.

**Second round of review**

**Reviewer 1**

Wen et al. 2nd Review:

I was asked to look at a revised version of the manuscript after I already reviewed the initially submitted version. While the original paper had some informative experiments about the occurrence of large deletions after CRISPR genome editing, I had pointed out several flaws that affected the reliability of the experimental conclusions for the field. First, the entire analysis was based on Nanopore sequencing, but not validated by a second, independent method. Second, the presented data included all types of deletions around the target locus, measured by the deletion index, although the manuscript aimed to address large deletions only. And third, contradicting published data the manuscript claimed that large deletions do not occur at a significant rate in iPSCs, which was not convincingly shown.
Generally speaking, I appreciate the authors efforts to address every point that was criticized initially, often with new experimental data, and most of the minor points were sufficiently addressed. However, the abovementioned major concerns were not sufficiently addressed, as I have detailed below for each point individually.

(my initial comments were removed as the system has a size limit)

Major issues:

Response 1:
We appreciate this reviewer’s insightful and constructive critiques of our work. We have
conducted qPCR and single-cell cloning to address all the posed questions.
a) Mega-deletion. We agree that PCR amplification to detect large deletions is limited
on primer-binding sites. If there is a mega-deletion, we will not obtain a long PCR
product of 4kb - 6kb in length. Per request, we have designed primers to examine
deletions up to 160kbp upstream and downstream of the cut site by qPCR. Reassuringly,
we did not detect any mega-deletions. Even considering the sensitivity of qPCR assay,
the data argue that mega-deletion is not a significant concern in our RNP-based editing.

This analysis is not very informative. First, the data points for the RNP-edited cells (Fig. S2g) shows quite high variation, which is in strong contrast to the WT cells and not discussed in the text. Second, qPCR analysis of cell pools is not a suitable method to identify large deletions – the proportion of large deletions is probably in general too low to detect significant changes. The only way to get informative data by qPCR would be perform it on single cell clones. Lastly, it is not clearly defined what is meant by “mega-deletions”.

b) Chromosome rearrangement. Our study focuses on large deletions. Although it is
beyond the scope of our research, we still discussed this concern. When there are
multiple DSBs in a single cell, chromosome translocation may occur at 1-5%, as
demonstrated by Carl June’s 2020 publication in Science. However, in our studies, only
one RNP is used; thus we expect much lower translocation events, if any. We will
address this important issue in the near future.

Rearrangements may not always be entire chromosome translocations, but could be smaller, more complex changes around the target locus, which happen quite frequently, as e.g. shown by Weisheit et al., Cell Reports, 2020, or Kosicki et al., Nature Biotech, 2018. This is supported by the single cell data in the manuscript – two out of 11 clones had a triplication of the locus! Therefore, this issue should be discussed in more detail in the discussion (currently it is only briefly mentioned in the introduction). At a minimum, it should be clearly stated that rearrangements and insertion are a somewhat frequent phenomenon after editing that can affect integrity of the edited locus and chromosome, and that the experiments in the manuscript do not address these on-target effects.

c) PCR bias. It is well-known that PCR preferentially amplifies short fragments. As
shown in single-cell cloning studies, this is a well-founded concern, but it does not
nullify our conclusion. We have now illustrated in the manuscript as follows: “One
concern in long PCR of mixed editing events is preferential amplification of short
alleles, which would lead to artificially high-level long-range deletions. To address this
issue, we analyzed the single-cell clones with both long and short alleles. In two typical
clones（#7 and #8）with three distinct alleles, we found that a 1479-bp and a 2716-bp
deletion allele constituted 67% and 80% reads, respectively, of the relevant single-cell
clones, which is considerably higher than the expected 33%. In contrast, the 13-bp
deletion allele represented only 7%. These data support the notion that current PCR
technologies preferentially amplify sequences with a shorter length. Accordingly, the
proportion of alleles with long deletions might have been overestimated, casting doubt
on the reliability of our conclusions. However, two facts argue against this concern: 1)
the bias is systematic and thus does not affect the conclusion of well-controlled studies;
2) D1000, particularly D2000, makes up less than 10% of the data (Fig. S2). Thus the
influence is limited.”

I recommend changing the last sentence to: ...as the bias is systematic and affects all experiments to the same degree, it should not affect our conclusions...;

d) Large deletions in single cells. We have now conducted single-cell cloning to assess
the reliability of our presented data. Aggregating single-cell data showed consistency
with bulk cell analysis. Thus, all our conclusions based on PCR of the bulk population
still hold. We have now illustrated in the manuscript as follows: “Last, we compared
the deletion indexes and D100 in bulk cell populations and aggregated single-cell clone
data and found similar DI in bulk cells and mean deletions in single-cell clones (Fig.
6b). Therefore, the single-cell results consolidate the conclusions of bulk cell analysis.”

I strongly disagree with the authors conclusion that the performed experiments support reliability of their data. It is completely unclear to me why the authors suddenly switched to a different cell line for their single cell clone analysis and again performed the analysis using Nanopore sequencing. Single cell cloning e.g., for iPSCs and other methods to detect deletions etc. have been well described in previous literature. The performed experiments only confirm that the sequencing method produced the same data in different cells.

It does not confirm, however, if Nanopore sequencing detects all potential deletions or other on-target genomic modifications in the originally edited cells! For that, a completely independent analysis, e.g. by Sanger sequencing, qPCR etc., in the same cell types would be essential to confirm the validity of the nanopore results. As the manuscript contradicts published studies about the frequency of deletions, especially in iPSCs, it is critical that all claims are shown convincingly.
Furthermore, coverage of bulk cell analysis in Fig. 6a shows approximately 50% WT alleles within the cell population, however, only one allele in the single cell clones (clone #4) has one WT allele. Where does this discrepancy between bulk and single cell clone analysis come from? As discussed in 1c), large deletions should rather be overrepresented in bulk Nanopore sequencing.

e) Definition of large deletion as D100 (deletion of over 100bp). We have re-analyzed
our data and found that DI correlated very well with D100. We have now illustrated in
the manuscript as follows: “To quantify the levels of large deletions precisely, we used
the deletion index, whereas previous studies defined removing fragments of over 100
bp (D100) as large deletions. Therefore, we extended the analysis by calculating alleles
with deletions of over 100, 500, 1000, 1500, or 2000 bp (Fig. S2a-c and S2d-f). We
found that over 80% of large deletions were 100-1000 bp in length, while D2000 was
0.1% or lower (Fig. S2a-c). Furthermore, even though D100 was more sensitive than
deletion index (DI) in assessing large deletions, deletion indexes correlated excellently
with D100 (Fig. 2e, 3e, and S8c). We thus decided to report DI in detail and also
summarized D100 data for comparison.”

First of all, I still think it is still not sufficiently explained how the “deletion index” is obtained. This will confuse a potential reader.
“…defined removing fragments of over 100 bp (D100) as large deletions.” Is this rather supposed to mean “…defined removing fragments below 100 bp (D100) as large deletions”?
Of course, the deletion index and D100 will correlate to a large extent, however, only D100 actually assesses large deletions, which is the main focus of the manuscript. To support the claims of the manuscript, all main figures should therefore mainly focus on D100 and not the deletion index.

Response 2: Per request, we have now re-analyzed our data, following the norm of this
field. In theory, the deletion index also contains all small/medium-sized indels. In reality,
the vast majority of alleles with large deletions are D100-500. Even though
small/medium-sized indels occurred more often, it does not significantly impact the DI
values. In addition, deletions of different sizes are not independent events. In other
words, more medium-sized deletion correlated with more large deletions, as shown in
Fig S2. As such, DI correlated very well with D100, as shown in Fig 2e, 3e, and S8c.
The use of the DI metric has its merit, for it unnecessities the arbitrary threshold used
in defining how large is large enough to be considered a large deletion. For example,
one may consider 50-bp del, and another prefers 100-bp deletion.
And we considered deletions larger than 100 bp (D100) as large deletions and found a
good correlation of DI and D100 in Fig. S8. The different sizes of deletions were also
analyzed and showed in Fig. S2a-c. We have also added the statement in the Results
session as follows:
“To quantify the levels of large deletions precisely, we used the deletion index, whereas
previous studies defined removing fragments of over 100 bp (D100) as large deletions.
Therefore, we extended the analysis by calculating alleles with deletions of over 100,
500, 1000, 1500, or 2000 bp (Fig. S2a-c and S2d-f). We found that over 80% of large
deletions were 100-1000 bp in length, while D2000 was 0.1% or lower (Fig. S2a-c).
Furthermore, even though D100 was more sensitive than deletion index (DI) in
assessing large deletions, deletion indexes correlated excellently with D100 (Fig. 2e,
3e, and S8c). We thus decided to report DI in detail and also summarized D100 data for
comparison.”

see answer to 1e)

Response 3: We agree with your professional concern. The data with HDR gene editing
were all analyzed with both HDR and NHEJ alleles. We have clearly stated the analysis
process in the manuscript especially for the large fragment HDR knock-in in EEF2
locus. To investigate the quantification based only on reads without HDR, we have
removed the HDR reads as shown in Fig. S5 and compared the deletion indexes
between alleles with HDR or without HDR. And we found there were no significant
differences in deletion indexes. The detailed information was stated in the Results
session and Fig. S5: “At the EEF2 locus, rather than a short fragment knock-in, AAV6
mediated a mNeonGreen reporter insertion. To determine whether the HDR alleles in
the dataset artificially decrease the deletion indexes, we analyzed data after depletion
of HDR alleles (Fig. S5a and S5b). We found no significant differences between the
reads with HDR alleles and reads without HDR alleles (Fig. S5c and S5d). These data
suggest that our analytical strategy precisely captures the deletion levels.”

No further comments.

Response 4: Compared with T cells and HSPCs, large deletions in gene-edited iPSCs
were low and close to the background in wild-type iPSCs. We have double-checked our
manuscript and avoided overstating our data. Changes in iPSCs were largely
insignificant because of the low background deletions. These seemingly negative data
are essential for the field of iPSCs, given their expanding applications in clinical cell
therapy.
We found relatively low and near the unedited WT cells background in large deletion
after RNP gene editing, which is different from other published finding (Weisheit I et
al., Cell Reports, 2020). We have performed more experiments to address issue
including cell cycle analysis. This part has been considerably modified as follows: “In
the above studies, we observed considerably lower deletions in iPSCs compared to T
cells and HSPCs. Our iPSC-RNP-editing results are seemingly at odds with a previous
study using plasmid editing in which iPSCs with high-level Cas9 expression were drugselected.
We asked if cell death after electroporation of RNPs contributed to biased
results in the three types of cells. We counted cell numbers two days after transfection
relative to unmanipulated counterparts as a surrogate indicator of cell survival. No
significant differences in viabilities were observed (Fig. S3a). We further investigated
if changes in the cell cycle played a part. We profiled the cell cycle using Pyronin Y
and Hoechst 33342 co-staining one day after RNP delivery (Fig. S3b and S3c). We
observed no significant differences in G0/G1, S, and G2/M phases in HSPCs (Fig. S3d),
while a slight increase of T cells in the S phase and iPSCs in the G2/M phase. However,
we noted considerably more iPSCs in the G2/M phase than T cells or HSPCs (~50% vs.
20%). This striking distinction might have partly contributed to ~5-fold lower deletions
in edited iPSCs since the HDR is the prevailing pathway in cycling iPSCs. Together,
significant low-level deletion mutagenesis in iPSCs likely results from their intrinsic
nature and transient low-level intracellular Cas9.”

Regarding this issue, I disagree with the authors on three different points:
First, in contrast to what the authors state above, recent publications have indeed shown high levels of large deletions in iPSCs, mouse and human cell lines (e.g. Weisheit et al., Nature Protocols, 2021, or Kosicki et al., Nature Biotech, 2018) also after RNP-based CRISPR editing. Therefore, the sentences…
p.9, line 41 “Our iPSC-RNP-editing results are seemingly at odds with a previous
study using plasmid editing in which iPSCs with high-level Cas9 expression were drugselected.”
p.9, line 6 “As such, we speculate that RNP editing may also decrease large deletion incidents.”
…should be modified or entirely removed from the text

Second, the authors state that “large deletions in gene-edited iPSCs were low and close to the background in wild-type iPSCs” (see Fig. 1d, the deletion index is close to 0! for iPSCs). However, although they can hardly detect large deletions in iPSCs in the first place, the authors still try to find factors that influence the occurrence of large deletions in iPSCs. This is not very informative. I therefore suggest to remove all iPSC data, (see also comment on Fig. 1d in third point), from the manuscript!

Third, the low level of deletions in iPSCs is in contrast to published studies that show up to 40% of edited clones having deletions or other on-target effects (Weisheit et al., Cell Reports, 2020). As already mentioned, it remains unclear if this discrepancy is due to lower levels of deletions in their cells or their inability to reliably detect them all. Again, this discrepancy needs to be mentioned and discussed clearly, further investigated by experimental data, or completely removed from the manuscript! Telling the genome editing field that large deletions do not occur in iPSCs (although published data clearly shows the opposite) is not helpful, but might rather be a wrong “all-clear signal”!  

Response 5: We have found a minor effect of NHEJ or MMEJ inhibitors on indel
frequency in many loci, because NHEJ blockade is offset by MMEJ repair, which leads
to more short deletions. To demonstrate the efficacy of inhibitors, we have presented
one typical editing outcome with one predominant +A type NHEJ and a -25bp type
MMEJ (the micro homology is CAGGAAG) at EEF2 locus. We found a significant
decrease in +A NHEJ frequencies and a significant increasing in -25bp MMEJ
frequencies with either M3814 or NU7441 treatment (Fig. S9). We then blocked the
MMEJ pathway with Olaparib. MMEJ inhibition reduced the NHEJ repair pathway but
had no significant effect on MMEJ mutations (Fig. S9). But we did not find any
significant changes of MMEJ alleles when treated with Olaparib. And our recent study
has also shown that Olaparib cannot reduce MMEJ mutations (Ya-Wen Fu, et al.,
Nucleic Acids Research, 2021). Unfortunately, there are no potent MMEJ inhibitors
available on the market. Therefore, for the time being, the effects of MMEJ can not be
meticulously examined. However, our data still suggest that HDR and NHEJ play
dominant roles in curtailing DI. However, this conclusion does not suggest that MMEJ
does not impact the outcomes of large deletions. In contrast, if MMEJ is totally blocked,
more damages to chromosomes will ensue.

First, the resolution of Figure S9a is too low for proper inspection.
Second, a comment on sentence in the answer above: “MMEJ inhibition reduced the NHEJ repair pathway but had no significant effect on MMEJ mutations (Fig. S9).” In Figure S9b, there is no effect on neither NHEJ, nor MMEJ repair by Olaparib (which was supposed to inhibit MMEJ). This clearly shows that this inhibitor does not have any effect, the data should be removed from the manuscript.


Response 6: We agree that adding double-stranded DNA fragments poses a potential risk
of random integrations into the genome. We have now discussed this point in the
Discussion session as follows: “However, the dsODN pose a risk to random insertion
at dsDNA breaks induced by replication or other stresses. Further investigation will
gain more insight into this potential concern.”

Add reference (e.g. to GUIDE-seq protocol). Rest is okay.

Minor issues:

Response 1: We have discussed this phenomenon in the discussion part. The detailed
information was stated in response to major issue 1.

From our understanding, the authors addressed this point with a sentence on p.10, line 8
“Loss-of-heterozygosity may arise in the target locus was not investigated [27].”
Please make this sentence clearer, e.g. “…occurrence of loss-of-heterozygosity was shown in a previous study investigating deleterious on-target effects after CRISPR editing[27], but was not investigated in our experiments...”

Response 5: This statement was confusing. To clarify, we have deleted “<1%” and
changed the statement to “We first calculated the area under the curve using ImageJ to
define significant deletions.”

Please clarify the expression “area under the curve”. Also, what is “mean depth” in the following sentence? (p.3, line 8)

Response 9: We have uploaded all high-resolution images in the revised version.

In the file we received, the resolution seemed to have gotten worse than it was last time. Same applies e.g., for Figure 3a and S9a.

13. p.7, Line 13: What do the authors mean by "more significant small deletions"?
Blocking NHEJ rather decreases small deletions?

Response 13: Blocking the NHEJ pathwy increases the MMEJ mediated deletions. To
clarify, we have changed the statement to “NHEJ inhibition delays the DSB repair,
leading to more MMEJ mediated deletions (Fig. S9) and large disruptions”.

This sentence is confusing, since MMEJ is also a DSB repair pathway. I suggest to make this clearer.


Further new comments:

1. p.3, line 12. “The deletion indexes analyzed by Samtools and ImageJ showed an excellent linear correlation (R2 = 0.98) (Fig. 1b and S1c).”

Which data in Figure 1b is this sentence referring to?

2. p.8, line 28 “The large fragment removal occurred after CRISPR-Cas9 induced gene editing in mouse embryonic stem cells and human embryos [10, 11].”

What is meant by “large fragment removal?”

2. p.8, line 58: This sentence does not make sense.

**Reviewer 2**

The authors have addressed my concerns.

**Authors Response**

**Point-by-point responses to the reviewers’ comments:**

*Reviewer #1:*

*I was asked to look at a revised version of the manuscript after I already reviewed the initially submitted version. While the original paper had some informative experiments about the occurrence of large deletions after CRISPR genome editing, I had pointed out several flaws that affected the reliability of the experimental conclusions for the field. First, the entire analysis was based on Nanopore sequencing, but not validated by a second, independent method. Second, the presented data included all types of deletions around the target locus, measured by the deletion index, although the manuscript aimed to address large deletions only. And third, contradicting published data the manuscript claimed that large deletions do not occur at a significant rate in iPSCs, which was not convincingly shown.
Generally speaking, I appreciate the authors efforts to address every point that was criticized initially, often with new experimental data, and most of the minor points were sufficiently addressed. However, the abovementioned major concerns were not sufficiently addressed, as I have detailed below for each point individually.

(my initial comments were removed as the system has a size limit)*

**Response:** We are impressed by this reviewer’s professionalism by reviewing our manuscript twice. We have addressed the concerns below point by point. And we hope our revised manuscript will thoroughly address your concerns.

*Major issues:

Response 1:
We appreciate this reviewer’s insightful and constructive critiques of our work. We have
conducted qPCR and single-cell cloning to address all the posed questions.
a) Mega-deletion. We agree that PCR amplification to detect large deletions is limited
on primer-binding sites. If there is a mega-deletion, we will not obtain a long PCR
product of 4kb - 6kb in length. Per request, we have designed primers to examine
deletions up to 160kbp upstream and downstream of the cut site by qPCR. Reassuringly,
we did not detect any mega-deletions. Even considering the sensitivity of qPCR assay,
the data argue that mega-deletion is not a significant concern in our RNP-based editing.

This analysis is not very informative. First, the data points for the RNP-edited cells (Fig. S2g) shows quite high variation, which is in strong contrast to the WT cells and not discussed in the text. Second, qPCR analysis of cell pools is not a suitable method to identify large deletions – the proportion of large deletions is probably in general too low to detect significant changes. The only way to get informative data by qPCR would be perform it on single cell clones. Lastly, it is not clearly defined what is meant by “mega-deletions”.*

**Response:** Per request, we have now shown our single-cell clone data from iPSCs instead of K562 cells. We also conducted qPCR to assess relative copies of gDNA at 40, 80, 160 kb away from the target site compared to unedited WT iPSCs. This analysis did not identify any appreciable changes in chromosome copy numbers in iPSCs after RNP-editing and single-cell cloning (Fig. 6d).

*b) Chromosome rearrangement. Our study focuses on large deletions. Although it is
beyond the scope of our research, we still discussed this concern. When there are
multiple DSBs in a single cell, chromosome translocation may occur at 1-5%, as
demonstrated by Carl June’s 2020 publication in Science. However, in our studies, only
one RNP is used; thus we expect much lower translocation events, if any. We will
address this important issue in the near future.

Rearrangements may not always be entire chromosome translocations, but could be smaller, more complex changes around the target locus, which happen quite frequently, as e.g. shown by Weisheit et al., Cell Reports, 2020, or Kosicki et al., Nature Biotech, 2018. This is supported by the single cell data in the manuscript – two out of 11 clones had a triplication of the locus! Therefore, this issue should be discussed in more detail in the discussion (currently it is only briefly mentioned in the introduction). At a minimum, it should be clearly stated that* *rearrangements and insertion are a somewhat frequent phenomenon after editing that can affect integrity of the edited locus and chromosome, and that the experiments in the manuscript do not address these on-target effects.*

**Response:** We have clearly stated this in the manuscript.

*c) PCR bias. It is well-known that PCR preferentially amplifies short fragments. As
shown in single-cell cloning studies, this is a well-founded concern, but it does not
nullify our conclusion. We have now illustrated in the manuscript as follows: “One
concern in long PCR of mixed editing events is preferential amplification of short
alleles, which would lead to artificially high-level long-range deletions. To address this
issue, we analyzed the single-cell clones with both long and short alleles. In two typical
clones（#7 and #8）with three distinct alleles, we found that a 1479-bp and a 2716-bp
deletion allele constituted 67% and 80% reads, respectively, of the relevant single-cell
clones, which is considerably higher than the expected 33%. In contrast, the 13-bp
deletion allele represented only 7%. These data support the notion that current PCR
technologies preferentially amplify sequences with a shorter length. Accordingly, the
proportion of alleles with long deletions might have been overestimated, casting doubt
on the reliability of our conclusions. However, two facts argue against this concern: 1)
the bias is systematic and thus does not affect the conclusion of well-controlled studies;
2) D1000, particularly D2000, makes up less than 10% of the data (Fig. S2). Thus the
influence is limited.”

I recommend changing the last sentence to: ...as the bias is systematic and affects all experiments to the same degree, it should not affect our conclusions...;*

**Response:** We have corrected this sentence. *d) Large deletions in single cells. We have now conducted single-cell cloning to assess
the reliability of our presented data. Aggregating single-cell data showed consistency
with bulk cell analysis. Thus, all our conclusions based on PCR of the bulk population
still hold. We have now illustrated in the manuscript as follows: “Last, we compared
the deletion indexes and D100 in bulk cell populations and aggregated single-cell clone
data and found similar DI in bulk cells and mean deletions in single-cell clones (Fig.
6b). Therefore, the single-cell results consolidate the conclusions of bulk cell analysis.”

I strongly disagree with the authors conclusion that the performed experiments support reliability of their data. It is completely unclear to me why the authors suddenly switched to a different cell line for their single cell clone analysis and again performed the analysis using Nanopore sequencing. Single cell cloning e.g., for iPSCs and other methods to detect deletions etc. have been well described in previous literature. The performed experiments only confirm that the sequencing method produced the same data in different cells.

It does not confirm, however, if Nanopore sequencing detects all potential deletions or other on-target genomic modifications in the originally edited cells! For that, a completely independent analysis, e.g. by Sanger sequencing, qPCR etc., in the same cell types would be essential to confirm the validity of the nanopore results. As the manuscript contradicts published studies about the frequency of deletions, especially in iPSCs, it is critical that all claims are shown convincingly.
Furthermore, coverage of bulk cell analysis in Fig. 6a shows approximately 50% WT alleles within the cell population, however, only one allele in the single cell clones (clone #4) has one WT allele. Where does this discrepancy between bulk and single cell clone analysis come from? As discussed in 1c), large deletions should rather be overrepresented in bulk Nanopore sequencing.*

**Response:** Now, we have presented iPSC single-cell clone data. We sequenced a total of 52 gene-edited iPSC single-cell clones. And we found only one clone (#31) showed a large deletion around the targeting site. All other clones showed no large deletions. We identified two SNPs around the target sites and were used to assess the potential larger deletions or loss of heterozygosity in a single-cell clone level. In each SNP, we calculated the percentage of each SNP. The clones with heterozygous alleles will show 50% of the single-nucleotide of SNPs, whereas the clones with loss-of-heterozygosity will display 0% or 100% of each SNP. The data suggested that all the iPSC single-cell clones were heterozygous, indicative of no loss-of-heterozygosity. We also assessed the copy numbers of DNA fragments 40, 80, 160 kb away from the target sites. We found no significant changes of copy number between wide-type cells and edited iPSC single-cell clones.

*e) Definition of large deletion as D100 (deletion of over 100bp). We have re-analyzed
our data and found that DI correlated very well with D100. We have now illustrated in
the manuscript as follows: “To quantify the levels of large deletions precisely, we used
the deletion index, whereas previous studies defined removing fragments of over 100
bp (D100) as large deletions. Therefore, we extended the analysis by calculating alleles
with deletions of over 100, 500, 1000, 1500, or 2000 bp (Fig. S2a-c and S2d-f). We
found that over 80% of large deletions were 100-1000 bp in length, while D2000 was
0.1% or lower (Fig. S2a-c). Furthermore, even though D100 was more sensitive than
deletion index (DI) in assessing large deletions, deletion indexes correlated excellently
with D100 (Fig. 2e, 3e, and S8c). We thus decided to report DI in detail and also
summarized D100 data for comparison.”

First of all, I still think it is still not sufficiently explained how the “deletion index” is obtained. This will confuse a potential reader.
“…defined removing fragments of over 100 bp (D100) as large deletions.” Is this rather supposed to mean “…defined removing fragments below 100 bp (D100) as large deletions”?
Of course, the deletion index and D100 will correlate to a large extent, however, only D100 actually assesses large deletions, which is the main focus of the manuscript. To support the claims of the manuscript, all main figures should therefore mainly focus on D100 and not the deletion index.*

**Response:** Thanks for your suggestions. We have clarified “deletion indexes” in the Method session-Nanopore sequencing data analyses. After careful consideration of all the reviewers' and editor’s perspectives, we decide to show deletion indexes data instead of D100 in the central part of this manuscript. The deletion indexes represent the mean values of all types of deletions, not just deletions of over 100 bp for D100. We observed more frequent large deletions in T cells and HSPCs, the widely used cell types in clinical therapy. Since one allele with a more considerable mutation will lead to an increase in deletion index but no change in D100, the metric of DI is more informative than D100. We propose the use of deletion indexes to assess the safety of gene therapy products. Publication of our manuscript will invite more meaningful discussions on this critical issue.

*Response 2: Per request, we have now re-analyzed our data, following the norm of this
field. In theory, the deletion index also contains all small/medium-sized indels. In reality,
the vast majority of alleles with large deletions are D100-500. Even though
small/medium-sized indels occurred more often, it does not significantly impact the DI
values. In addition, deletions of different sizes are not independent events. In other
words, more medium-sized deletion correlated with more large deletions, as shown in
Fig S2. As such, DI correlated very well with D100, as shown in Fig 2e, 3e, and S8c.
The use of the DI metric has its merit, for it unnecessities the arbitrary threshold used
in defining how large is large enough to be considered a large deletion. For example,
one may consider 50-bp del, and another prefers 100-bp deletion.
And we considered deletions larger than 100 bp (D100) as large deletions and found a
good correlation of DI and D100 in Fig. S8. The different sizes of deletions were also
analyzed and showed in Fig. S2a-c. We have also added the statement in the Results
session as follows:
“To quantify the levels of large deletions precisely, we used the deletion index, whereas
previous studies defined removing fragments of over 100 bp (D100) as large deletions.
Therefore, we extended the analysis by calculating alleles with deletions of over 100,
500, 1000, 1500, or 2000 bp (Fig. S2a-c and S2d-f). We found that over 80% of large
deletions were 100-1000 bp in length, while D2000 was 0.1% or lower (Fig. S2a-c).
Furthermore, even though D100 was more sensitive than deletion index (DI) in
assessing large deletions, deletion indexes correlated excellently with D100 (Fig. 2e,
3e, and S8c). We thus decided to report DI in detail and also summarized D100 data for
comparison.”

see answer to 1e)*

**Response:** Thanks for the comments. We have answered the question in 1e.

*Response 3: We agree with your professional concern. The data with HDR gene editing
were all analyzed with both HDR and NHEJ alleles. We have clearly stated the analysis
process in the manuscript especially for the large fragment HDR knock-in in EEF2
locus. To investigate the quantification based only on reads without HDR, we have
removed the HDR reads as shown in Fig. S5 and compared the deletion indexes
between alleles with HDR or without HDR. And we found there were no significant
differences in deletion indexes. The detailed information was stated in the Results
session and Fig. S5: “At the EEF2 locus, rather than a short fragment knock-in, AAV6
mediated a mNeonGreen reporter insertion. To determine whether the HDR alleles in
the dataset artificially decrease the deletion indexes, we analyzed data after depletion
of HDR alleles (Fig. S5a and S5b). We found no significant differences between the
reads with HDR alleles and reads without HDR alleles (Fig. S5c and S5d). These data
suggest that our analytical strategy precisely captures the deletion levels.”

No further comments.*

*Response 4: Compared with T cells and HSPCs, large deletions in gene-edited iPSCs
were low and close to the background in wild-type iPSCs. We have double-checked our
manuscript and avoided overstating our data. Changes in iPSCs were largely
insignificant because of the low background deletions. These seemingly negative data
are essential for the field of iPSCs, given their expanding applications in clinical cell
therapy.
We found relatively low and near the unedited WT cells background in large deletion
after RNP gene editing, which is different from other published finding (Weisheit I et
al., Cell Reports, 2020). We have performed more experiments to address issue
including cell cycle analysis. This part has been considerably modified as follows: “In
the above studies, we observed considerably lower deletions in iPSCs compared to T
cells and HSPCs. Our iPSC-RNP-editing results are seemingly at odds with a previous
study using plasmid editing in which iPSCs with high-level Cas9 expression were drug selected.
We asked if cell death after electroporation of RNPs contributed to biased
results in the three types of cells. We counted cell numbers two days after transfection
relative to unmanipulated counterparts as a surrogate indicator of cell survival. No
significant differences in viabilities were observed (Fig. S3a). We further investigated
if changes in the cell cycle played a part. We profiled the cell cycle using Pyronin Y
and Hoechst 33342 co-staining one day after RNP delivery (Fig. S3b and S3c). We
observed no significant differences in G0/G1, S, and G2/M phases in HSPCs (Fig. S3d),
while a slight increase of T cells in the S phase and iPSCs in the G2/M phase. However,
we noted considerably more iPSCs in the G2/M phase than T cells or HSPCs (~50% vs.
20%). This striking distinction might have partly contributed to ~5-fold lower deletions
in edited iPSCs since the HDR is the prevailing pathway in cycling iPSCs. Together,
significant low-level deletion mutagenesis in iPSCs likely results from their intrinsic
nature and transient low-level intracellular Cas9.”

Regarding this issue, I disagree with the authors on three different points:
First, in contrast to what the authors state above, recent publications have indeed shown high levels of large deletions in iPSCs, mouse and human cell lines (e.g. Weisheit et al., Nature Protocols, 2021, or Kosicki et al., Nature Biotech, 2018) also after RNP-based CRISPR editing. Therefore, the sentences…
p.9, line 41 “Our iPSC-RNP-editing results are seemingly at odds with a previous
study using plasmid editing in which iPSCs with high-level Cas9 expression were drugselected.”
p.9, line 6 “As such, we speculate that RNP editing may also decrease large deletion incidents.”
…should be modified or entirely removed from the text*

**Response:** We have removed the statements.

*Second, the authors state that “large deletions in gene-edited iPSCs were low and close to the background in wild-type iPSCs” (see Fig. 1d, the deletion index is close to 0! for iPSCs). However, although they can hardly detect large deletions in iPSCs in the first place, the authors still try to find factors that influence the occurrence of large deletions in iPSCs. This is not very informative. I therefore suggest to remove all iPSC data, (see also comment on Fig. 1d in third point), from the manuscript!*

**Response:** CRISPR editing of iPSCs has broad applications in cell and gene therapy; investigation into the potential of large deletions in gene-edited iPSCs will advance the fields of iPSC-based therapies. Thus, we believe that removing all iPSC data is not a prudent choice. *Third, the low level of deletions in iPSCs is in contrast to published studies that show up to 40% of edited clones having deletions or other on-target effects (Weisheit et al., Cell Reports, 2020). As already mentioned, it remains unclear if this discrepancy is due to lower levels of deletions in their cells or their inability to reliably detect them all. Again, this discrepancy needs to be mentioned and discussed clearly, further investigated by experimental data, or completely removed from the manuscript! Telling the genome editing field that large deletions do not occur in iPSCs (although published data clearly shows the opposite) is not helpful, but might rather be a wrong “all-clear signal”!*

**Response:** We have discussed this discrepancy in the discussion part as follows: “This discrepancy might attribute to the different approaches for delivering editing components and data analysis methods. We conducted long-range PCR and nanopore sequencing to assess large deletions. Although we showed no apparent large deletions in gene-edited iPSCs compared with wildtype cells, we still found one clone with large deletion among the 52 iPSC single-cell clones. Thus, gene-edited iPSCs may need rigorous analysis of on-targets to avoid unintended outcomes.”

*Response 5: We have found a minor effect of NHEJ or MMEJ inhibitors on indel
frequency in many loci, because NHEJ blockade is offset by MMEJ repair, which leads
to more short deletions. To demonstrate the efficacy of inhibitors, we have presented
one typical editing outcome with one predominant +A type NHEJ and a -25bp type
MMEJ (the micro homology is CAGGAAG) at EEF2 locus. We found a significant
decrease in +A NHEJ frequencies and a significant increasing in -25bp MMEJ
frequencies with either M3814 or NU7441 treatment (Fig. S9). We then blocked the
MMEJ pathway with Olaparib. MMEJ inhibition reduced the NHEJ repair pathway but
had no significant effect on MMEJ mutations (Fig. S9). But we did not find any
significant changes of MMEJ alleles when treated with Olaparib. And our recent study
has also shown that Olaparib cannot reduce MMEJ mutations (Ya-Wen Fu, et al.,
Nucleic Acids Research, 2021). Unfortunately, there are no potent MMEJ inhibitors
available on the market. Therefore, for the time being, the effects of MMEJ can not be
meticulously examined. However, our data still suggest that HDR and NHEJ play
dominant roles in curtailing DI. However, this conclusion does not suggest that MMEJ
does not impact the outcomes of large deletions. In contrast, if MMEJ is totally blocked,
more damages to chromosomes will ensue.

First, the resolution of Figure S9a is too low for proper inspection.*

**Response:** We have uploaded all raw pictures with high resolution individually as a backup.

*Second, a comment on sentence in the answer above: “MMEJ inhibition reduced the NHEJ repair pathway but had no significant effect on MMEJ mutations (Fig. S9).” In Figure S9b, there is no effect on neither NHEJ, nor MMEJ repair by Olaparib (which was supposed to inhibit MMEJ). This clearly shows that this inhibitor does not have any effect, the data should be removed from the manuscript.*

**Response:** We have removed the data.

*Response 6: We agree that adding double-stranded DNA fragments poses a potential risk
of random integrations into the genome. We have now discussed this point in the
Discussion session as follows: “However, the dsODN pose a risk to random insertion
at dsDNA breaks induced by replication or other stresses. Further investigation will
gain more insight into this potential concern.”

Add reference (e.g. to GUIDE-seq protocol). Rest is okay.*

**Response:** We have added the reference.

*Minor issues:

Response 1: We have discussed this phenomenon in the discussion part. The detailed
information was stated in response to major issue 1.

From our understanding, the authors addressed this point with a sentence on p.10, line 8
“Loss-of-heterozygosity may arise in the target locus was not investigated [27].”
Please make this sentence clearer, e.g. “…occurrence of loss-of-heterozygosity was shown in a previous study investigating deleterious on-target effects after CRISPR editing[27], but was not investigated in our experiments...”*

**Response:** We have changed the statement.

*Response 5: This statement was confusing. To clarify, we have deleted “<1%” and
changed the statement to “We first calculated the area under the curve using ImageJ to
define significant deletions.”

Please clarify the expression “area under the curve”. Also, what is “mean depth” in the following sentence? (p.3, line 8)*

**Response:** We have changed the statements and clarified “mean depth” in the Method session -- Nanopore sequencing data analyses.

*Response 9: We have uploaded all high-resolution images in the revised version.

In the file we received, the resolution seemed to have gotten worse than it was last time. Same applies e.g., for Figure 3a and S9a.*

**Response:** The low resolution may result from the format conversion of the file. We have uploaded all raw pictures with high resolution individually as a backup. *13. p.7, Line 13: What do the authors mean by "more significant small deletions"?
Blocking NHEJ rather decreases small deletions?

Response 13: Blocking the NHEJ pathwy increases the MMEJ mediated deletions. To
clarify, we have changed the statement to “NHEJ inhibition delays the DSB repair,
leading to more MMEJ mediated deletions (Fig. S9) and large disruptions”.

This sentence is confusing, since MMEJ is also a DSB repair pathway. I suggest to make this clearer.*

**Response:** We have changed the statement to “NHEJ inhibition leads to more MMEJ mediated deletions (Fig. S9) and large disruptions”. *Further new comments:

1. p.3, line 12. “The deletion indexes analyzed by Samtools and ImageJ showed an excellent linear correlation (R2 = 0.98) (Fig. 1b and S1c).”

Which data in Figure 1b is this sentence referring to?*

**Response:** We have deleted “Fig. 1b” in the sentence.

*2. p.8, line 28 “The large fragment removal occurred after CRISPR-Cas9 induced gene editing in mouse embryonic stem cells and human embryos [10, 11].”

What is meant by “large fragment removal?”*

**Response:** We have changed “large fragment removal” to “large fragment deletion”. *2. p.8, line 58: This sentence does not make sense.*

**Response:** We have deleted this sentence.
